# A spectroscopic test suggests that fragment ion structure annotations in MS/MS libraries are frequently incorrect

**DOI:** 10.1038/s42004-024-01112-7

**Published:** 2024-02-14

**Authors:** Lara van Tetering, Sylvia Spies, Quirine D. K. Wildeman, Kas J. Houthuijs, Rianne E. van Outersterp, Jonathan Martens, Ron A. Wevers, David S. Wishart, Giel Berden, Jos Oomens

**Affiliations:** 1 Radboud University, Institute for Molecules and Materials, FELIX Laboratory, Toernooiveld 7, 6525ED Nijmegen, The Netherlands; 2 Department of Laboratory Medicine, Translational Metabolic Laboratory, Radboud University Medical Center, Geert Grooteplein Zuid 10, 6525GA Nijmegen, The Netherlands; 3 Departments of Computing Science and Biological Sciences, University of Alberta, Edmonton, AB Canada; 4 van ‘t Hoff Institute for Molecular Sciences, University of Amsterdam, Science Park 904, 1098XH Amsterdam, The Netherlands

**Keywords:** Mass spectrometry, Bioanalytical chemistry, Infrared spectroscopy, Cheminformatics

## Abstract

Modern untargeted mass spectrometry (MS) analyses quickly detect and resolve thousands of molecular compounds. Although features are readily annotated with a molecular formula in high-resolution small-molecule MS applications, the large majority of them remains unidentified in terms of their full molecular structure. Collision-induced dissociation tandem mass spectrometry (CID-MS^2^) provides a diagnostic molecular fingerprint to resolve the molecular structure through a library search. However, for de novo identifications, one must often rely on in silico generated MS^2^ spectra as reference. The ability of different in silico algorithms to correctly predict MS^2^ spectra and thus to retrieve correct molecular structures is a topic of lively debate, for instance in the CASMI contest. Underlying the predicted MS^2^ spectra are the in silico generated product ion structures, which are normally not used in de novo identification, but which can serve to critically assess the fragmentation algorithms. Here we evaluate in silico generated MS^n^ product ion structures by comparison with structures established experimentally by infrared ion spectroscopy (IRIS). For a set of three dozen product ion structures from five precursor molecules, we find that virtually all fragment ion structure annotations in three major in silico MS^2^ libraries (HMDB, METLIN, mzCloud) are incorrect and caution the reader against their use for structure annotation of MS/MS ions.

## Abstract (web summary)

Collision-induced dissociation tandem mass spectrometry offers to resolve molecular structures through library searches, however, for de novo identifications, one must often rely on in silico*-*generated spectra as reference. Here, the authors evaluate in silico generated MS^n^ product ion structures by comparison with spectroscopically established structures and find that for 36 randomly selected MS/MS product ions, the vast majority of annotations in three major libraries are incorrect, primarily due to unaccounted for cyclization rearrangements.

## Introduction

Owing to its high sensitivity and resolution, mass spectrometry (MS) has become indispensable in the detection and identification of molecular species in complex mixtures, e.g., in metabolomics and many other small-molecule applications^1^. Modern MS instruments detect and resolve thousands of molecular compounds in a sample in a matter of just seconds. However, most features detected in untargeted MS analyses are not identified in terms of their full molecular structure. While the resolving power is usually sufficient to assign a unique chemical formula to the detected ions, the mass value alone gives little information on the arrangement of the atoms within the molecule. Consequently, definitive identification of the molecular structure remains challenging as it requires structural and stereoisomers corresponding to the same mass-to-charge ratio (*m/z*) to be distinguished.

Tandem mass spectrometry (MS/MS = MS^2^) is commonly used to advance structure annotation beyond the chemical formula. Collision-induced dissociation (CID) of a precursor ion selected in the first MS stage produces a structurally diagnostic fragmentation pattern in the second MS stage. To delineate a molecular structure for the precursor ion, this fragmentation pattern is compared against the entries in MS/MS spectral libraries^2^. Many application-specific libraries exist^2–4^, e.g. for metabolites, agrochemicals, toxicological substances, drug compounds, etc. However, even taken together, MS/MS reference libraries cover only a minute fraction of chemical space, estimated as perhaps 1% or so^3,5–7^. For de novo identifications beyond these ‘known unknowns’, in silico strategies to identify structures have been developed. High-level quantum-chemical computation of MS/MS spectra^8^ is developing, but far too costly to screen large numbers of candidate structures. Much faster methods originally relied mostly on rule-based^9,10^ and combinatorial^9,11^ fragmentation approaches^7^, while more recently, these heuristic models are being updated with strategies involving elements of machine learning^12–16^.

**Fig. 1:**
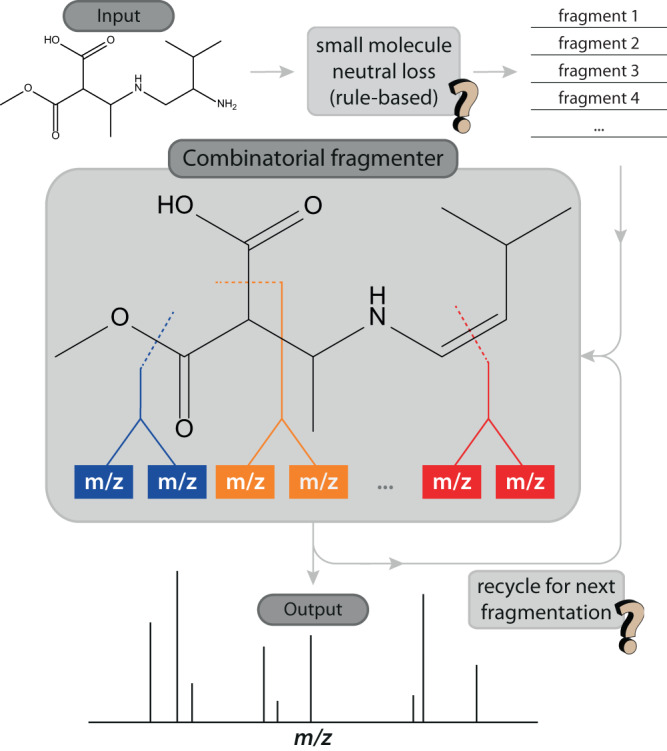
Simplified schematic showing the combinatorial fragmentation algorithm. Combinatorial fragmentation is used to generate in silico MS/MS spectra (figure loosely based on Wolf et al.^11^). The question marks indicate steps where rearrangements can occur that are not accounted for in the algorithm.

The combinatorial approach (Fig. 1) to predict MS/MS spectra is still widely in use and underlies some of the more recent machine-learning strategies^13,14^. A large compound database is screened for entries with the accurate mass (MS^1^) of the unknown query molecule. For each hit, a quick rule-based algorithm first determines possible small neutral losses (H_2_O, NH_3_, etc.), which are common in MS/MS and often occur early on in the CID breakdown cascade. A list of possible fragment *m/z* values is then generated in silico by breaking each bond in the molecule (excluding X-H bonds). The resulting combinatorial fragment *m/z*’s are fed back into the in silico fragmenter for a second (and third) round of fragmentation, forming so-called fragmentation trees (not to be confused with experimental MS^n^ spectral trees). Each type of chemical bond represents a preset bond dissociation energy, so that the probability of generating a specific *m/z* fragment—and hence its relative intensity in the MS/MS spectrum—can be quantified from the summed bond dissociation energies along the fragmentation tree ending up at that *m/z*. The resulting in silico MS/MS spectrum is then matched against the query MS/MS spectrum.

A caveat involves the neglect of possible structural rearrangement occurring upon bond dissociation^7,10^. Rearrangement constitutes the replacement of broken bonds with new ones. In the in silico fragmenter, rearrangement would thus lead to a different set of combinatorial fragments being generated upon a next round of fragmentation. Neglecting rearrangement, as indicated by the question marks in Fig. 1, may thus lead to fragment ion *m/z* values in the in silico MS/MS spectrum that are incorrect. Especially rearrangements occurring early on in the fragmentation tree may strongly affect the entire downstream tree.

Commonly used in silico fragmenters include MetFrag, which generates MS/MS spectra for the METLIN (https://metlin.scripps.edu) database^17,18^ and Competitive Fragmentation Modeling (CFM-ID)^9,15^ which provides spectra for the Human Metabolome Database (HMDB) (https://hmdb.ca)^19–23^. mzCloud implements heuristic approaches by combining general fragmentation rules with fragmentation mechanisms published and (partly) relies on manual evaluation^24^. mzCloud (https://www.mzcloud.org) also uses ab initio and density functional theory (DFT) quantum-chemical calculations, assigning the lowest-energy isomer found as the fragment ion structure and thus ignoring the kinetic aspects of the CID reaction.

The question that arises is whether the annotated structures of the MS/MS fragment ions (that are often included in in silico MS/MS libraries) are indeed correct. Even though these structural annotations are not used directly in MS/MS spectral analysis, the in silico MS/MS spectra that are compared with the experiment are derived from the combinatorial algorithm that involves these structures. An estimate of the reliability of these structures is, therefore, an indirect measure for the reliability of predicted MS/MS spectra. Incorrect structures are expected to degrade the quality of the predicted MS/MS spectrum, especially in the lower mass range, where ions occur that are formed upon cleavage of more than one bond. Deviations are, therefore also expected to be more severe at higher-energy CID settings.

Beyond their role in predicting MS/MS spectra, annotated CID-fragment ion structures may serve other purposes as well. In MS^n^ libraries, the MS^n^ spectrum may be used to identify the MS^n−1^ ion structure. As such, the annotated structures increase the number of entries (molecular structures) in the database, including compounds that may not be available as reference standards^25^. An example of making use of this approach is implemented in METLIN and mzCloud, where a precursor-independent similarity search can be performed that can identify molecular substructures based on annotated fragment structures. In addition, annotations consolidate the internal consistency of the database, as families of structurally similar compounds become connected through shared substructures at deeper stages of the fragmentation tree^26^.

To address the question at hand, we verify a selection of annotated structures in various MS/MS libraries by infrared ion spectroscopy (IRIS). IRIS records an infrared (IR) spectrum for a mass-isolated CID MS^n^ ion in an ion trap mass spectrometer. Structure determination is then achieved by comparison of the experimental IR spectrum to theoretical IR spectra for candidate structures computed at the density functional level of theory (DFT). In this work, we evaluate the fragment ion structures for a total of 36 MS^n^ ions derived from five precursor molecules, all listed as small-molecule human metabolites. Although this is admittedly a very small sample dataset, it serves as a random selection and ought to give us some insight into the reliability of fragment ion structure annotations in common mass-spectral libraries. We find that the structure annotation of almost all fragment ions investigated is incorrect, which can often be attributed to the neglect of cyclization reactions (METLIN and HMDB) or to an incorrectly selected cyclized product ion (mzCloud). As an indication of the importance of rearrangements in CID MS/MS reactions, a large body of work has been reported on the dissociation reactions of small protonated peptides, employing theoretical and experimental methods in fundamental ion chemistry, including ion spectroscopy^27^. Furthermore, there are indeed indications that predicted MS/MS spectra are less accurate at higher collision energy^28^, possibly exposing the effects of rearrangements occurring in between two bond cleavages, which are not accounted for in the in silico modeling.

## Results and discussion

### Workflow

**Fig. 2:**
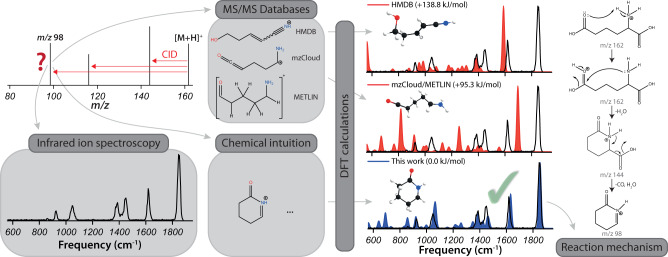
Workflow to establish the structure of CID MS^n^ fragment ions. The MS^n^ fragment ion of interest is generated by CID and isolated in the ion trap mass spectrometer, where its IRIS spectrum is recorded. DFT geometry optimizations and vibrational frequency calculations are performed for the structures listed in MS/MS libraries as well as for alternative structures suggested by chemical intuition. The structure providing the predicted spectrum with the best match to experiment is annotated as the actual MS^n^ fragment ion structure. Based on this identification, we propose plausible reaction paths from precursor to product ion.

IRIS spectra were recorded for 36 CID-fragment ions derived from five protonated precursors (α-amino-adipic acid, urocanic acid, citrulline, homocitrulline, arginine). Investigated species include MS^2^, MS^3^ and a few MS^4^ ions. Experimental IRIS spectra were manually compared with predicted IR spectra. In cases where multiple conformers with similar hydrogen bonding interactions are computed to be within 6 kJ mol^−1^, a Boltzmann-weighted average of the predicted spectra was taken. The degree of spectral matching was determined qualitatively by visual inspection, and the structure providing the best match was assigned as the actual fragment ion structure. Based on this structure, we suggest plausible reaction pathways; transition-state calculations needed to confirm these mechanisms are beyond the scope of the present study. An overview of the workflow is depicted in Fig. 2.

All experimental spectra, comparisons with computed spectra, and the structural identifications proposed are shown in Supplementary Figs. S1–S33. Below, we highlight selected examples and discuss different aspects of structural identification. Figures 3–5, 7, and 9 in this section summarize our spectroscopy-based structure determinations compared against annotations from in silico MS/MS libraries. Suggested reaction pathways from precursor to product ions are provided in Supplementary Figs. S36–S61.

**Fig. 3:**
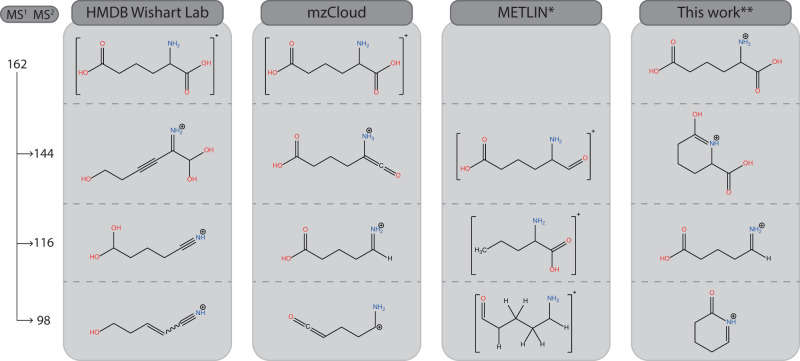
MS^2^ spectral tree for protonated α-amino-adipic acid with structural annotations. Structures derived in this work from the IR spectra of the fragment ions are compared with those listed in three databases. * For the METLIN structures, a proton must be added to obtain an ion of the correct *m/z* value. ** Annotations in this column are based on the spectroscopic analysis shown in Supplementary Figs. S1–S4.

**Fig. 4:**
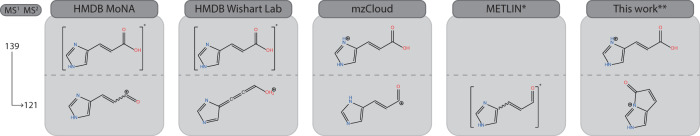
Spectral tree for protonated urocanic acid with structural annotations. The bicyclic structure identified by ion spectroscopy is not reproduced in any of the databases. * For the METLIN structure, a proton must be added to obtain an ion of the correct *m/z* value. ** Annotations in this column are based on the spectroscopic analysis shown in Supplementary Figs. S5–S6.

**Fig. 5:**
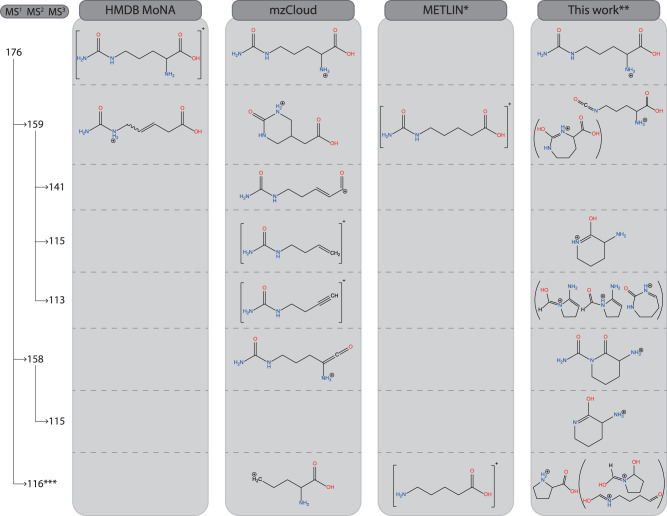
MS^n^ spectral tree for protonated citrulline with structural annotations. Structures from the databases are contrasted against spectroscopically identified structures. Structures in brackets are tentative. * For METLIN structures, a proton must be placed at one of the carbon atoms to obtain an ion of the correct *m/z* value. ** Annotations in this column are based on the spectroscopic analyses shown in Supplementary Figs. S7–S15. *** A further analysis of the *m/z* 116 product ion from citrulline and from arginine is presented in Supplementary Fig. S34 and Supplementary Note 1.

### α-amino-adipic acid—cyclization rearrangements are common

As an example of the typical workflow, the results for α-amino-adipic acid (α-AAA) are shown in Fig. 2. Upon CID, the MS^2^ spectrum of protonated α-AAA reveals three prominent fragment ions at *m/z* 144, 116, and 98. These MS^2^ ions were consecutively mass-isolated, and their IRIS spectra were recorded, see Supplementary Figs. S2–S4. The IRIS spectrum of the *m/z* 98 fragment is shown as an example in Fig. 2, along with computed spectra for the annotated structure for this fragment ion taken from the three databases. The elemental composition of the *m/z* 98 fragment was established as C_5_H_8_NO^+^ using FTICR-MS (see Supplementary Table S1) and is presumably formed by the neutral loss of CO + 2H_2_O. The HMDB^19–23^ and mzCloud^26^ libraries give the correct chemical formula for this fragment. The annotation in METLIN^17,18,25^ is ambiguous (see Fig. 2): at the aldehyde terminus, either a CC double bond is missing or some, but not all, hydrogens are implicit. To arrive at the correct *m/z* value, we assume that there is a double bond, forming a ketene moiety; the formal charge is then at the carbon atom adjacent to the amine, so that the METLIN annotation is identical to the one in mzCloud.

Comparisons of experimental and theoretical spectra in Fig. 2 shows that the spectra calculated for the annotations in the libraries deviate substantially from the measured spectrum. Most notably, the strong band observed at 1850 cm^−1^ is missing for the HMDB structure and shifted for the METLIN/mzCloud structure. We, therefore, devised new structures by chemical intuition and calculated their IR spectra (see Fig. 2 and Supplementary Fig. S4). The N-protonated 6-oxo-1-piperideine ion provides a good match with the experimental spectrum, attributing the 1850 cm^−1^ band to the carbonyl stretch mode. With this structure for the *m/z* 98 fragment ion, a reaction mechanism may be proposed involving proton migration upon activation and nucleophilic attack, driving a cyclization and concomitant expulsion of H_2_O. Subsequently, the carboxylic acid moiety detaches from the ring and (probably) leaves as H_2_O + CO. These processes are not uncommon in gas-phase ion chemistry, although a detailed confirmation of the reaction path requires transition-state calculations.

Figure 3 summarizes all IRIS-based CID-fragment ion annotations for α-AAA, based on the experiment-versus-theory spectral comparisons in Supplementary Figs. S1–S4. One notices that all structures proposed by the databases are linear, as implied by the in silico algorithms that were used to derive these structures. However, two out of three spectroscopically established fragment structures are cyclized products.

### Urocanic acid—another example of cyclization upon CID

As another example of facile cyclization upon CID, Fig. 4 shows results for the water loss fragment ion at *m/z* 121 of protonated urocanic acid, which is the main CID fragment. Our IR spectroscopic analysis shown in Supplementary Fig. S6 unambiguously demonstrates that cyclization occurs to form a bicyclic structure (see Fig. 4). None of the annotations in the MS/MS libraries predict this cyclized structure and instead contain only the original imidazole ring of the precursor. It is interesting to note that MoNA and Wishart Lab sources in the HMDB give different annotations (Splash Key identifiers for the MS/MS spectra used are given in Supplementary Table S6). The MoNA data are based on an experimental MS/MS spectrum to which annotations have been added. The Wishart Lab MS/MS spectrum and its structural annotations are predicted by CFM-ID.

### Citrulline—lowest-energy isomer is not necessarily correct structure

For citrulline, several MS^2^ as well as MS^3^ fragments were explored, using the MS^n^ capabilities of the ion trap instrument. Figure 5 presents the MS^n^ spectral tree including the annotations from the libraries and from our IR spectroscopic identifications. The underlying experimental IRIS spectra and their matching against DFT computed spectra for hypothesized fragment ion structures is given in Supplementary Figs. S7–S15. The HMDB and METLIN libraries only contain MS^2^ spectra, whereas mzCloud also includes fragments of MS^3^ and higher.

**Fig. 6:**
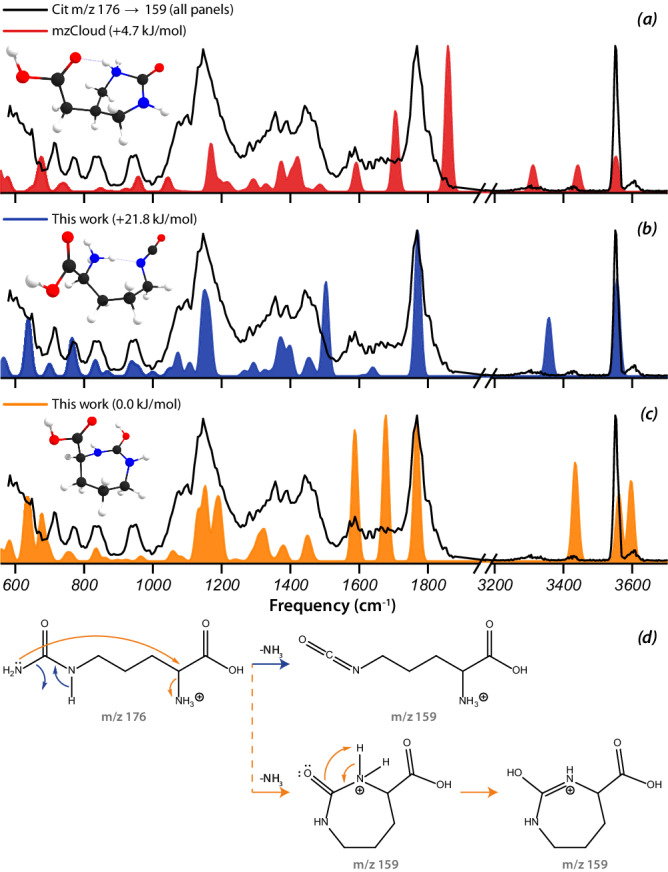
IRIS spectrum of the *m/z* 159 MS^2^ ion from [citrulline + H]^+^. The measured spectrum is shown as the black trace in **a**–**c**. The spectrum predicted for the annotation in mzCloud deviates substantially from the measured spectrum (**a**). The non-cyclized isomer provides a reasonable match for most bands observed experimentally (**b**). To account for the experimental intensity near 1550–1700 cm^−1^ and at 3600 cm^−1^, a minor contribution of the 7-membered ring structure is invoked (**c**). Proposed reaction mechanisms leading to these fragment structures (**d**).

Among the various libraries, mzCloud uniquely employs quantum-chemical calculations to annotate the structure of MS^n^ fragment ions. Calculations are performed for multiple candidate structures and the lowest-energy isomer is proposed as the actual product ion structure. As such, this strategy considers only thermodynamic aspects of the CID reaction and ignores possible kinetic effects. This may lead to incorrect annotations, as is illustrated here for the *m/z* 159 fragment. mzCloud provides PM6 calculations for various candidate structures, giving the 6-membered ring structure shown in Fig. 5 as the global minimum. Figure 6a compares the experimental IRIS spectrum of the *m/z* 159 fragment ion with the B3LYP-calculated spectrum of this structure, revealing a clear mismatch. Most notable is the poor match between the computed carbonyl stretch band at 1840 cm^−1^ and the strong experimental band at 1770 cm^−1^. In contrast, the computed spectrum for a linear isomer formed by direct NH_3_-loss from the urea moiety, shown in Fig. 6b, provides a reasonable match. The carbonyl stretch as well as the strong feature around 1000–1200 cm^−1^ and the series of weaker bands between 600 and 1000 cm^−1^ overlap convincingly. The mismatch near 1500 cm^−1^ is due to an amine NH bending vibration, which often show small shifts in harmonic frequency calculations^29–31^. The OH stretch mode near 3550 cm^−1^ is closely reproduced, whereas the NH stretch band computed near 3360 cm^−1^ appears broadened and redshifted, which is likely a consequence of the shared-proton character of the N-H∙∙∙N moiety in combination with the room-temperature environment of the experiment^32–34^.

The experimentally observed absorption at 3600 cm^−1^ is not accounted for in the computed spectrum, nor is the plateau between 1550 and 1700 cm^−1^. It appears that a fraction of the *m/z* 159 ion population adopts the 7-membered ring structure shown in Fig. 6c, which corresponds to the global minimum at our level of theory, 5 kJ mol^−1^ lower than the 6-membered ring global minimum structure of mzCloud. From a kinetic point of view, the 7-membered ring appears more plausible since it can form through NH_3_-loss from the urea moiety with concomitant nucleophilic attack of the amino nitrogen onto the urea carbon atom (see Fig. 6d). In contrast, formation of the 6-membered ring would require additional displacement of the amino group along the alkyl backbone.

The IRIS spectrum suggests that the global minimum 7-membered ring structure is a minor contributor to the total ion population, with the linear isomer at a relative energy of 22 kJ mol^−1^ being the dominant contributor. We speculate that pathways leading to rings of larger sizes face increasing entropic barriers^35^. The PM6 calculations in mzCloud place this linear isomer 190 kJ mol^−1^ above the 6-ring structure. This example shows that the lowest-energy structure is not necessarily the actual fragment structure. This is not uncommon in CID reactions, as for instance, illustrated by the oxazolone structure adopted by b_2_ sequence ions of many protonated peptides^36,37^, which does not correspond to the global minimum, as confirmed in various ion spectroscopy studies^27,38–42^.

### Homocitrulline—MS^n^ ions of same *m/z* may have different structures

**Fig. 7:**
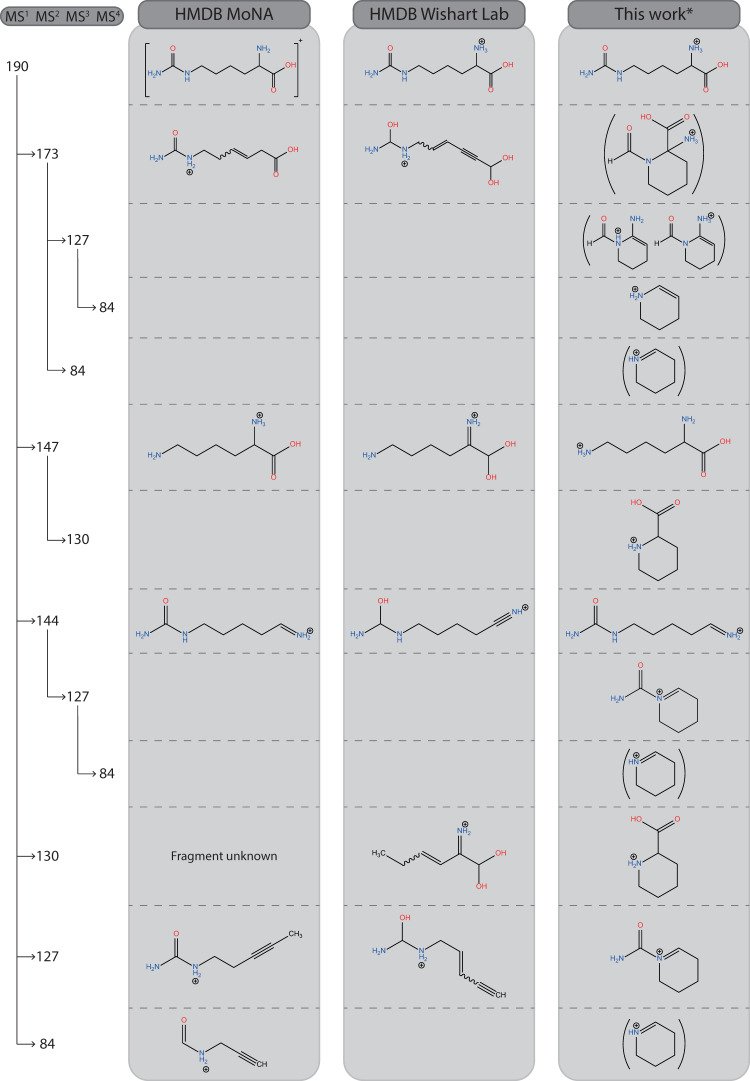
MS^n^ spectral tree for [homocitrulline + H]^+^ with annotations. HMDB library annotations are compared with structures identified through ion spectroscopy in this work. Annotations in brackets are tentative. * Annotations in this column are based on the spectroscopic analysis shown in Supplementary Figs. S16–S24.

For homocitrulline, MS^2^ as well as MS^3^ and MS^4^ fragments were investigated, as listed in the MS^n^ spectral tree in Fig. 7, with annotations from the HMDB library and from our IR spectroscopic identifications. The HMDB libraries only contain MS^2^ spectra; homocitrulline is not included in mzCloud.

In MS^n^ spectra with *n* ≥ 3, fragments of the same *m/z* value may be generated via different MS^n^ paths, i.e., involving different MS^2^ intermediates. It appears obvious in such cases that an identical *m/z* value does not necessarily imply that these fragments correspond to the same structure. However, strategies that assign by definition the lowest-energy isomer give identical annotations to such fragments.

**Fig. 8:**
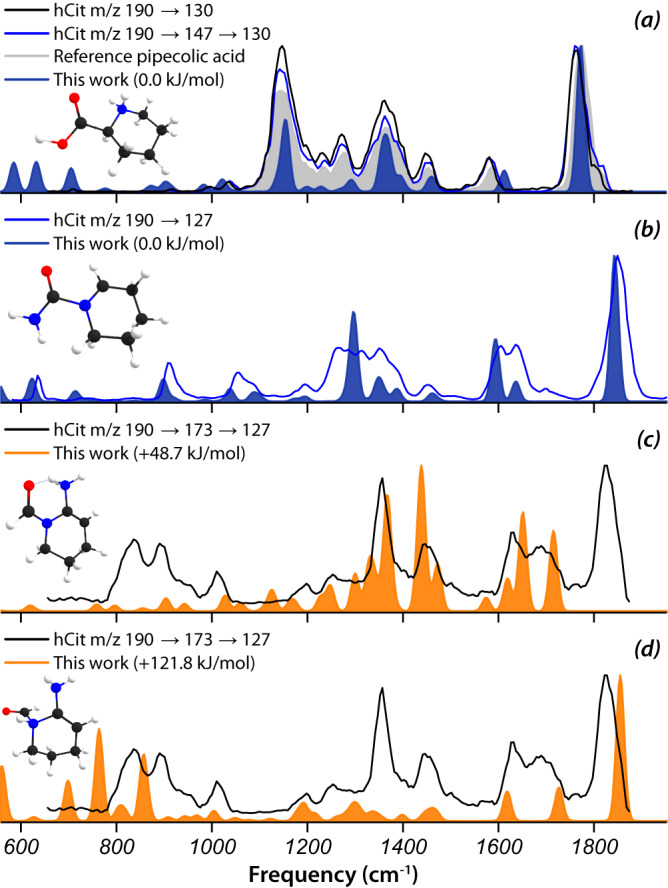
The *m/z* 130 fragment ion of homocitrulline generated via different MS^n^ pathways. **a** IRIS spectra of the *m/z* 130 CID product ion from [hCit + H]^+^, generated either directly (MS^2^) or via the *m/z* 147 intermediate (MS^3^). Both *m/z* 130 ions share the same spectrum and hence the same structure, which is identified as protonated pipecolic acid (computed spectrum shaded blue and reference IRIS spectrum shaded gray). **b** IRIS spectrum of the *m/z* 127 fragments produced directly (MS^2^) from the *m/z* 190 precursor or (**c**, **d**) via the *m/z* 173 intermediate (MS^3^). Clearly, different species are generated: MS^2^ produces the 1-piperideine-N-carboxamide ion (**b**), while MS^3^ is suggested to produce two protonation isomers of 2-amino-2-piperideine-N-aldehyde (**c**, **d**).

Protonated homocitrulline, [hCit+H]^+^ at *m/z* 190, shows an MS^2^ fragment at *m/z* 130 that can also be generated in an MS^3^ experiment via the *m/z* 147 intermediate MS^2^ ion. IRIS spectra of the *m/z* 130 ion produced via either the MS^2^ or the MS^3^ pathway are identical, see Fig. 8a. Hence, the two ions possess the same structure, which we identify as protonated pipecolic acid based on a comparison with its predicted IR spectrum. Moreover, a physical reference standard of pipecolic acid is available and its IRIS spectrum coincides closely with those of the MS^2^ and MS^3^ product ions. The HMDB predicts a linear dienol iminium ion structure for the MS^2^ ion (Fig. 7). Pipecolic acid was identified previously as the *m/z* 130 MS^2^ ion of protonated L-lysine^43^.

The *m/z* 127 fragment ion of [hCit+H]^+^ can also be generated via two distinct MS^n^ routes, either directly in MS^2^ or indirectly in MS^3^ with the *m/z* 173 ion as intermediate. In this case, IRIS spectra of the MS^n^ ions generated through different pathways are clearly distinct, indicating that they do not share the same molecular structure. For the MS^2^ fragment ion, the computed spectrum for the 1-piperideine-N-carboxamide ion provides a convincing match, see Fig. 8b. The IRIS spectrum of the *m/z* 127 MS^3^ fragment ion formed via *m/z* 173 is more difficult to assign. We suspect that two isomeric ions co-exist, as none of the computed spectra reproduces the number of bands observed (see Supplementary Fig. S22). We tentatively propose a mixture of two protomers of 2-amino-2-piperideine-N-aldehyde, protonated either on the amine or on the piperideine nitrogen, shown in Fig. 8c, d.

### Arginine—a comparison with the ion chemistry literature

**Fig. 9:**
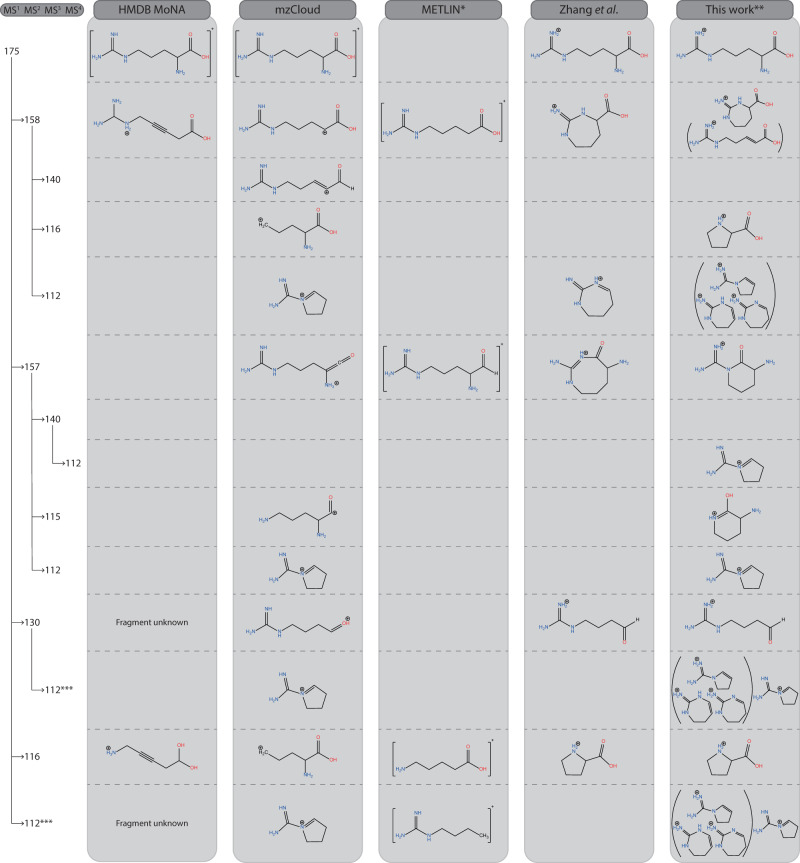
MS^n^ spectral tree of protonated arginine with annotations. Annotations proposed by the MS libraries, Zhang et al.^44^, and ion spectroscopy (this work) are compared. Structures in brackets are tentative assignments. * For METLIN structures, a proton must be placed at one of the carbon atoms to arrive at an ion of the correct *m/z* value. ** Annotations based on the spectroscopic analysis shown in Supplementary Figs. S25–S33. *** A more detailed analysis of the *m/z* 112 fragment ion is presented in Supplementary Fig. S35 and Supplementary Note 2.

The vast ion chemistry literature documents numerous studies proposing MS^n^ structure annotations based on manual interpretation of MS^n^ spectra. For protonated arginine, Zhang et al.^44^. employed high-resolution ESI-MS and HCD-MS^2^ on a high-resolution Orbitrap platform to derive product ion structures and reaction mechanisms from MS^3^ spectra as well as from MS^2^ analyses of deuterated analogs^45^. An overview of fragment ion annotations is given in Fig. 9 along with annotations from libraries and from our spectroscopic investigation. Spectral overlays that form the basis for our annotations are presented in Supplementary Figs. S25–S33.

A quick glance at Fig. 9 suggests that the library annotations are again incorrect. In general, the large majority of fragment ion structures established by IRIS are cyclized species, versus nearly exclusively linear structures in the databases. An exception is the *m/z* 112 ion in mzCloud, which is correctly annotated, although different MS^n^ pathways lead to this *m/z* value, and the spectroscopic results show that they do not all correspond to the same molecular structure. The manual annotations of Zhang et al.^44^ include many cyclic product ion structures that generally match closely with our results. Nonetheless, some deviations in assigned structures are also noted. Especially, cyclization upon nucleophilic attack leads mostly to 5- or 6-membered rings according to our spectroscopic analyses, whereas several 8-membered rings are proposed in Zhang et al.^44^.

## Conclusions

**Fig. 10:**
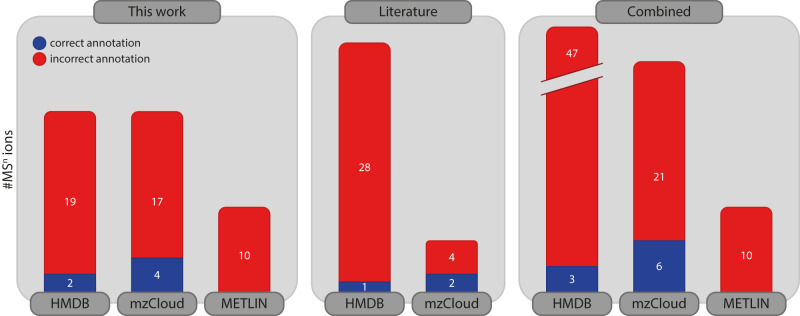
Number of correct and incorrect MS/MS structure annotations. Summary of correct/incorrect MS^n^ structure annotations as verified by IR ion spectroscopy in this work; the large majority of annotations in major MS/MS libraries are likely incorrect. Additional spectroscopically established CID product ion structures collected from literature confirm this trend (see Supplementary Figs. S67–S89).

We conclude that the library annotations rarely agree with the fragment ion structures established spectroscopically in this work. Based on the 36 spectroscopically established CID product ion structures in this work, the fractions of correct and incorrect annotations are displayed in the diagram in Fig. 10, where it should be noted that not all 36 ions are in each database. We consider a database entry to be correct when the molecular structure matches, ignoring discrepancies in protonation site. mzCloud appears to slightly outperform HMDB and METLIN, although the fraction of correct annotations is too low to be reliably used.

Admittedly, our test set is extremely small and clearly too limited to derive statistically significant percentages of correct and incorrect annotations. Nonetheless, this set of 36 spectroscopically established CID product ion structures is probably the largest published thus far. Furthermore, 45 additional spectroscopically established CID product ion structures were collected from literature; see Section 7 in the Supplementary Information. This dataset contains mostly peptide sequence ions and was also contrasted against structural annotations in the MS/MS libraries, which confirms the trend: only 1 out of 29 HMDB entries is correct and 2 out of 6 mzCloud entries are correct (see Fig. 10). Overall, this test reveals the generic deficiency of library annotations: many of the product ion structures identified here possess cyclic chemical moieties that are formed by rearrangements in the CID reaction. The in silico algorithms of MetFrag and CFM-ID do not consider such rearrangements.

Small-molecule neutral losses (e.g., H_2_O, NH_3_) are common in ESI-MS/MS and are also represented in our small test set. They are of particular interest since they often occur already at low CID activation energies and, hence early on in the MS^n^ spectral tree. In the in silico algorithms, these neutral losses are often treated separately and are implemented early on in the fragmentation tree^11^. We observe in our IR spectroscopic analysis that many H_2_O and NH_3_ loss events induce cyclization of the ionic fragment. A significant improvement may be achieved by devising new algorithms that correctly annotate the products of these small-molecule neutral losses, which would better model the entire downstream fragmentation tree and, hence, the predicted MS/MS fragment masses.

The current machine-learning revolution is having a strong impact on the development of in silico MS/MS algorithms. The performance of fragmentation tools currently available is benchmarked in numerous reports in the MS literature, see, for instance, the CASMI contest^46,47^. The performance is usually evaluated based on the ability to correctly identify molecular (sub)structures from an experimental MS/MS spectrum, without other a priori knowledge of the query compound. In the most recent competition, although the elemental composition was often correctly identified from the mass-spectral data, the correct 2D chemical structure was retrieved from the MS/MS spectrum in only about 10% of cases^48^. A benchmark on the ability to correctly annotate MS/MS fragment ions may serve as an alternative test that could provide deeper insight into the underlying fundamentals of why one algorithm performs better than another one.

## Methods

### Chemicals and sample preparation

Homocitrulline, citrulline, arginine, and α-amino-adipic acid were obtained from MetaSci (Toronto, Canada). Urocanic acid and reference compounds were acquired from Sigma-Aldrich (St. Louis, USA). Stock solutions were prepared by dissolving the samples in 50:50 MeOH-H_2_O (LC-MS grade, obtained from Sigma-Aldrich (St. Louis, USA)). Approximately 0.5% of formic acid (LC-MS grade, obtained from Sigma-Aldrich (St. Louis, USA)) was added to promote protonation.

### Experimental

A modified 3D quadrupole ion trap mass spectrometer (Bruker, AmaZon Speed ETD) is used to perform IRIS experiments. The instrument was modified to enable optical access to the trapped ions^49^. Solutions were diluted to approximately 1 μM and introduced into the MS by direct infusion at 120–180 μl/h flow rates through an electrospray ionization (ESI) source. Typical operating parameters were capillary and end plate voltages of 4500 V and 500 V, respectively, and a nitrogen dry gas temperature of 180 °C flowing at 4 L min^−1^. MS/MS and MS^n^ fragments were generated by collision-induced dissociation (CID) of the mass-isolated protonated precursor ion. The CID amplitude was optimized to maximize the signal on the fragment mass peak of interest. Additionally, a Fourier transform ion cyclotron resonance (FTICR-MS, Bruker, SolariX XR 7 T) mass spectrometer equipped with an identical ESI source was used to determine the accurate mass and hence the molecular formula of all MS^n^ fragments (see Supplementary Figs. S62–S66 and Supplementary Tables S1–S5).

In the ion trap, the MS^n^ fragment of interest is mass-isolated and subjected to IR analysis using the FELIX free-electron laser. The FELIX IR laser was set to produce IR radiation between 560 and 1950 cm^−1^ in the form of 5–10 μs long macropulses of 20–160 mJ at a repetition rate of 5 or 10 Hz and with a bandwidth of ~0.4% of the center frequency. An optical parametric oscillator (LaserSpec, Belgium)^50^ was also used to obtain IR spectra in the 3250–3800 cm^−1^ spectral range for some MS/MS fragments of arginine and citrulline. The OPO produces 5.6 nJ pulses of 35 ps duration at a 80-MHz repetition rate and with a bandwidth of 0.5 cm^−1^.

When the laser frequency is in resonance with a vibrational transition, multiple-photon absorption by the ions results in an increase of their internal energy and, eventually, in their fragmentation. The extent of fragmentation in the mass spectrum can be monitored as a function of the IR laser frequency. Hence, an IR vibrational spectrum can be reconstructed from a series of mass spectra by monitoring the IR-induced dissociation yield, defined here as ${{{{\mathrm{ln}}}}}\left[\sum {I}_{{all\; ions}}/{I}_{{precursor\; ion}}\right]$, as function of IR frequency^51^. The yield is obtained from 6 averaged mass spectra at each laser frequency, advancing in steps of 5 cm^−1^. The frequency of the laser was calibrated using a grating spectrometer and the yield was linearly corrected for variations in the laser pulse energy. For displaying purposes, each experimental spectrum is normalized to the most intense peak.

### Computational

Theoretical spectra were generated for possible fragment ion structures using density functional theory (DFT) at the B3LYP/6-31++G(d,p) level. As input structures, we used annotations suggested in the HMDB, mzCloud, and METLIN databases, as well as many alternative structures based on chemical intuition. In our computational workflow, the SMILES 2D-structure format was used as input for the cheminformatics toolbox RDKit^52^ that generates all possible protonation isomers as well as stereoisomers. Structures containing only one stereocenter were randomly assigned to be R or S, as enantiomers have the same IR spectrum. For each protonation isomer, a conformational search was performed using a distance geometry algorithm to find 500 random 3D-conformations, after which the structures were optimized with a classical force field^53^ and clustered based on similarity. A maximum of 20 unique structures were then selected based on root-mean-square deviations of atomic positions, and these were submitted to Gaussian16^54^ for geometry optimizations and frequency calculations at the semi-empirical PM6 level. Optimizations that converge to one of the other isomers, unconverged calculations and structures with broken bonds are automatically removed. In addition, conformations were filtered by a relative energy cut-off of 40 kJ mol^−1^. The remaining structures were then re-optimized at the B3LYP/6-31++G(d,p) level of theory^55,56^ and harmonic vibrational frequencies were calculated and scaled by a factor of 0.975 to correct for anharmonicity^57^. A factor of 0.955 was applied for the H-stretching frequencies in the 3-μm wavelength range^57^. A convolution of the stick spectra with a Gaussian line shape of 20 cm^−1^ full width at half maximum produced the theoretical spectra that were compared with the experimental spectra. The B3LYP-optimized geometries were also employed for a single-point electronic energy calculation at the MP2/6-31++G(d,p) level of theory to improve relative energies of isomeric structures.

### Supplementary information

Supplementary Information

## Data Availability

In the Supplementary Information file, we provide comparisons of experimental versus theoretical spectra underlying our proposed structural annotations in Figs. 3–5, 7, and 9. Furthermore, we provide all DFT-optimized geometries of fragment ion structures considered. Plausible reaction pathways connecting precursor ion and CID-fragment ion structure established through IRIS are suggested. Recorded high-resolution MS/MS spectra and accurate mass values of CID fragments studied are listed. We provide a list of Splash Keys identifying the accessed MS/MS spectra in the HMDB. Finally, additional spectroscopically identified CID-fragment ion structures from various precursors taken from the literature (mainly small peptides) are presented. The experimental IRIS and MS/MS data underlying the figures in the main text and in the Supplementary Information is available through the Radboud Data Repository (10.34973/e77p-ae39).
