# Supplementary material for: A spectroscopic test suggests that fragment ion structure annotations in MS/MS libraries are frequently incorrect
Source: Commun Chem. 2024 Feb 14;7:30. doi: 10.1038/s42004-024-01112-7 (PMC10867025; doi:10.1038/s42004-024-01112-7)
Supplement: Supplementary file 1 — Supplementary Information [file 42004_2024_1112_MOESM1_ESM.pdf]

Supplementary Information

A spectroscopic test suggests that fragment ion structure annotations in MS/MS libraries are frequently incorrect

Lara van Tetering[1], Sylvia Spies[1], Quirine D.K. Wildeman[1], Kas J. Houthuijs[1], Rianne E. van Outersterp[1], Jonathan Martens[1], Ron A. Wevers[2], David S. Wishart[3], Giel Berden[1], and Jos Oomens[1, 4]

[1] *Radboud University, Institute for Molecules and Materials, FELIX Laboratory, Toernooiveld 7, 6525ED Nijmegen, The Netherlands*
[2] *Department of Laboratory Medicine, Translational Metabolic Laboratory, Radboud University Medical Center, Geert Grooteplein Zuid 10, 6525GA Nijmegen, The Netherlands*
[3] *Departments of Computing Science and Biological Sciences, University of Alberta, Edmonton, AB, Canada*
[4] *van 't Hoff Institute for Molecular Sciences, University of Amsterdam, Science Park 904, 1098XH Amsterdam, The Netherlands*

Contents

1 Molecular formula of MS^n fragments **S4**

2 Infrared spectra **S5**

2.1 α-Amino-adipic acid S5
2.1.1 Precursor ion, m/z 162 . . . . . S5
2.1.2 MS^2 fragment ion, m/z 144 . . . . . S6
2.1.3 MS^2 fragment ion, m/z 116 . . . . . S7
2.1.4 MS^2 fragment ion, m/z 98 . . . . . S8
2.2 Urocanic acid S8
2.2.1 Precursor ion, m/z 139 . . . . . S8
2.2.2 MS^2 fragment ion, m/z 121 . . . . . S9
2.3 Citrulline S10
2.3.1 Precursor ion, m/z 176 . . . . . S10
2.3.2 MS^2 fragment ion, m/z 159 . . . . . S11
2.3.3 MS^2 fragment ion, m/z 158 . . . . . S12
2.3.4 MS^3 fragment ion, m/z 141 (via fragment ion m/z 159) S12
2.3.5 MS^2 fragment ion, m/z 116 . . . . . S13
2.3.6 MS^3 fragment ion, m/z 115 (via fragment ion m/z 159) S15
2.3.7 MS^3 fragment ion, m/z 115 (via fragment ion m/z 158) S16
2.3.8 MS^3 fragment ion, m/z 113 (via fragment ion m/z 159) S17
2.4 Homocitrulline S18
2.4.1 Precursor ion, m/z 190 . . . . . S18
2.4.2 MS^2 fragment ion, m/z 173 . . . . . S19
2.4.3 MS^2 fragment ion, m/z 147 . . . . . S20
2.4.4 MS^2 fragment ion, m/z 144 . . . . . S20
2.4.5 MS^2 and MS^3 fragment ion, m/z 130 (via fragment ion m/z 147) S21
2.4.6 MS^2 and MS^3 fragment ion, m/z 127 (via fragment ion m/z 144) S22
2.4.7 MS^3 fragment ion, m/z 127 (via fragment ion m/z 173) S23
2.4.8 MS^n fragment ion, m/z 84 . . . . . S24
2.4.9 MS^4 fragment ion, m/z 84 (via MS^3 fragment ion m/z 190 → 173 → 127) S25
2.5 Arginine S25
2.5.1 Precursor ion, m/z 175 . . . . . S25
2.5.2 MS^2 fragment ion, m/z 158 . . . . . S26
2.5.3 MS^2 fragment ion, m/z 157 . . . . . S26
2.5.4 MS^3 fragment ion, m/z 140 (via fragment ion m/z 158 and fragment ion m/z 157) S27
2.5.5 MS^2 fragment ion, m/z 130 . . . . . S27
2.5.6 MS^2 and MS^3 fragment ion, m/z 116 (via fragment ion m/z 158) S28
2.5.7 MS^3 fragment ion, m/z 115 (via fragment ion m/z 157) S29
2.5.8 MS^n fragment ion, m/z 112 . . . . . S30
2.6 Supplementary Note 1: Comparison of MS^2 fragment ion m/z 116 of arginine and citrulline S32
2.7 Supplementary Note 2: Analysis of arginine MS^n fragment ion at m/z 112 . . . . . S32

3 Optimized geometries of fragment ion structures **S34**

3.1 α-Amino-adipic acid S34
3.1.1 Precursor ion, m/z 162 . . . . . S34
3.1.2 MS^2 fragment ion, m/z 144 . . . . . S34
3.1.3 MS^2 fragment ion, m/z 116 . . . . . S35
3.1.4 MS^2 fragment ion, m/z 98 . . . . . S36
3.2 Urocanic acid S37
3.2.1 Precursor ion, m/z 139 . . . . . S37
3.2.2 MS^2 fragment ion, m/z 121 . . . . . S37
3.3 Citrulline S38
3.3.1 Precursor ion, m/z 176 . . . . . S38
3.3.2 MS^2 fragment ion, m/z 159 . . . . . S38

3.3.3 MS^2 fragment ion, m/z 158 . . . S40
3.3.4 MS^3 fragment ion, m/z 141 (via fragment ion m/z 159) . . . S40
3.3.5 MS^2 fragment ion, m/z 116 . . . S40
3.3.6 MS^3 fragment ion, m/z 115 (via fragment ion m/z 159) . . . S42
3.3.7 MS^3 fragment ion, m/z 115 (via fragment ion m/z 158) . . . S43
3.3.8 MS^3 fragment ion, m/z 113 (via fragment ion m/z 159) . . . S44
3.4 Homocitrulline . . . S45
3.4.1 Precursor ion, m/z 190 . . . S45
3.4.2 MS^2 fragment ion, m/z 173 . . . S45
3.4.3 MS^2 fragment ion, m/z 147 . . . S46
3.4.4 MS^2 fragment ion, m/z 144 . . . S46
3.4.5 MS^2 and MS^3 fragment ion, m/z 130 (via fragment ion m/z 147) . . . S47
3.4.6 MS^2 and MS^3 fragment ion, m/z 127 (via fragment ion m/z 144) . . . S47
3.4.7 MS^3 fragment ion, m/z 127 (via fragment ion m/z 173) . . . S48
3.4.8 MS^n fragment ion, m/z 84 . . . S49
3.4.9 MS^4 fragment ion, m/z 84 (via MS^3 fragment ion m/z 190 → 173 → 127) . . . S49
3.5 Arginine . . . S50
3.5.1 Precursor ion, m/z 175 . . . S50
3.5.2 MS^2 fragment ion, m/z 158 . . . S50
3.5.3 MS^2 fragment ion, m/z 157 . . . S51
3.5.4 MS^3 fragment ion, m/z 140 (via fragment ion m/z 158 and fragment ion m/z 157) S51
3.5.5 MS^2 fragment ion, m/z 130 . . . S52
3.5.6 MS^2 and MS^3 fragment ion, m/z 116 (via fragment ion m/z 158) . . . S52
3.5.7 MS^3 fragment ion, m/z 115 (via fragment ion m/z 157) . . . S53
3.5.8 MS^n fragment ion, m/z 112 . . . S53

4 Proposed fragmentation mechanisms S55
4.1 α-Amino-adipic acid . . . S55
4.1.1 m/z 162 → 144 . . . S55
4.1.2 m/z 162 → 116 . . . S55
4.1.3 m/z 162 → 98 . . . S55
4.2 Urocanic acid . . . S55
4.2.1 m/z 139 → 121 . . . S55
4.3 Citrulline . . . S56
4.3.1 m/z 176 → 159 . . . S56
4.3.2 m/z 176 → 158 . . . S56
4.3.3 m/z 176 → 116 . . . S56
4.3.4 m/z 176 → 159 → 115 . . . S56
4.3.5 m/z 176 → 158 → 115 . . . S57
4.3.6 m/z 176 → 159 → 113 . . . S57
4.4 Homocitrulline . . . S57
4.4.1 m/z 190 → 173 . . . S57
4.4.2 m/z 190 → 147 . . . S57
4.4.3 m/z 190 → 144 . . . S57
4.4.4 m/z 190 → 147 → 130 & m/z 190 → 130 (via fragment m/z 147) . . . S58
4.4.5 m/z 190 → 144 → 127 & m/z 190 → 127 (via fragment m/z 144) . . . S58
4.4.6 m/z 190 → 173 → 127 . . . S58
4.4.7 m/z 190 → 173 → 127 → 84 . . . S58
4.4.8 m/z 190 → 173 → 84 . . . S58
4.4.9 m/z 190 → 144 → 127 → 84 & m/z 190 → 84 (via fragment m/z 127) . . . S59
4.5 Arginine . . . S59
4.5.1 m/z 175 → 158 . . . S59
4.5.2 m/z 175 → 157 . . . S59
4.5.3 m/z 175 → 130 . . . S59
4.5.4 m/z 175 → 116 . . . S60
4.5.5 m/z 175 → 158 → 116 . . . S60

4.5.6 m/z 175 → 157 → 115 . . . . . S60
4.5.7 m/z 175 → 158 → 112 and m/z 175 → 112 (via fragment m/z 158) S60
4.5.8 m/z 175 → 157 → 112 . . . . . S61

5 Recorded MS/MS Spectra **S62**

6 Accessed HMDB Spectra **S64**

7 Additional molecules **S65**
7.1 2-hydroxynicotinic acid S65
7.2 Alanylasparagine (AlaAsn) S65
7.3 Alanylglutamine (AlaGln) S66
7.4 Alanylserine (AlaSer) S66
7.5 Alanylthreonine (AlaThr) S67
7.6 Arginylglycine (ArgGly) S67
7.7 Asparagine S67
7.8 Asparaginyl-alanine (AsnAla) S68
7.9 Asparaginyl-serine (AsnSer) S69
7.10 Asparaginyl-threonine (AsnThr) S69
7.11 Asparaginyl-valine (AsnVal) S70
7.12 Aspartic acid S70
7.13 Glutamic acid S71
7.14 Glutamine S71
7.15 Glutaminylalanine (GlnAla) S72
7.16 Glycyl-arginine (GlyArg) S72
7.17 Glycyl-serine (GlySer) S73
7.18 Glycyl-threonine (GlyThr) S73
7.19 Leu-enkephelin S73
7.20 Prolyl-serine (ProSer) S74
7.21 Prolyl-threonine (ProThr) S74
7.22 Testosterone S74
7.23 Triglycine S75

8 Supplementary References **S76**

1 Molecular formula of MS^n fragments

High-resolution MS/MS spectra were obtained on a Bruker SolariX FTICR-MS (see Figures S62-S66 in Section 5) to establish the accurate mass and to derive the elemental formula of all CID product ions investigated.

Table S1: α-Amino-adipic acid

m/z value	Measured m/z	Molecular formula
162	162.0760	$C_6H_{12}NO_4^+$
144	144.0655	$C_6H_{10}NO_3^+$
116	116.0706	$C_5H_{10}NO_2^+$
98	98.0600	$C_5H_8NO^+$

Table S2: Urocanic acid

m/z value	Measured m/z	Molecular formula
139	139.0502	$C_6H_7N_2O_2^+$
121	121.0396	$C_6H_5N_2O^+$

Table S3: Citrulline

m/z value	Measured m/z	Molecular formula
176	176.1030	$C_6H_{14}N_3O_3^+$
159	159.0764	$C_6H_{11}N_2O_3^+$
158	158.0923	$C_6H_{12}N_3O_2^+$
141	141.0658	$C_6H_9N_2O_2^+$
116	116.0705	$C_5H_{10}NO_2^+$
115	115.0865	$C_5H_{11}N_2O^+$
113	113.0709	$C_5H_9N_2O^+$

Table S4: Homocitrulline

m/z value	Measured m/z	Molecular formula
190	190.1185	$C_7H_{16}N_3O_3^+$
173	173.0920	$C_7H_{13}N_2O_3^+$
147	147.1127	$C_6H_{15}N_2O_2^+$
144	144.1131	$C_6H_{14}N_3O^+$
130	130.0862	$C_6H_{12}NO_2^+$
127	127.0865	$C_6H_{11}N_2O^+$
84	84.0807	$C_5H_{10}N^+$

Table S5: Arginine

m/z value	Measured m/z	Molecular formula
175	175.1190	$C_6H_{15}N_4O_2^+$
158	158.0924	$C_6H_{12}N_3O_2^+$
157	157.1084	$C_6H_{13}N_4O^+$
140	140.0818	$C_6H_{10}N_3O^+$
130	130.0974	$C_5H_{12}N_3O^+$
116	116.0705	$C_5H_{10}NO_2^+$
115	115.0865	$C_5H_{11}N_2O^+$
112	112.0868	$C_5H_{10}N_3^+$

2 Infrared spectra

Relative energies as well as 2D-structures are inlayed in each panel. For the spectroscopically established structure in this work, the DFT optimized structure is also shown. In cases where two conformers contribute to the measured spectrum, the energies are given for both DFT optimized structures.

Input 2D-structures from the databases may optimize to another isomeric structure. If this is the case for the lowest-energy conformer, the predicted spectrum is not shown here. Furthermore, there might be more than one protonation site shown for some structures as not all databases include protonation sites.

2.1 α-Amino-adipic acid

2.1.1 Precursor ion, m/z 162

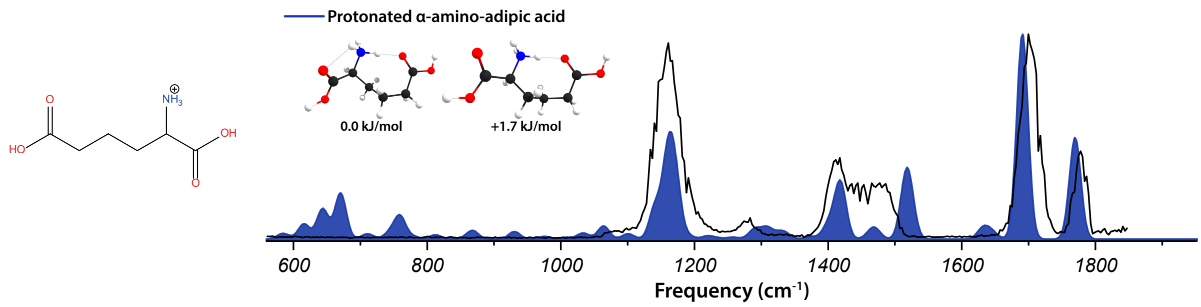

Figure S1: IRIS spectrum of protonated α-amino-adipic acid compared with predicted IR spectrum of lowest-energy protonation site.

2.1.2 MS^2 fragment ion, m/z 144

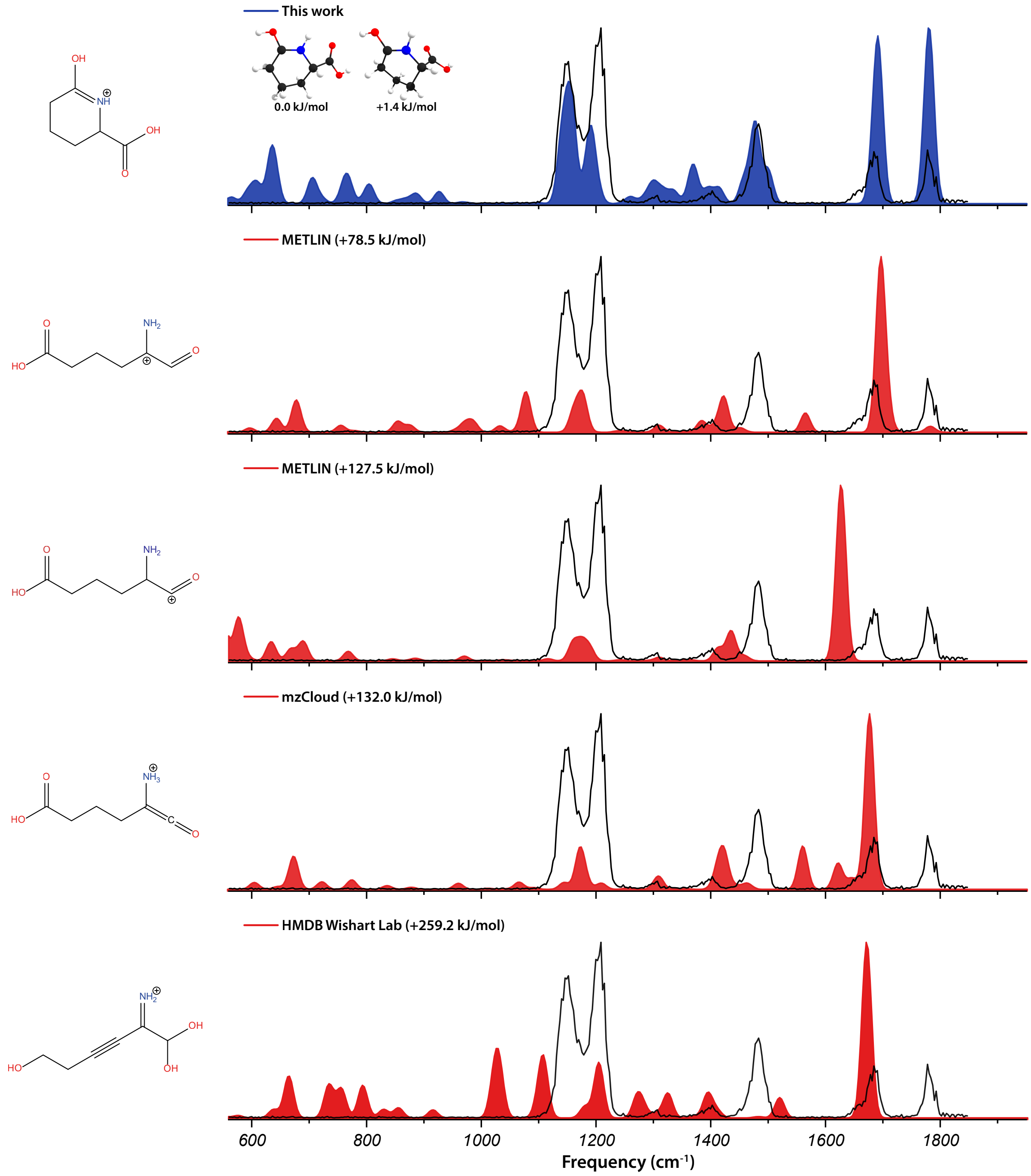

Figure S2: IRIS spectrum of the *m/z* 144 fragment ion produced directly from α-amino-adipic acid in comparison to predicted IR spectra of structures found in this work, the HMDB, METLIN and mzCloud.

2.1.3 MS^2 fragment ion, m/z 116

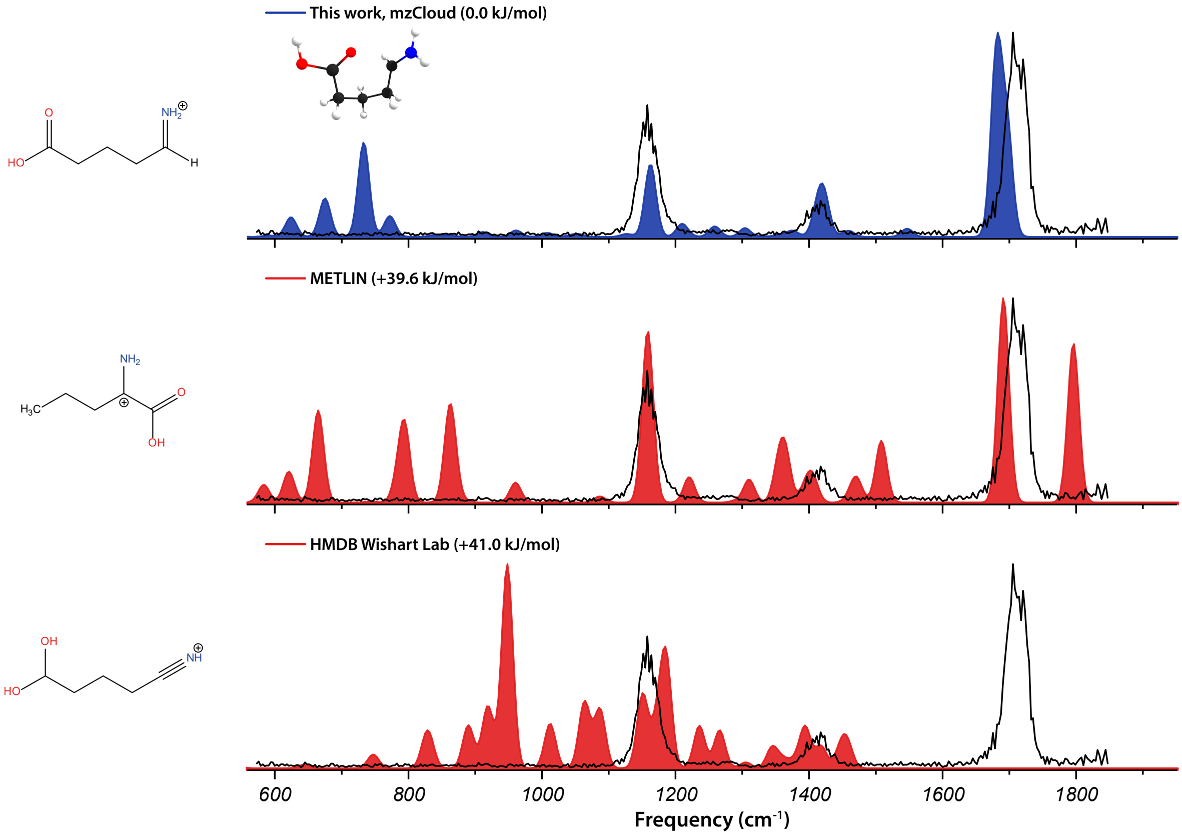

Figure S3: IRIS spectrum of the m/z 116 fragment ion produced directly from α-amino-adipic acid in comparison to predicted IR spectra of structures found in this work, the HMDB, METLIN and mzCloud. The structure proposed in mzCloud is identical to the spectroscopically established structure.

2.1.4 MS^2 fragment ion, m/z 98

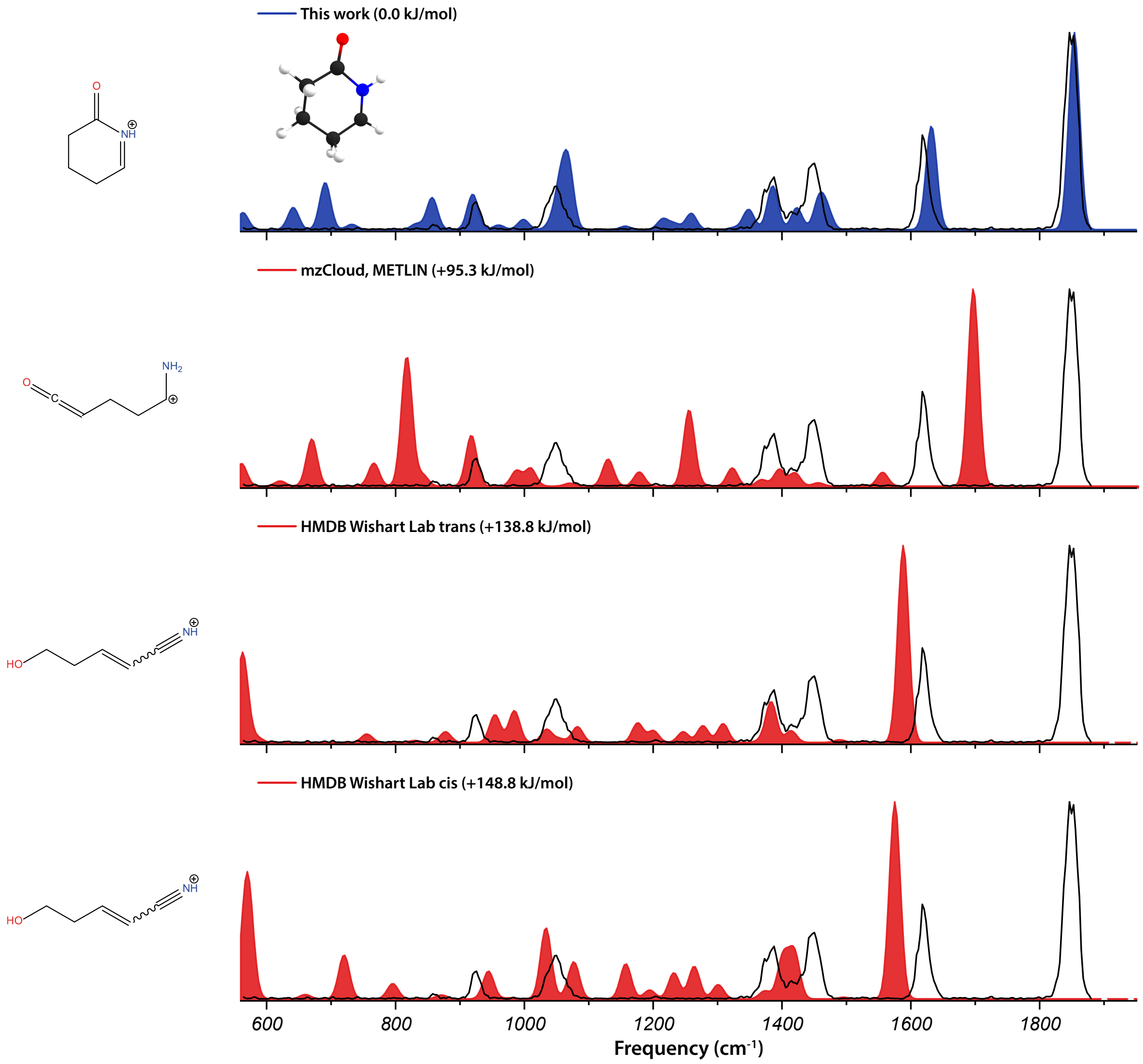

Figure S4: IRIS spectrum of the *m/z* 98 fragment ion produced directly from α-amino-adipic acid in comparison to predicted IR spectra of structures found in this work, the HMDB, METLIN and mzCloud.

2.2 Urocanic acid

2.2.1 Precursor ion, m/z 139

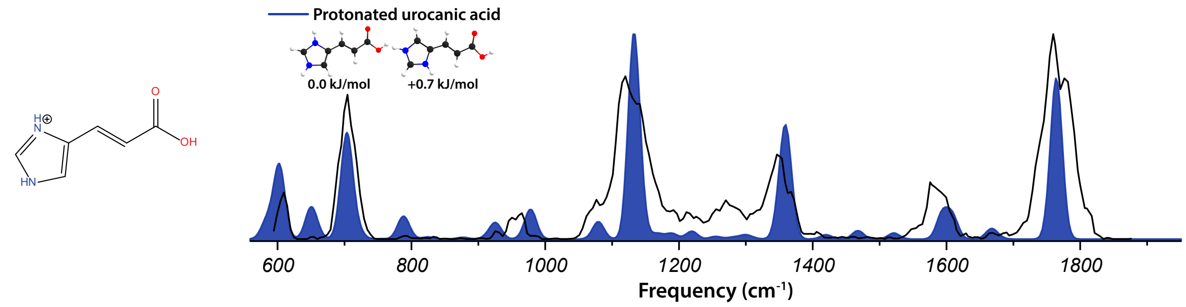

Figure S5: IRIS spectrum of protonated urocanic acid compared with predicted IR spectrum of lowest-energy protonation site.

2.2.2 MS^2 fragment ion, m/z 121

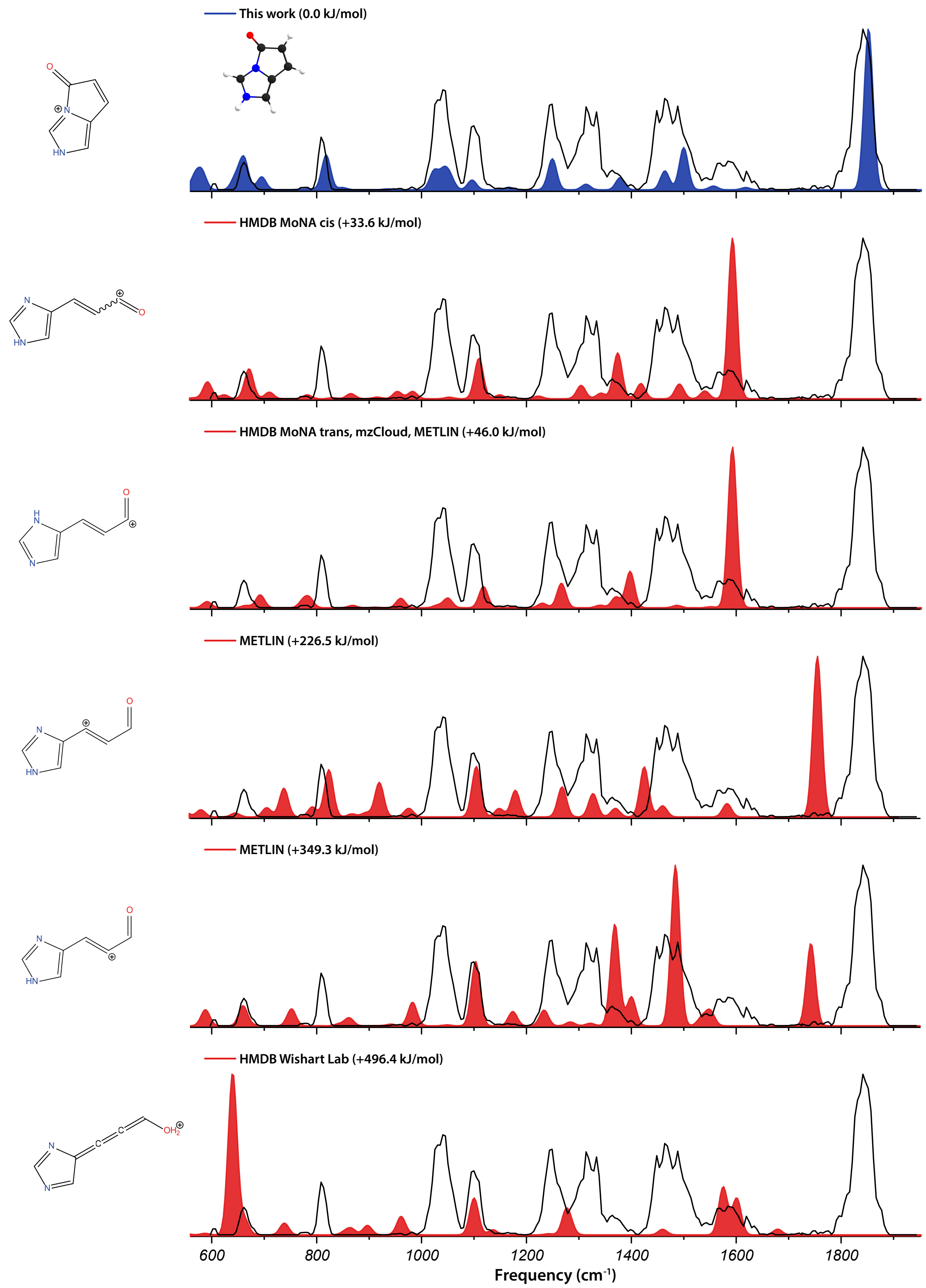

Figure S6: IRIS spectrum of the *m/z* 121 fragment ion produced directly from urocanic acid in comparison to predicted IR spectra of structures found in this work, the HMDB, METLIN and mzCloud.

2.3 Citrulline

2.3.1 Precursor ion, m/z 176

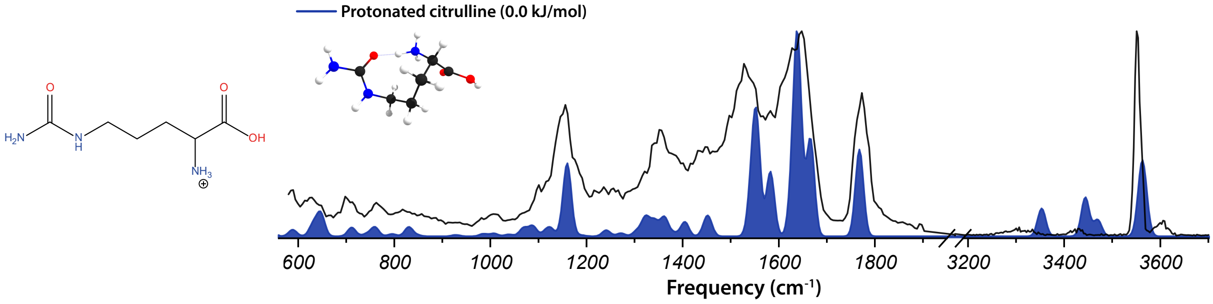

Figure S7: IRIS spectrum of protonated citrulline compared with predicted IR spectrum of lowest-energy protonation site.

2.3.2 MS^2 fragment ion, m/z 159

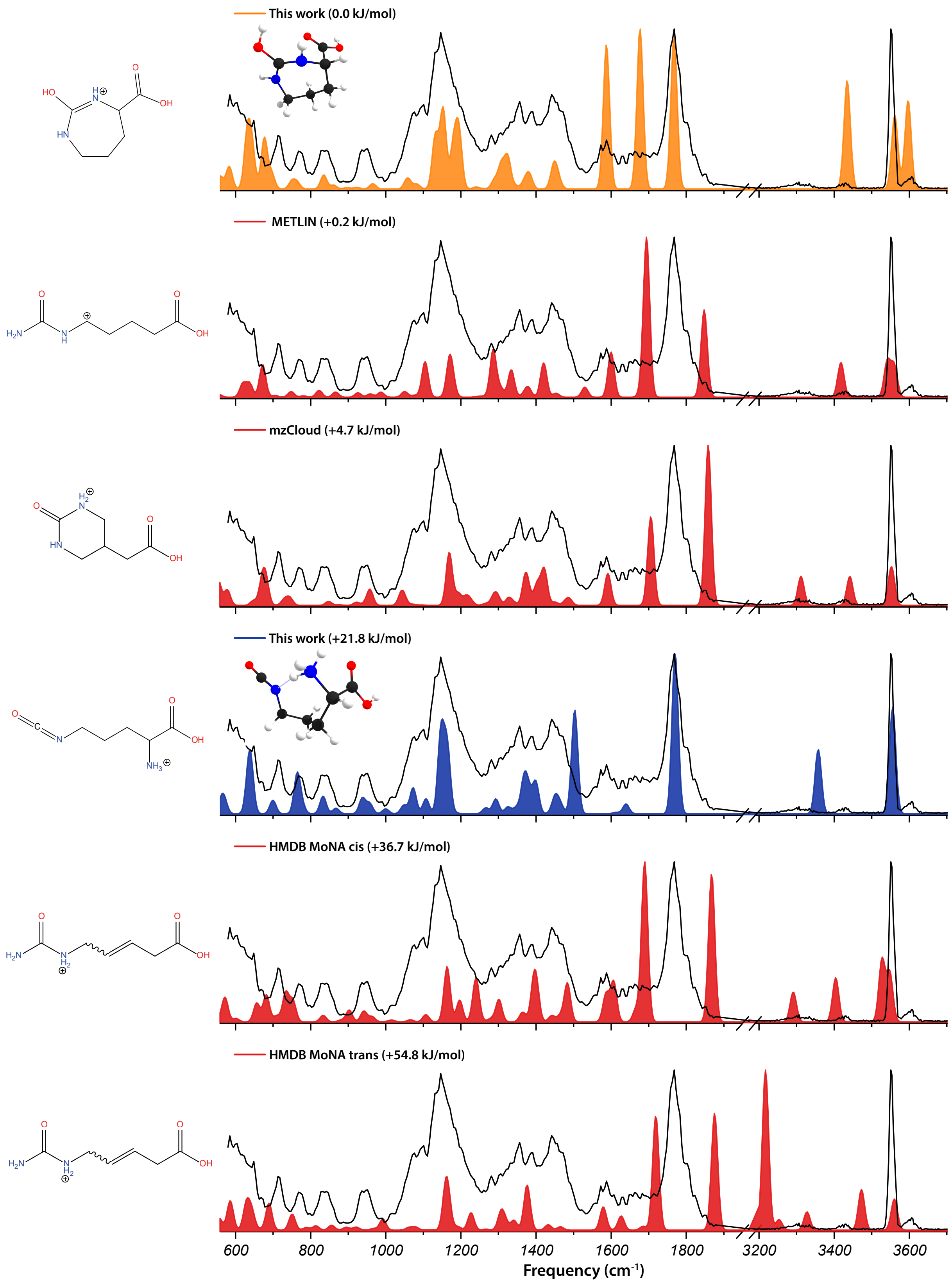

Figure S8: IRIS spectrum of the *m/z* 159 fragment ion produced directly from citrulline in comparison to predicted IR spectra of structures found in this work, the HMDB, METLIN and mzCloud.

2.3.3 MS^2 fragment ion, m/z 158

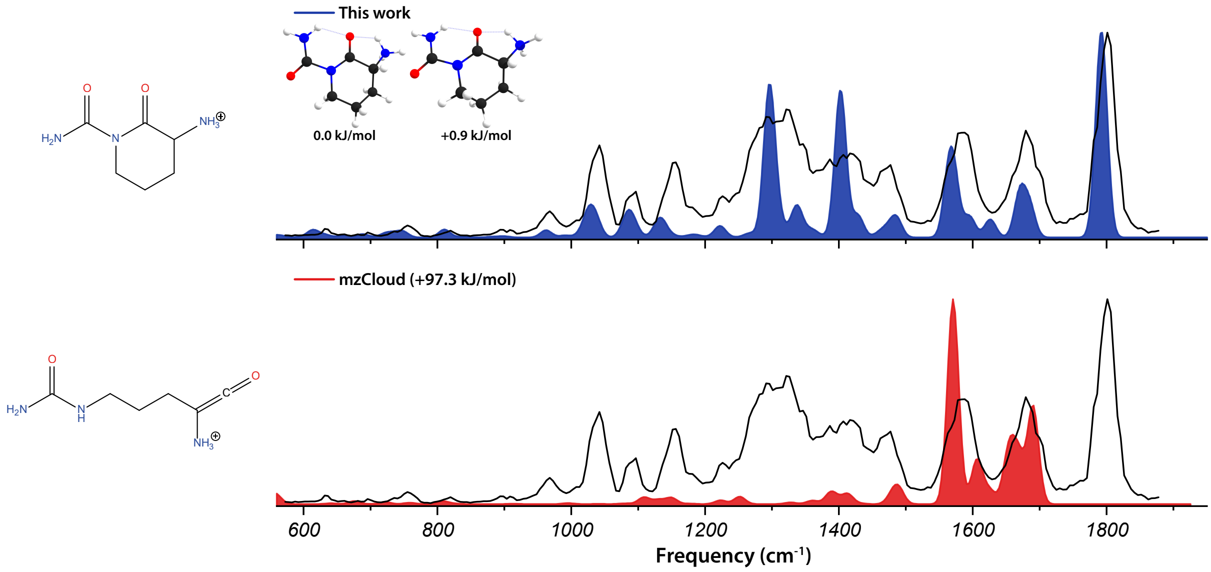

Figure S9: IRIS spectrum of the *m/z* 158 fragment ion produced directly from citrulline in comparison to predicted IR spectra of structures found in this work and mzCloud.

2.3.4 MS^3 fragment ion, m/z 141 (via fragment ion m/z 159)

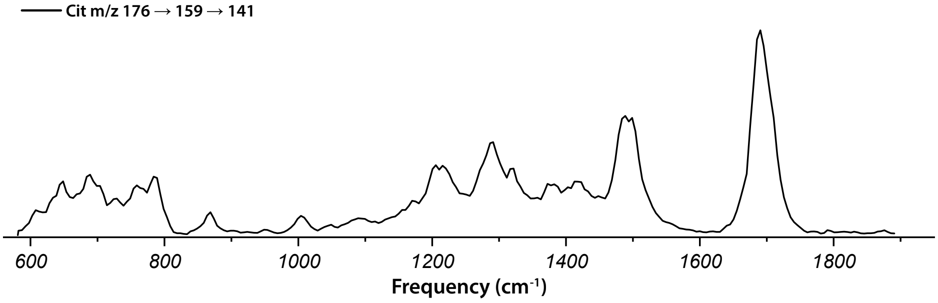

Figure S10: IRIS spectrum of the *m/z* 141 fragment ion produced via the *m/z* 159 intermediate in an MS^3 experiment. No matching structures found.

2.3.5 MS^2 fragment ion, m/z 116

This fragment ion forms two structures, named structure I and structure II, as elaborated in Section 2.6.

MS^2 fragment ion, m/z 116 (structure I)

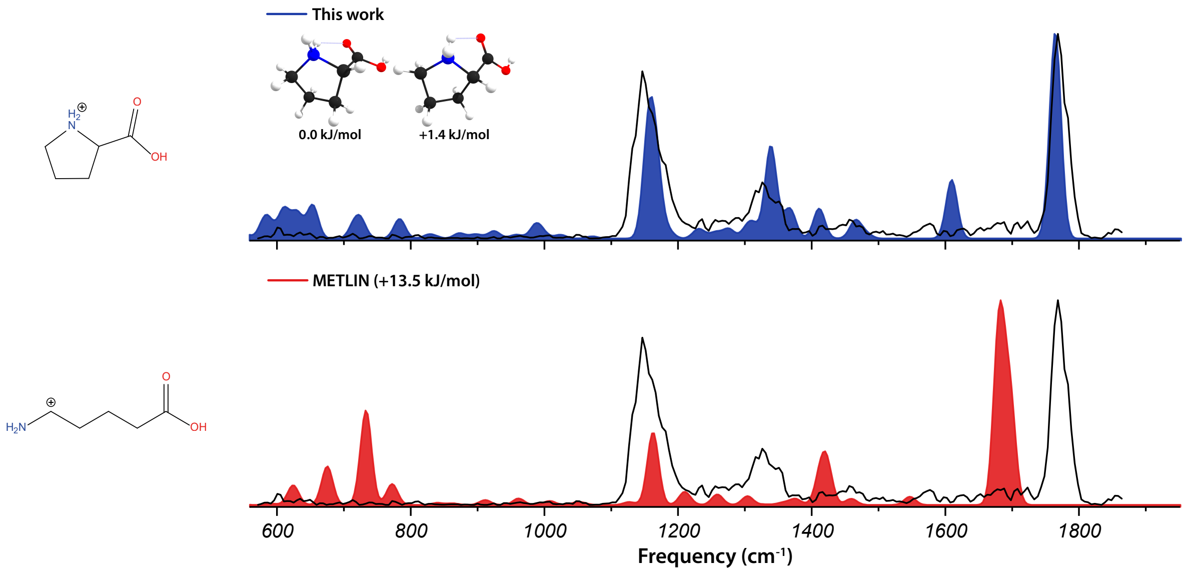

Figure S11: IRIS spectrum of the MS^2 fragment ion of *m/z* 116 directly formed from citrulline, measured in the IR induced dissociation channel in *m/z* 70. The spectrum is compared with predicted IR spectra of structures found in this work and METLIN.

MS^2 fragment ion, m/z 116 (structure II)

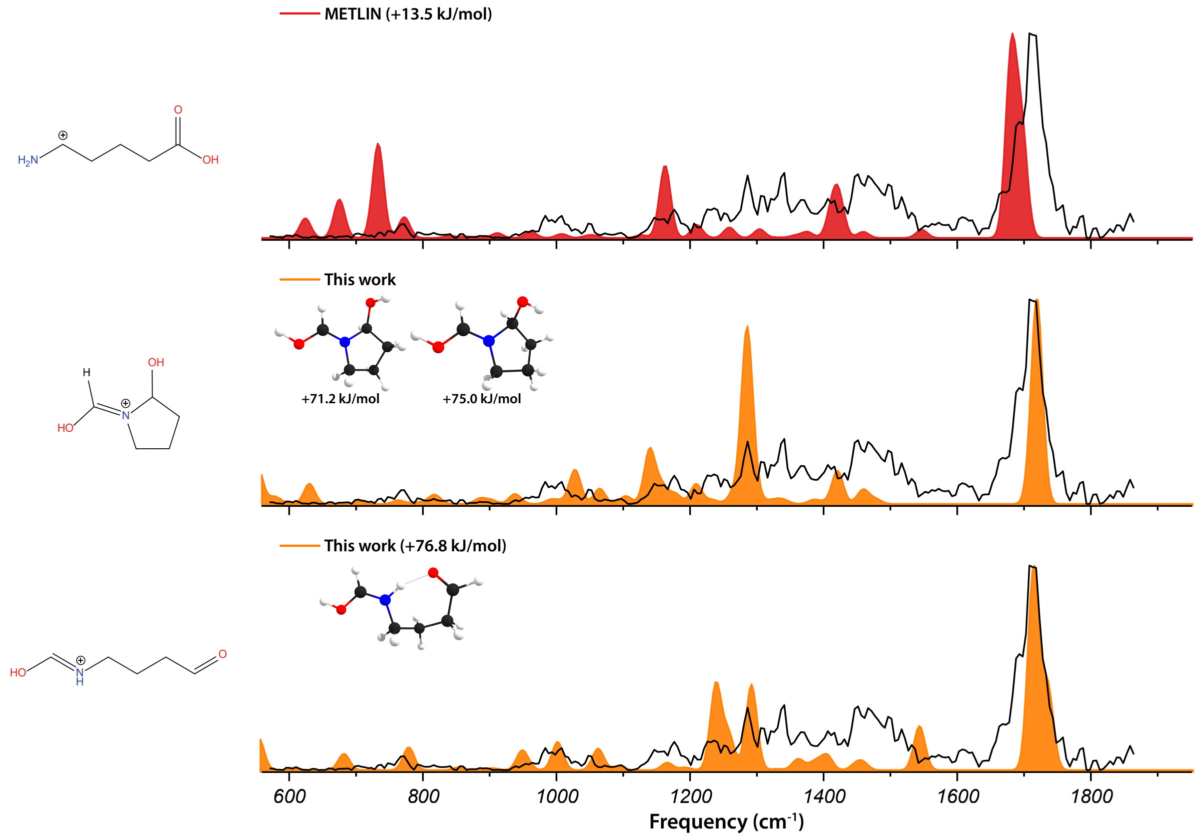

Figure S12: IRIS spectrum of the MS^2 fragment ion of m/z 116 directly formed from citrulline, measured in the IR induced dissociation channel in m/z 98 and 71. The spectrum is compared with predicted IR spectra of structures found in this work and METLIN. Energies are relative to global minimum structure of Figure S11.

2.3.6 MS^3 fragment ion, m/z 115 (via fragment ion m/z 159)

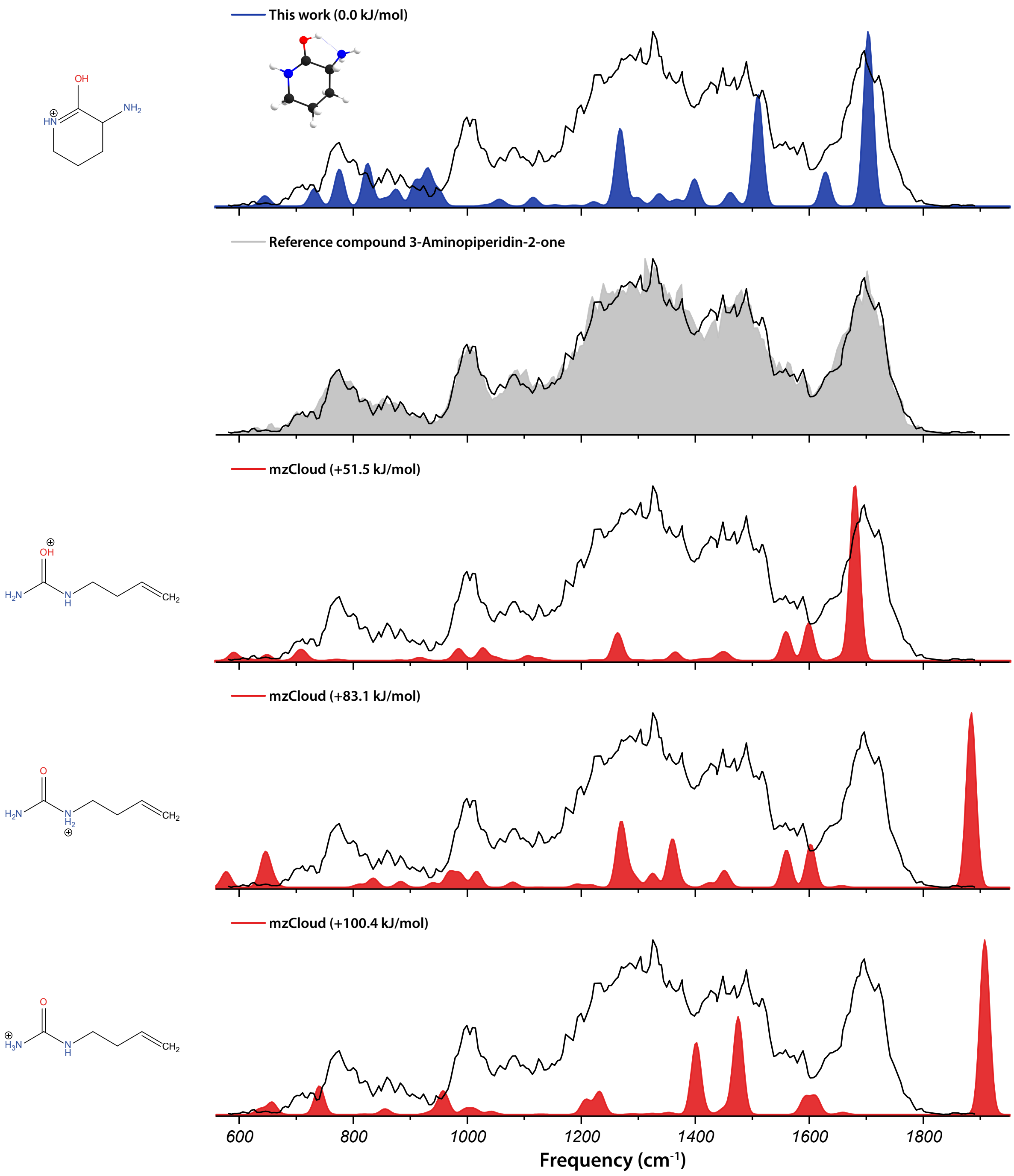

Figure S13: IRIS spectrum of the *m/z* 115 fragment ion produced via the *m/z* 159 intermediate in an MS^3 experiment in comparison to predicted IR spectra of structures found in this work and mzCloud. Experimental spectrum of reference compound 3-Aminopiperidin-2-one shown for comparison.

2.3.7 MS^3 fragment ion, m/z 115 (via fragment ion m/z 158)

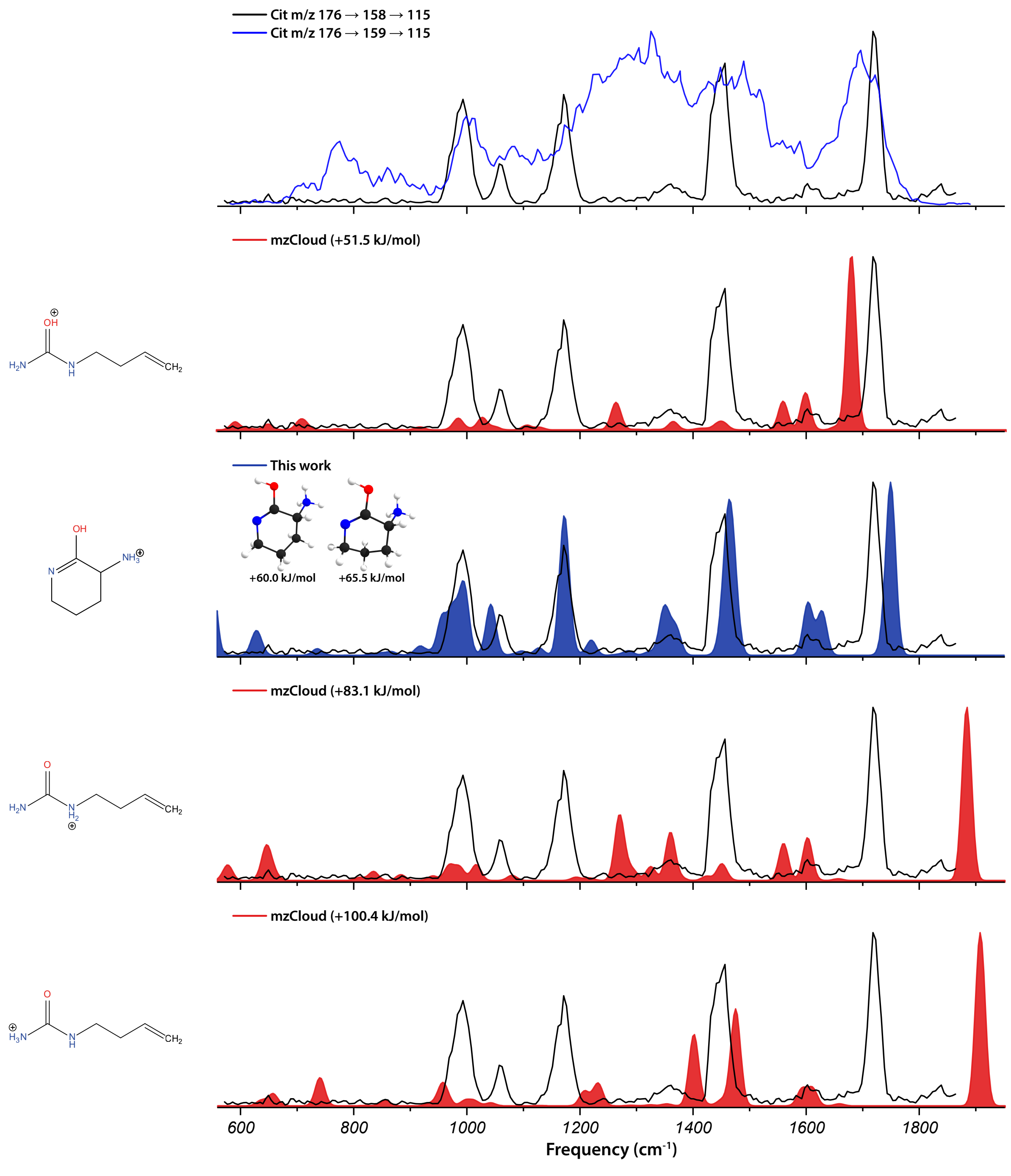

Figure S14: IRIS spectrum of the *m/z* 115 fragment ion produced via the *m/z* 158 intermediate in an MS^3 experiment in comparison to predicted IR spectra of structures found in this work and mzCloud. Experimental spectrum of pathway *m/z* 176 → 159 → 115 shown for comparison. Energies are relative to global minimum structure of Figure S13.

2.3.8 MS^3 fragment ion, m/z 113 (via fragment ion m/z 159)

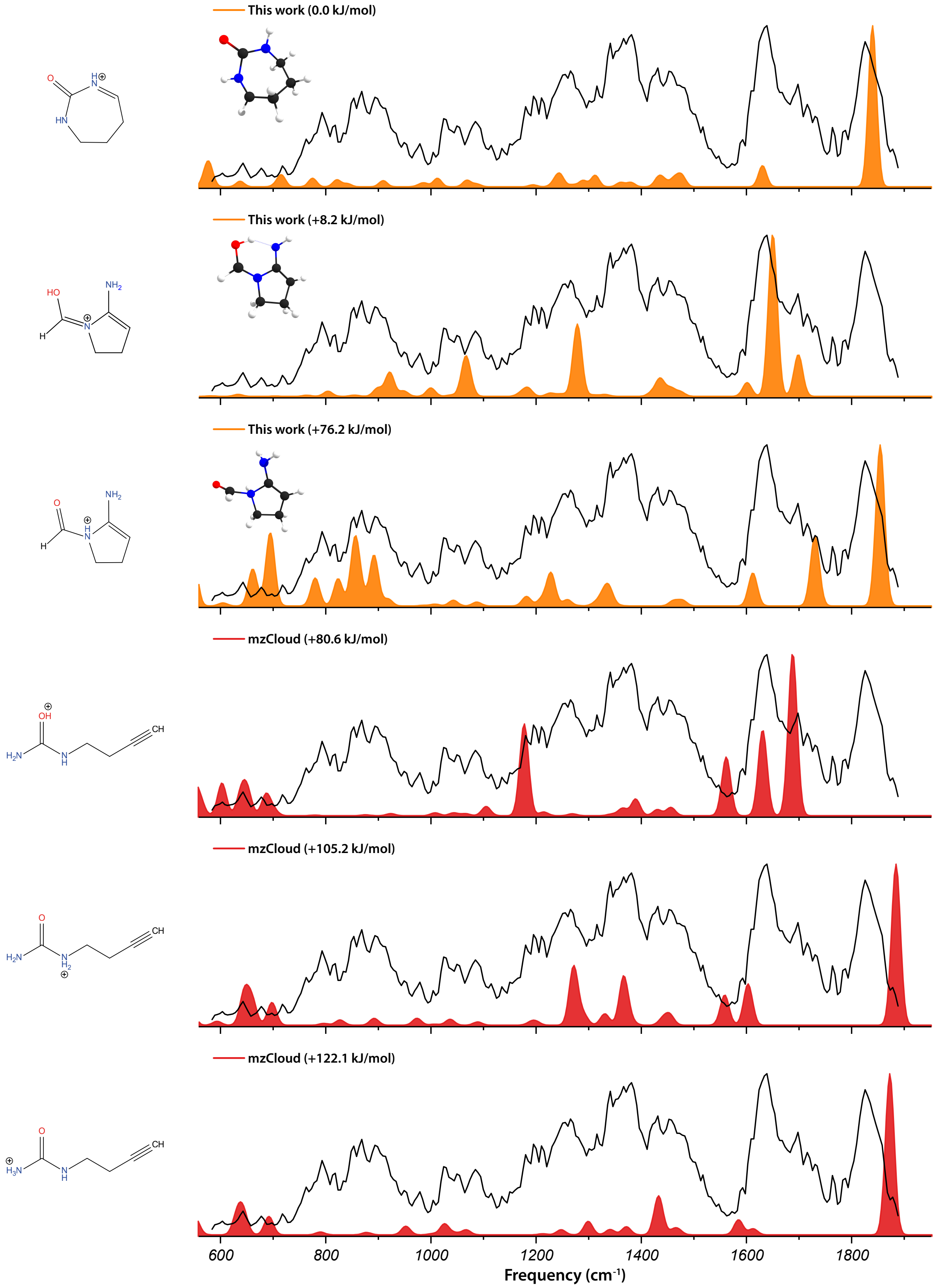

Figure S15: IRIS spectrum of the m/z 113 fragment ion produced via the m/z 159 intermediate in an MS^3 experiment in comparison to predicted IR spectra of structures found in this work and mzCloud.

2.4 Homocitrulline

2.4.1 Precursor ion, m/z 190

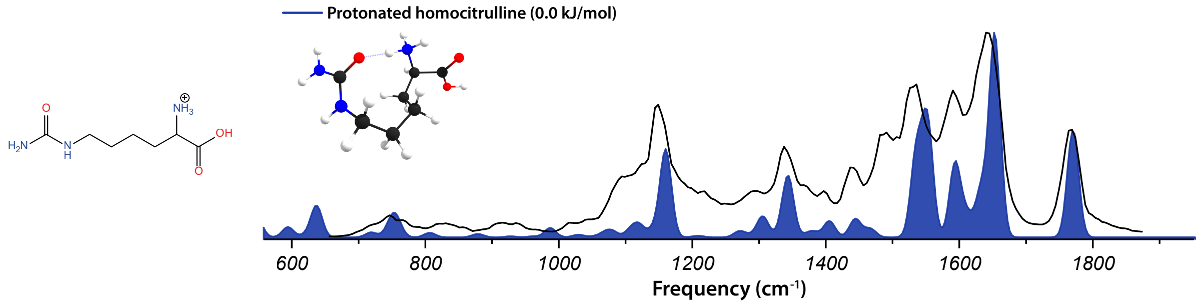

Figure S16: IRIS spectrum of protonated homocitrulline compared with predicted IR spectrum of lowest-energy protonation site.

2.4.2 MS^2 fragment ion, m/z 173

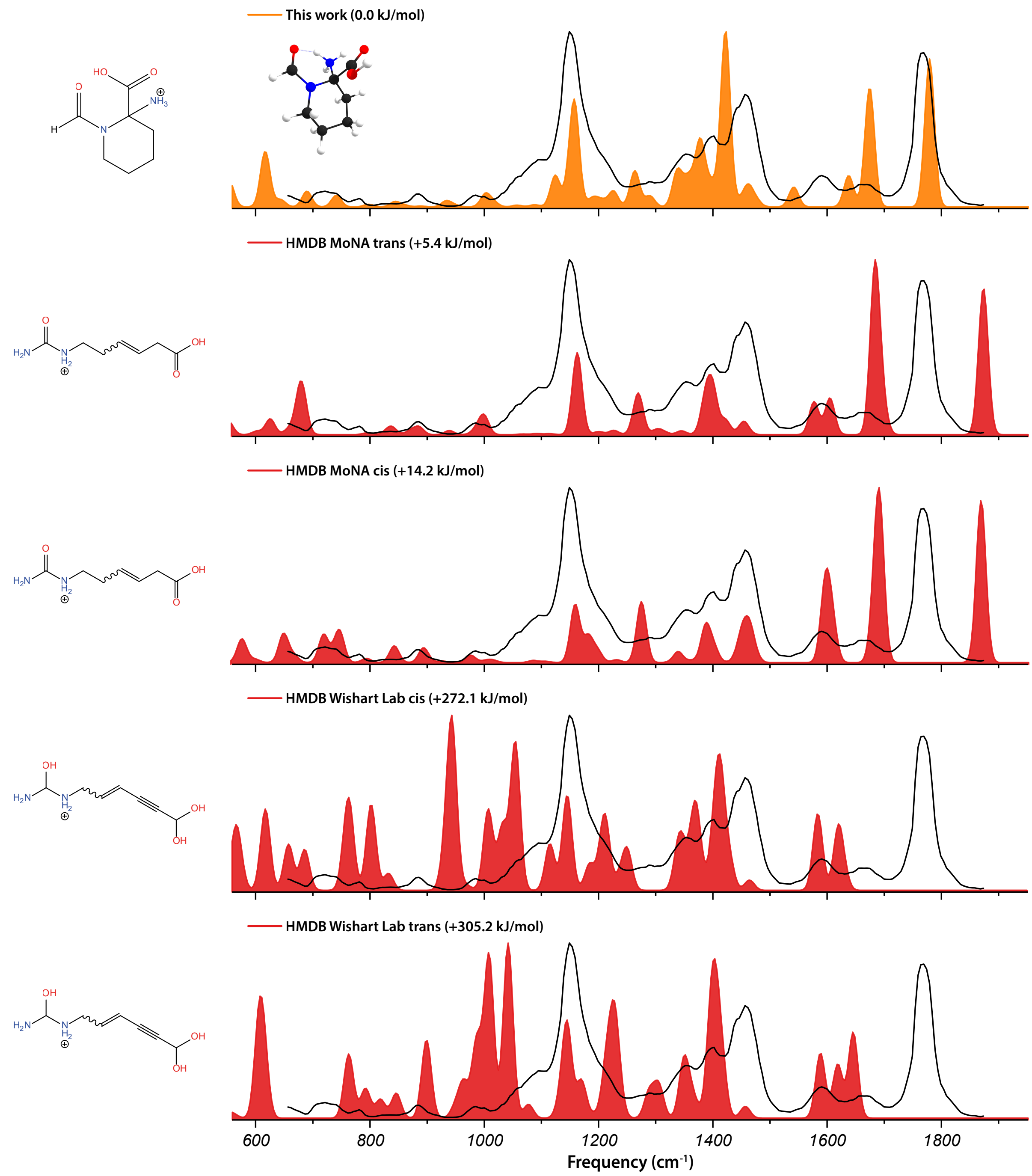

Figure S17: IRIS spectrum of the *m/z* 173 fragment ion produced directly from homocitrulline in comparison to predicted IR spectra of structures found in this work and the HMDB.

2.4.3 MS^2 fragment ion, m/z 147

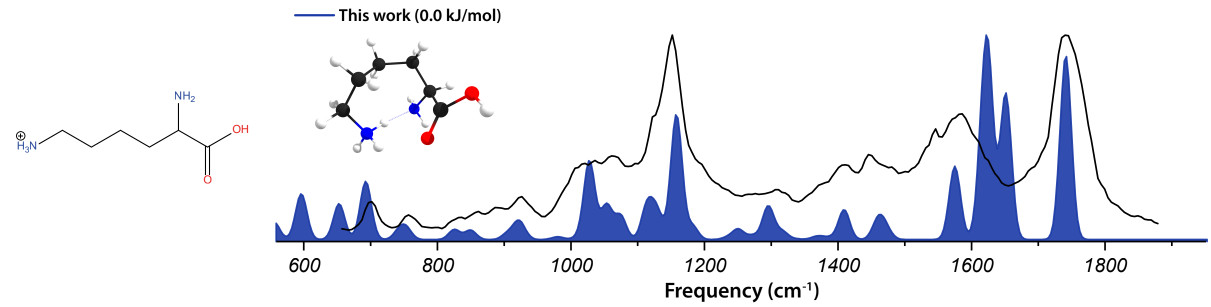

Figure S18: IRIS spectrum of the *m/z* 147 fragment ion produced directly from homocitrulline in comparison to predicted IR spectrum of structure found in this work.

2.4.4 MS^2 fragment ion, m/z 144

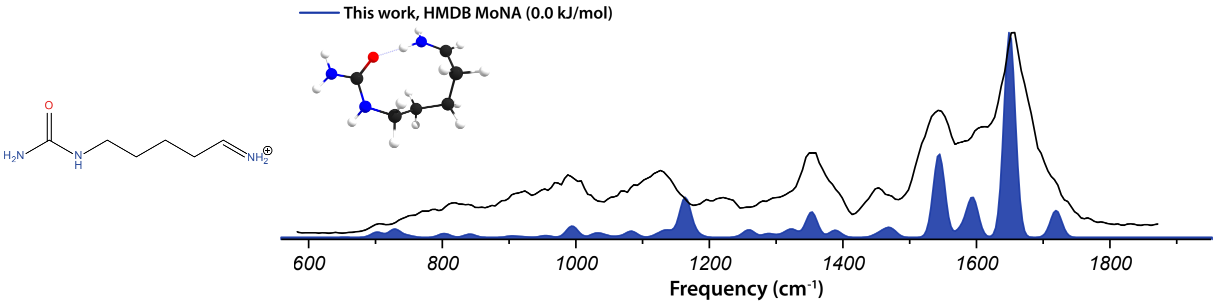

Figure S19: IRIS spectrum of the *m/z* 144 fragment ion produced directly from homocitrulline in comparison to predicted IR spectrum of structure found in this work and the HMDB. The structure proposed in the HMDB is identical to the spectroscopically established structure.

2.4.5 MS^2 and MS^3 fragment ion, m/z 130 (via fragment ion m/z 147)

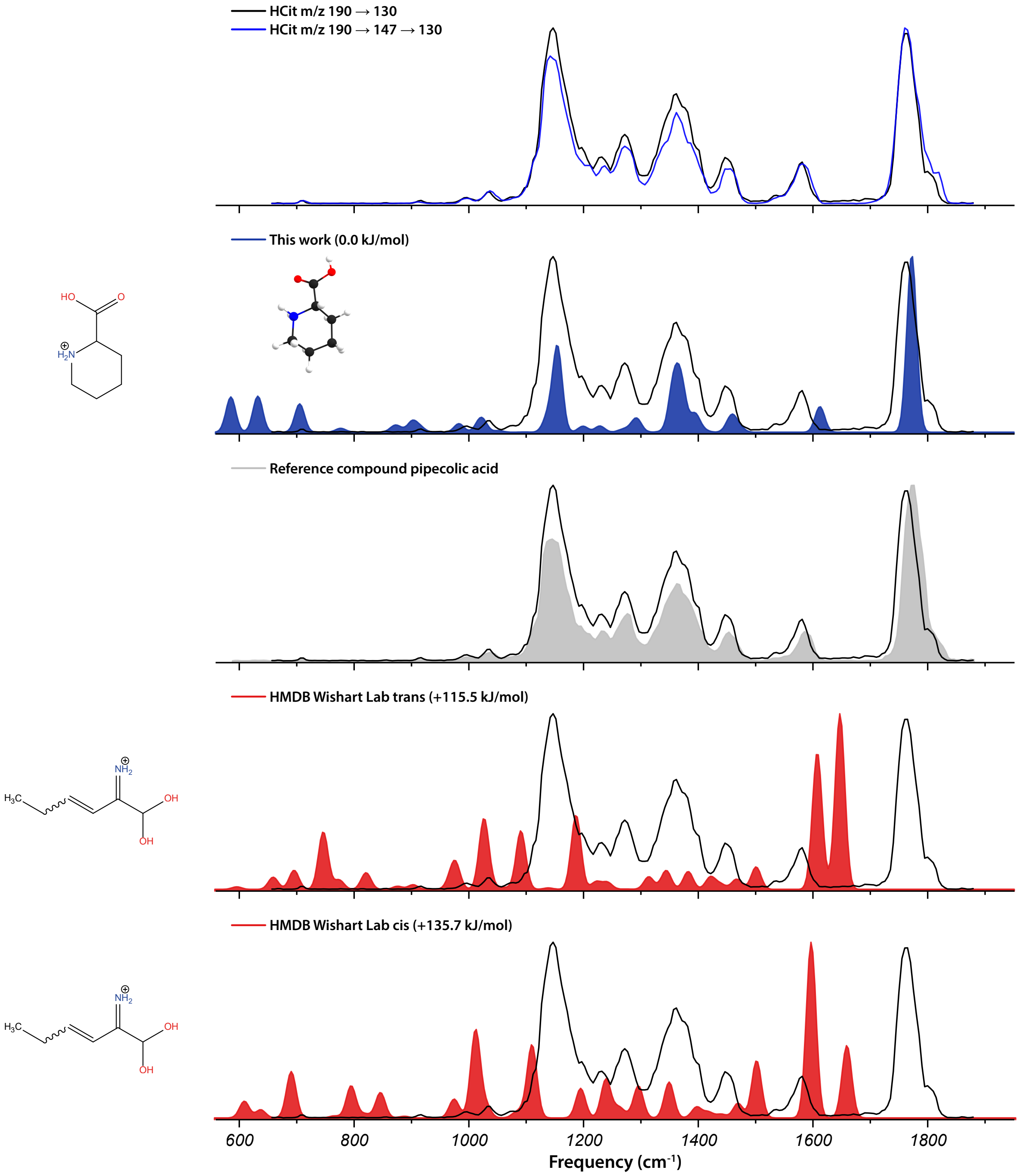

Figure S20: IRIS spectrum of the *m/z* 130 fragment ion produced directly from homocitrulline (MS^2) and via the *m/z* 147 intermediate in an MS^3 experiment in comparison to predicted IR spectra of structures found in this work and the HMDB. Experimental spectrum of reference compound pipecolic acid shown for comparison.

2.4.6 MS^2 and MS^3 fragment ion, m/z 127 (via fragment ion m/z 144)

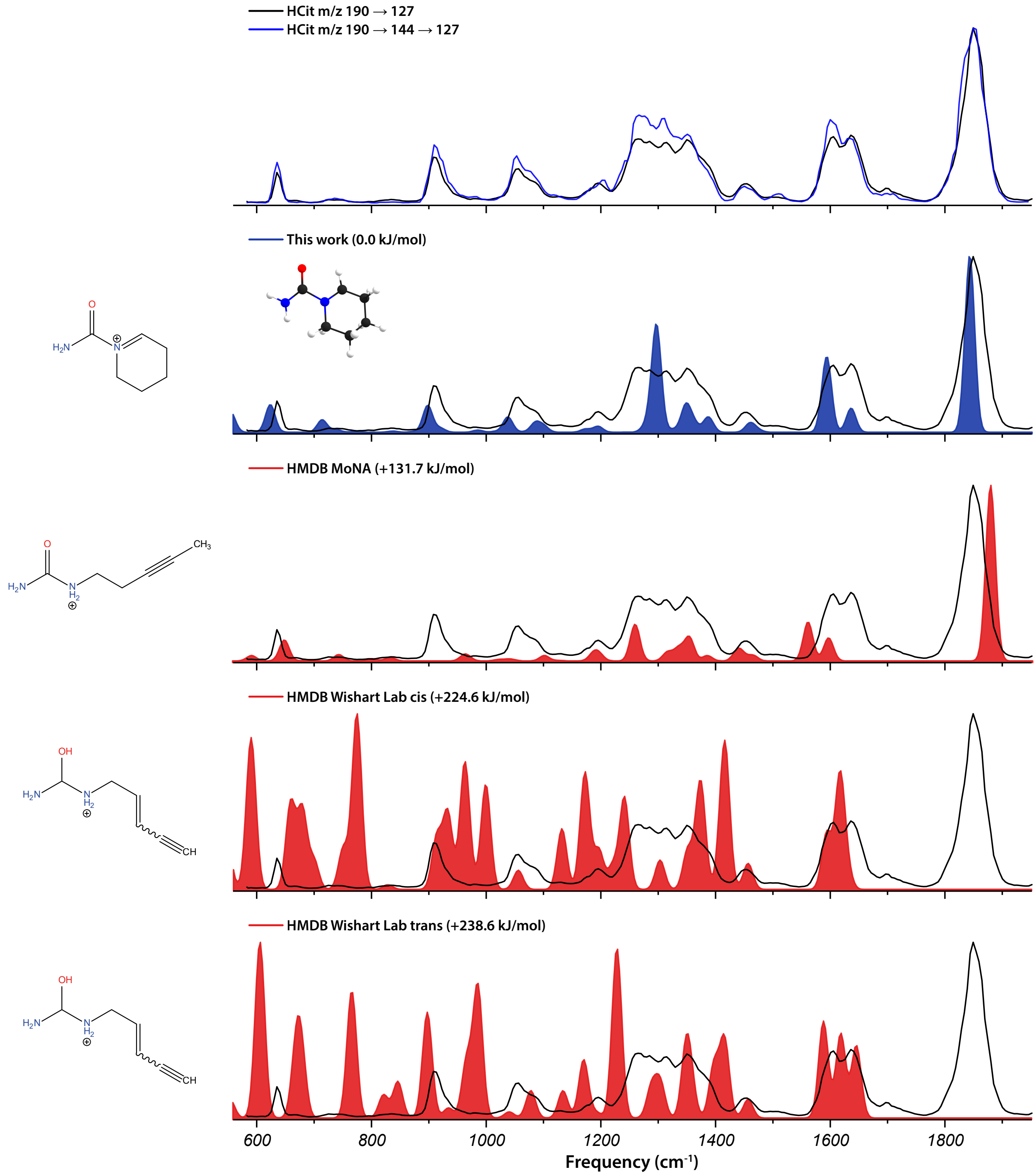

Figure S21: IRIS spectrum of the *m/z* 127 fragment ion produced directly from homocitrulline (MS^2) and via the *m/z* 144 intermediate in an MS^3 experiment in comparison to predicted IR spectra of structures found in this work and the HMDB.

2.4.7 MS^3 fragment ion, m/z 127 (via fragment ion m/z 173)

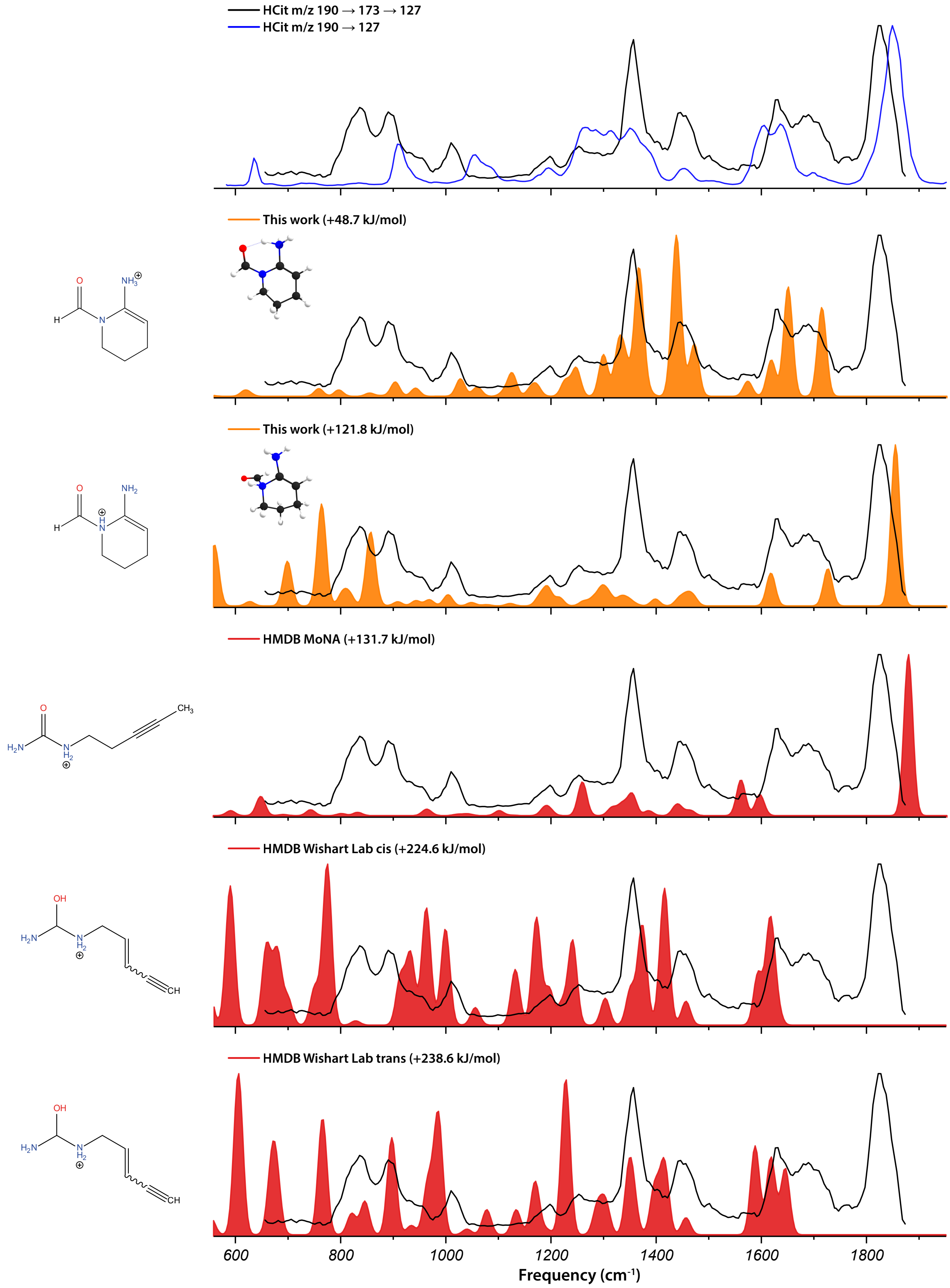

Figure S22: IRIS spectrum of the m/z 127 fragment ion produced via the m/z 173 intermediate in an MS^3 experiment in comparison to predicted IR spectra of structures found in this work and the HMDB. Experimental spectrum of pathway m/z 190 → 127 shown for comparison. Energies are relative to global minimum structure of Figure S21.

2.4.8 MS^n fragment ion, m/z 84

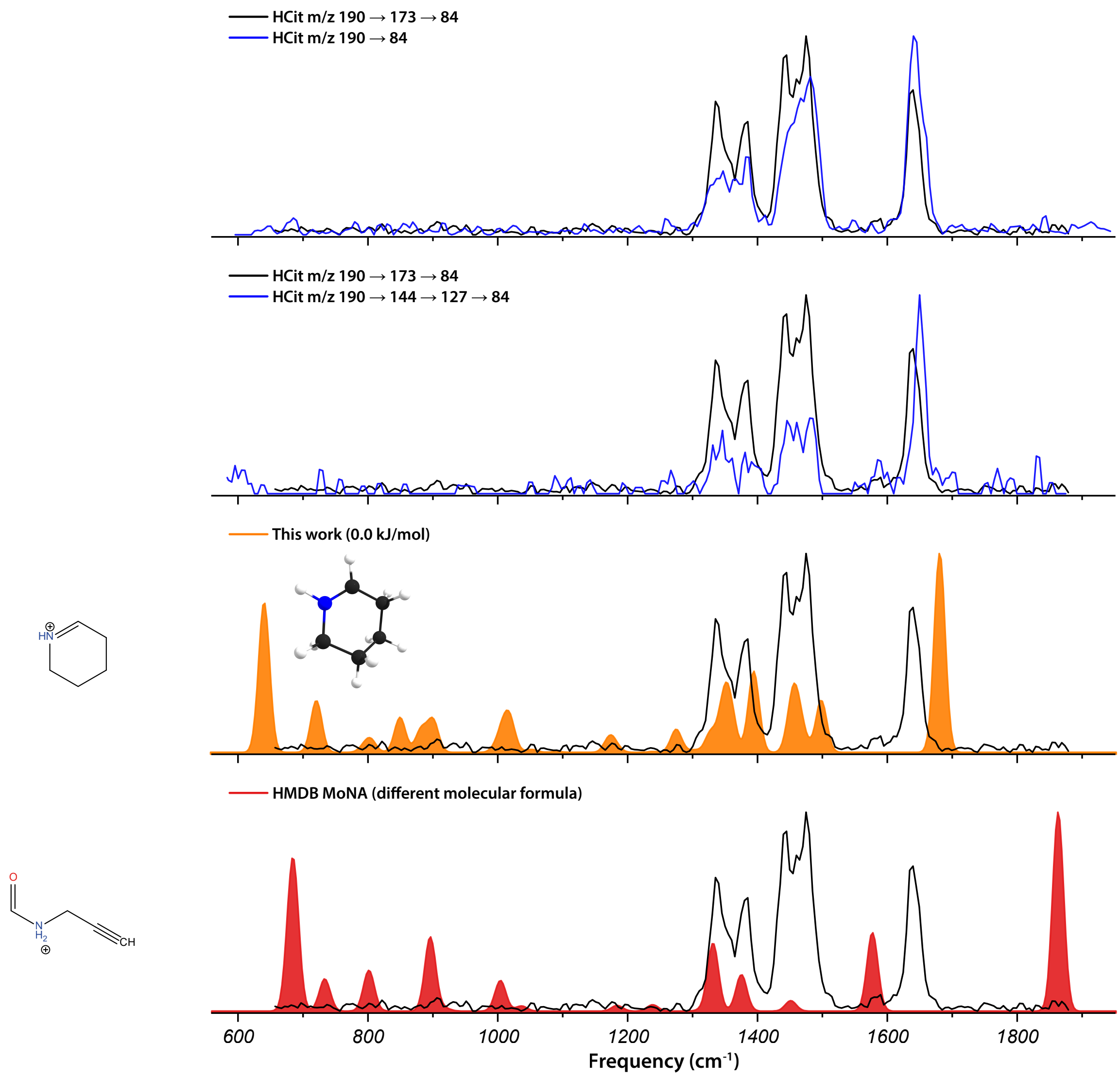

Figure S23: IRIS spectrum of the *m/z* 84 fragment ion produced directly from homocitrulline and via pathways *m/z* 190 → 173 → 84, *m/z* 190 → 84 and *m/z* 190 → 144 → 127 → 84 in comparison to predicted IR spectra of structures found in this work and the HMDB.

2.4.9 MS^4 fragment ion, m/z 84 (via MS^3 fragment ion m/z 190 → 173 → 127)

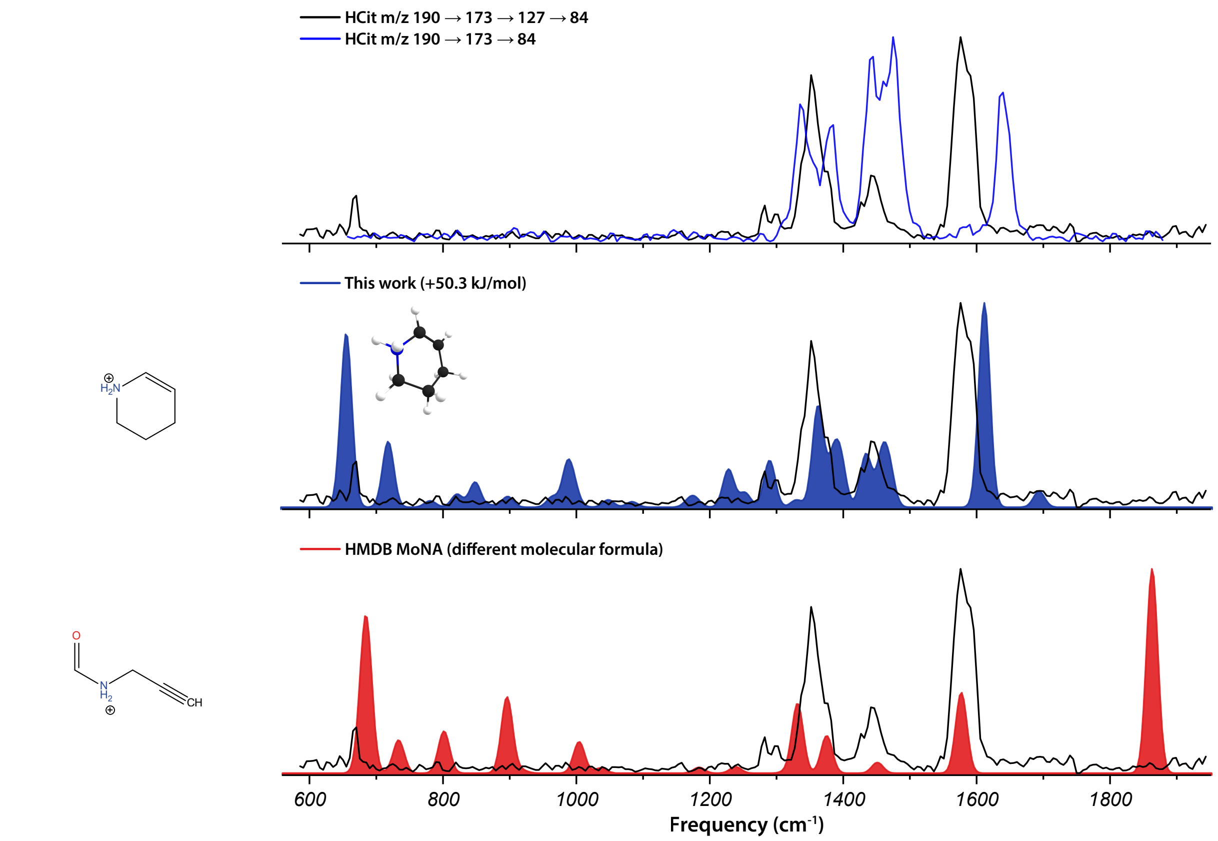

Figure S24: IRIS spectrum of the *m/z* 84 fragment ion produced via the pathway *m/z* 190 → 173 → 127 in comparison to predicted IR spectra of structures found in this work and the HMDB. Experimental spectrum of pathway *m/z* 190 → 173 → 84 shown for comparison. Energies are relative to global minimum structure of Figure S23.

2.5 Arginine

2.5.1 Precursor ion, m/z 175

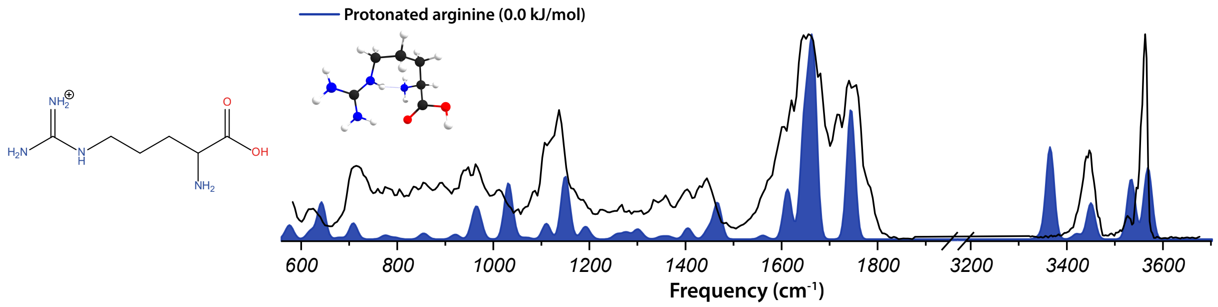

Figure S25: IRIS spectrum of protonated arginine compared with predicted IR spectrum of lowest-energy protonation site.

2.5.2 MS^2 fragment ion, m/z 158

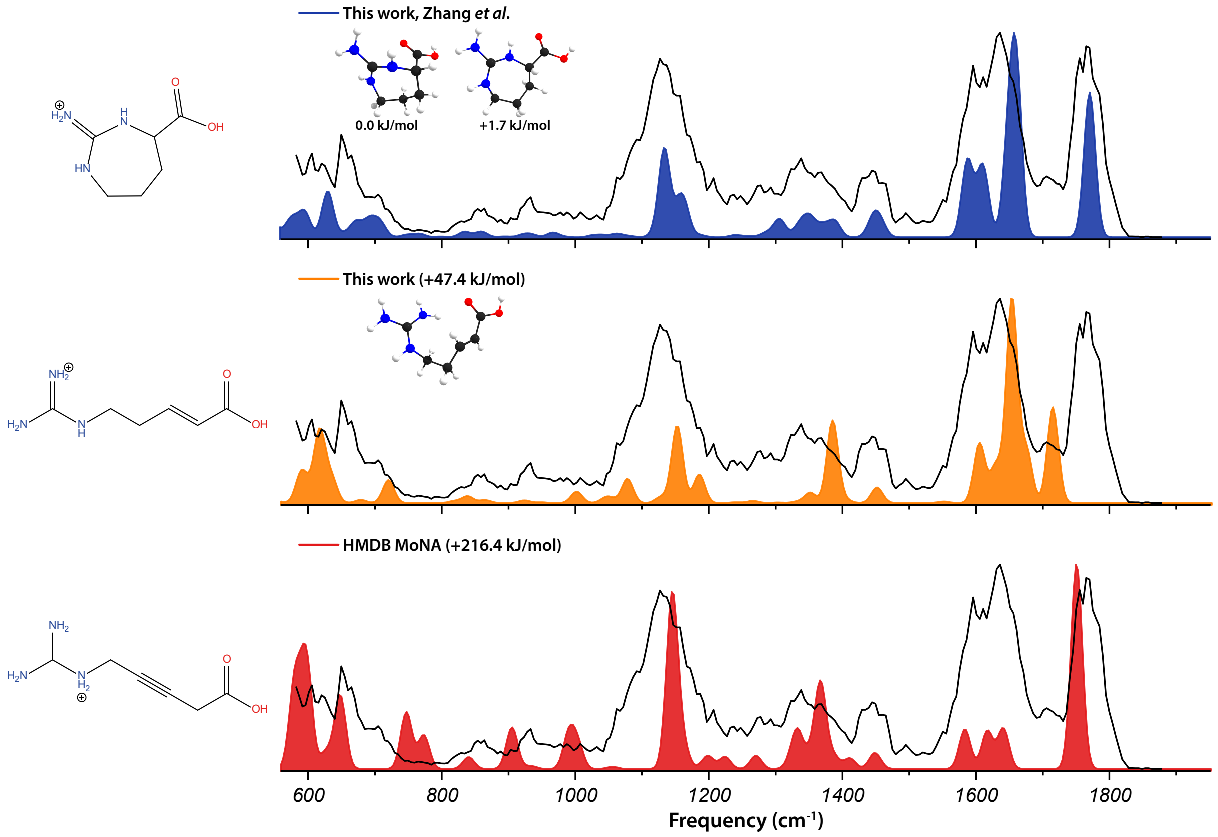

Figure S26: IRIS spectrum of the m/z 158 fragment ion produced directly from arginine in comparison to predicted IR spectra of structures found in this work, the HMDB and Zhang *et al.* [1]. The structure proposed by Zhang *et al.* is identical to the spectroscopically established structure.

2.5.3 MS^2 fragment ion, m/z 157

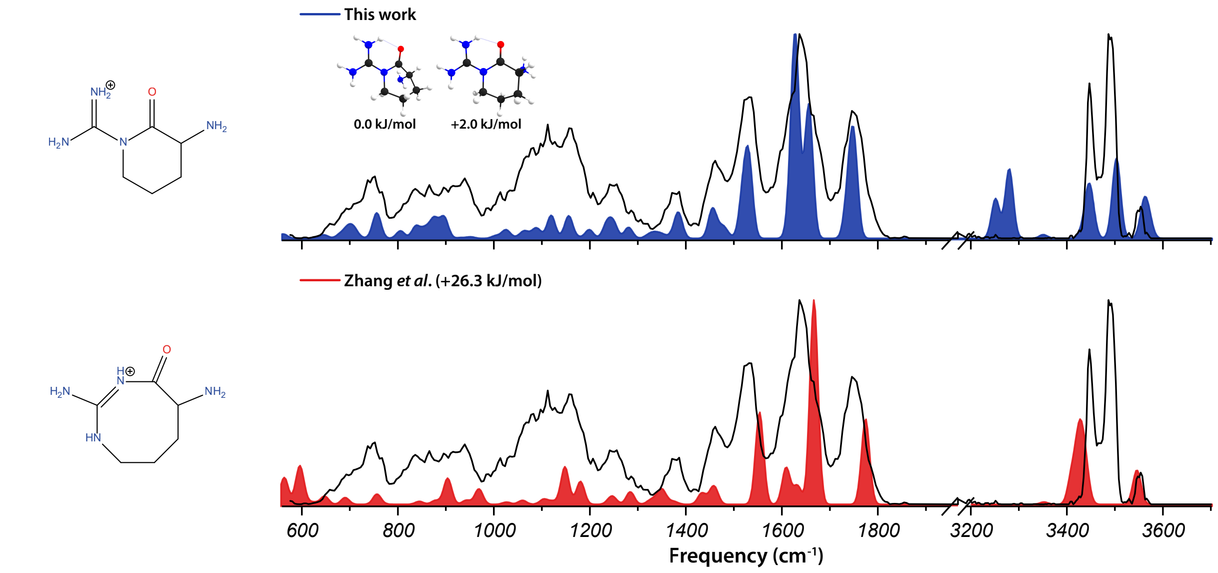

Figure S27: IRIS spectrum of the m/z 157 fragment ion produced directly from arginine in comparison to predicted IR spectra of structures found in this work and Zhang *et al.* [1].

2.5.4 MS^3 fragment ion, m/z 140 (via fragment ion m/z 158 and fragment ion m/z 157)

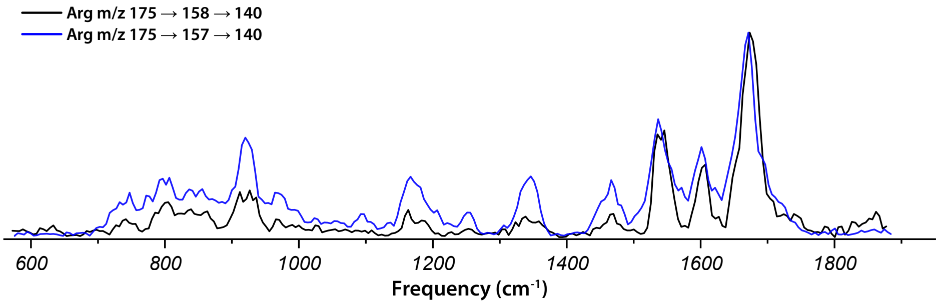

Figure S28: IRIS spectrum of the *m/z* 140 fragment ion produced via the *m/z* 158 intermediate in an MS^3 experiment and via the *m/z* 157 intermediate in an MS^3 experiment. No matching structures found.

2.5.5 MS^2 fragment ion, m/z 130

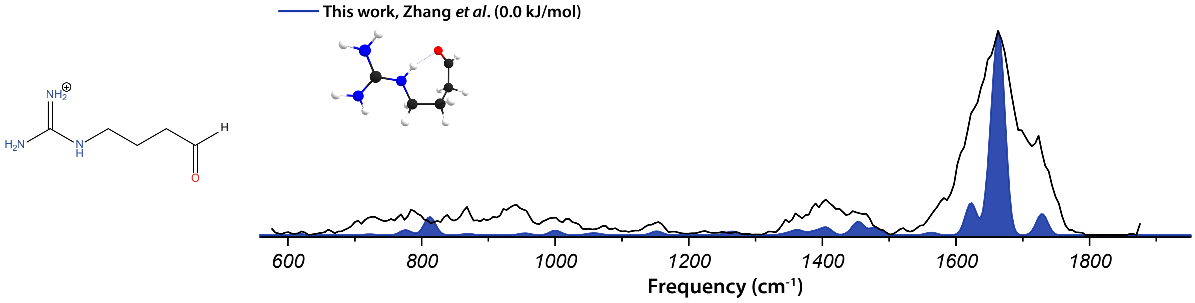

Figure S29: IRIS spectrum of the *m/z* 130 fragment ion produced directly from arginine in comparison to predicted IR spectrum of structure found in this work and Zhang *et al.* [1]. The structure proposed by Zhang *et al.* is identical to the spectroscopically established structure.

2.5.6 MS^2 and MS^3 fragment ion, m/z 116 (via fragment ion m/z 158)

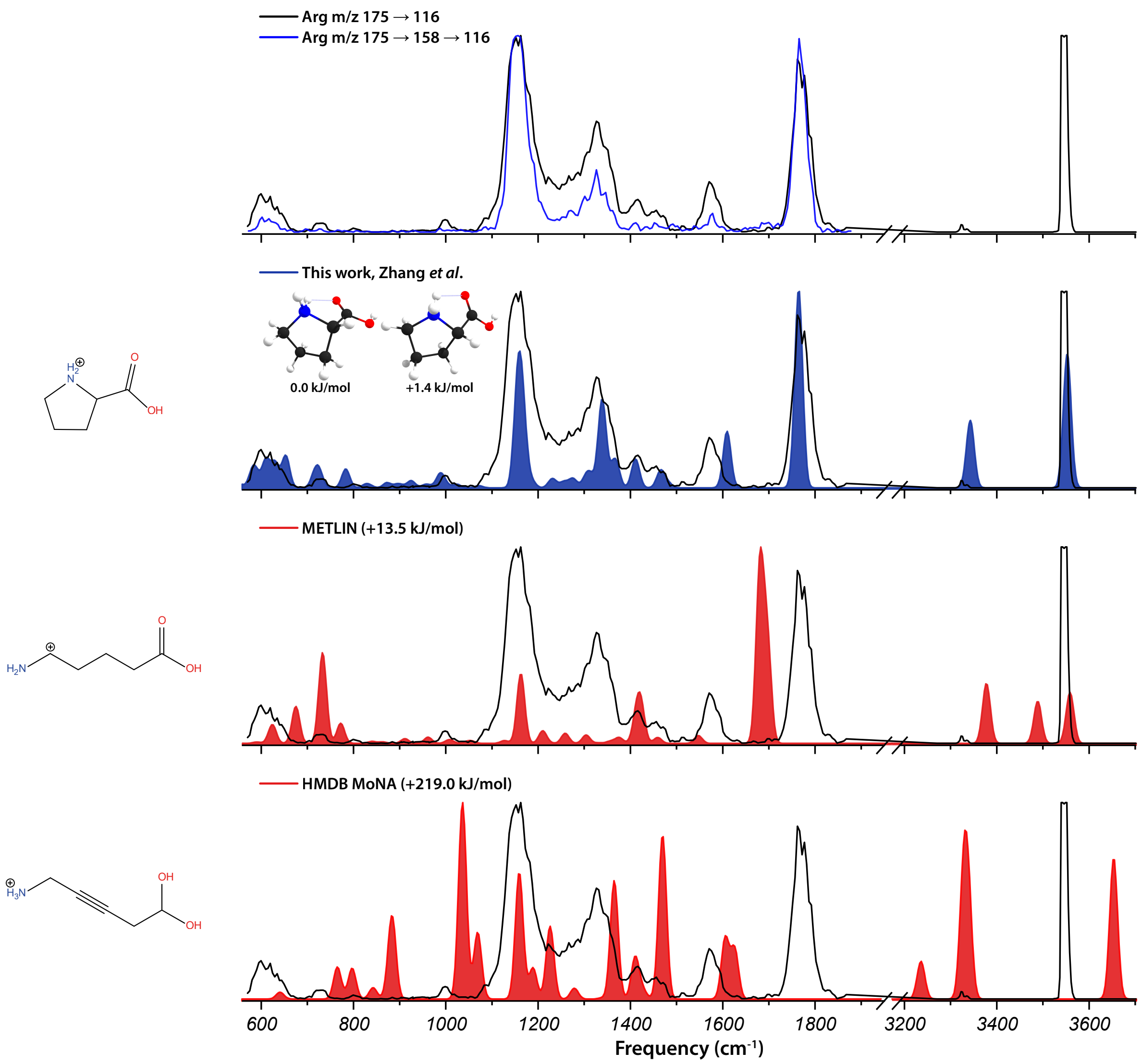

Figure S30: IRIS spectrum of the *m/z* 116 fragment ion produced directly from arginine (MS^2) and via the *m/z* 158 intermediate in an MS^3 experiment in comparison to predicted IR spectra of structures found in this work, Zhang *et al.* [1], the HMDB and METLIN. The structure proposed by Zhang *et al.* is identical to the spectroscopically established structure.

2.5.7 MS^3 fragment ion, m/z 115 (via fragment ion m/z 157)

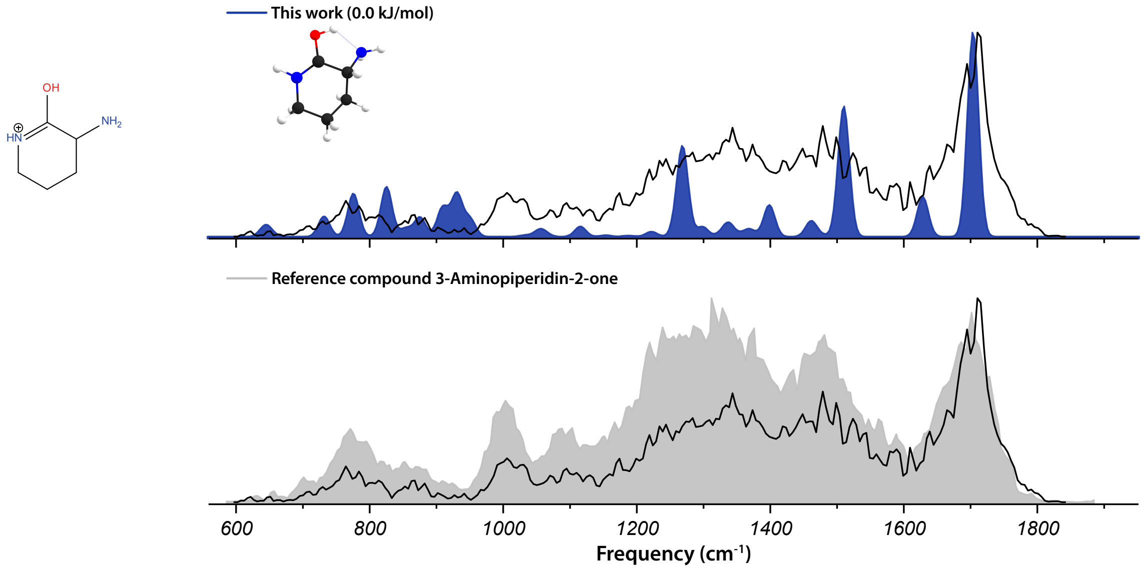

Figure S31: IRIS spectrum of the *m/z* 115 fragment ion produced via the *m/z* 157 intermediate in an MS^3 experiment in comparison to predicted IR spectrum of structure found in this work. Experimental spectrum of reference compound 3-Aminopiperidin-2-one shown for comparison. The reference compound spectrum was recorded at a different time and hence the difference in intensity due to a difference in laser power.

2.5.8 MS^n fragment ion, m/z 112

The arginine fragment of m/z 112 was generated via different MS^n paths. As detailed in the main text as well as in Section 2.7, the experimental IR spectrum suggests that the m/z 112 fragment ion adopts two different structures, here named structure A and structure B.

MS^n fragment ion, m/z 112 (structure A)

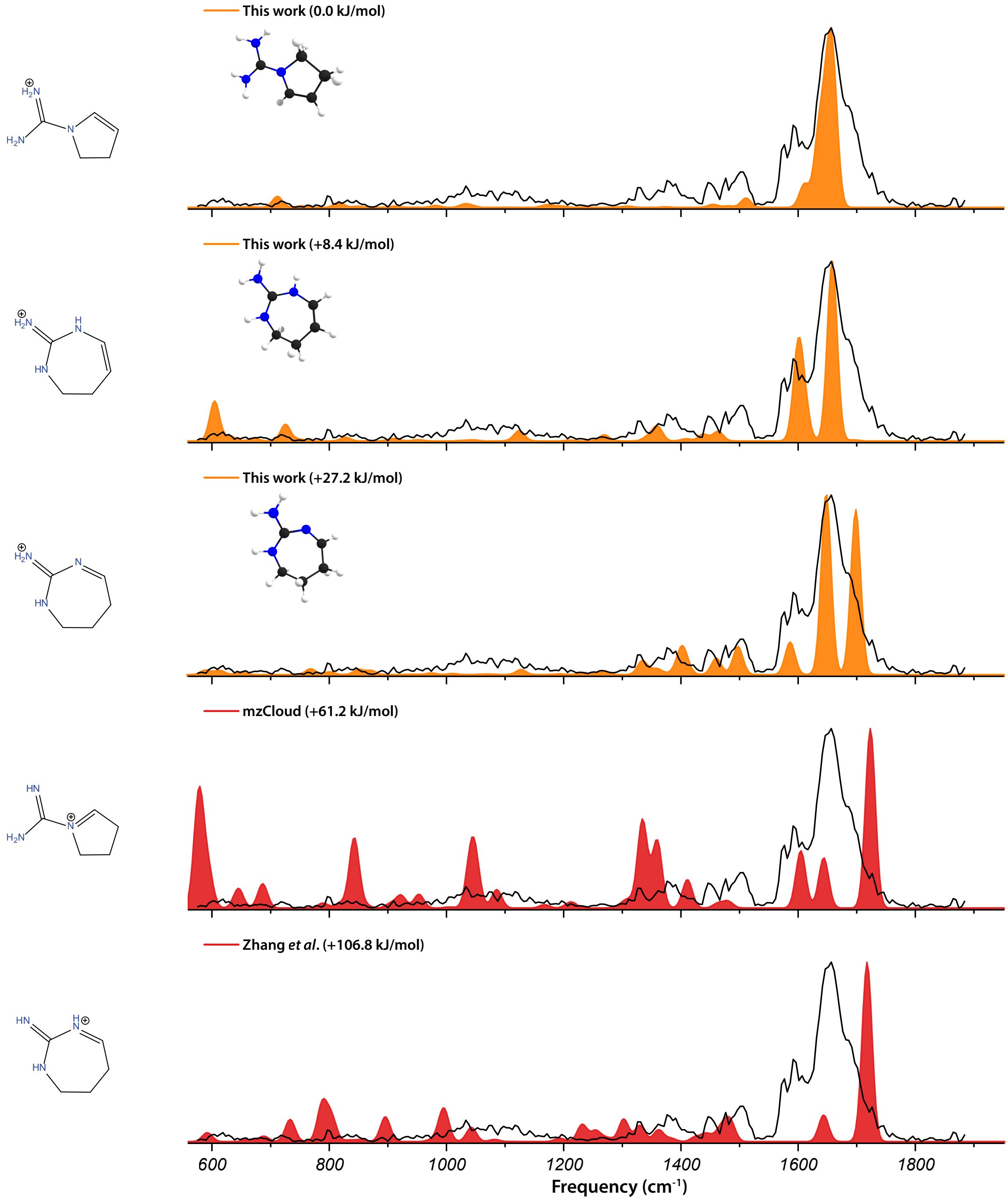

Figure S32: IRIS spectrum of the m/z 112 fragment ion produced via the m/z 158 intermediate in an MS^3 experiment in comparison to predicted IR spectra of structures found in this work, Zhang *et al.* [1] and mzCloud.

MS^n fragment ion, m/z 112 (structure B)

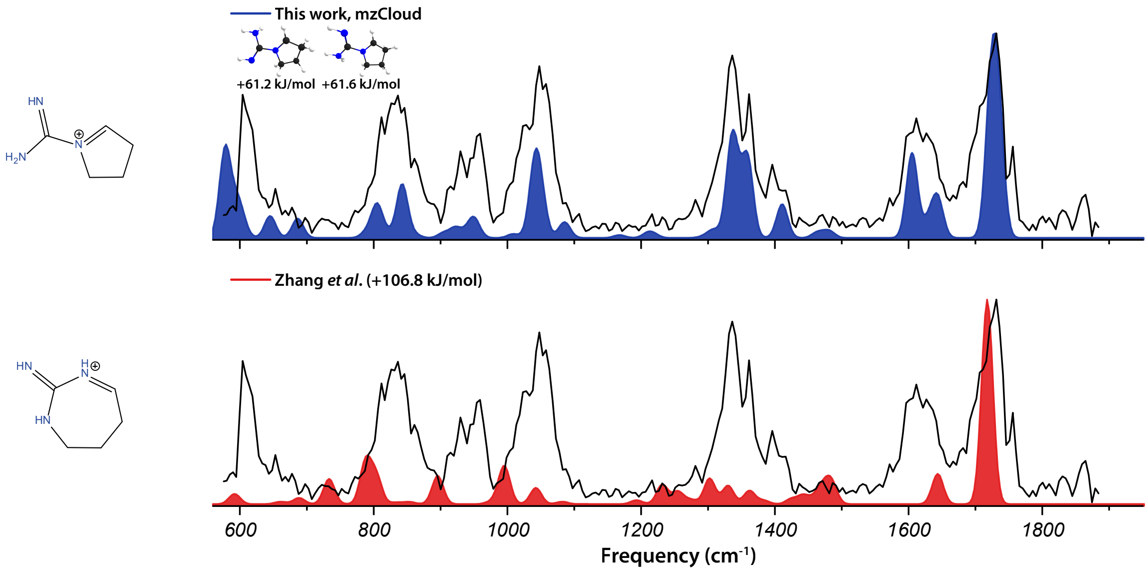

Figure S33: IRIS spectrum of the *m/z* 112 fragment ion produced directly from arginine in comparison to predicted IR spectra of structures found in this work, Zhang *et al.* [1] and mzCloud. The structure proposed in mzCloud is identical to the spectroscopically established structure. Energies are relative to global minimum structure of Figure S32.

2.6 Supplementary Note 1: Comparison of MS^2 fragment ion m/z 116 of arginine and citrulline

Arginine and citrulline both form a fragment ion of m/z 116 ($C_5H_{10}NO_2^+$) and their corresponding infrared spectra are compared in Figure S34. A clear difference is seen as the spectrum of the citrulline fragment contains a peak at 1700 cm^{-1} that is absent in the arginine fragment ion spectrum, which is the result of a difference in dissociation products. Both fragment ions of m/z 116 dissociate into an ion of m/z 70, but the ion originating from citrulline also forms fragment ions with an m/z value of 71 and 98, giving rise to the additional peak around 1700 cm^{-1}. Therefore, it appears that the citrulline fragment ion of m/z 116 consists of two different structures. One structure (structure I) only dissociates into m/z 70 and is equal to the structure of arginine, whereas structure II fragments into m/z 71 and m/z 98.

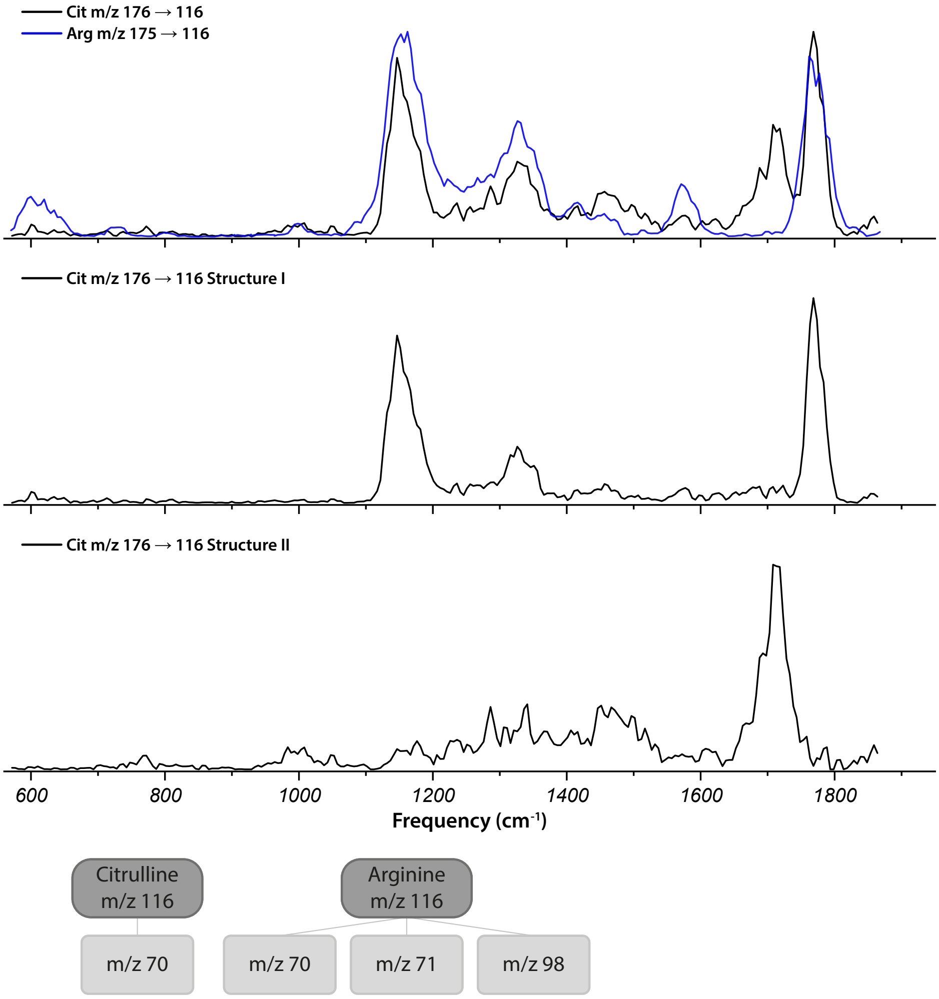

Figure S34: IRIS spectrum of the m/z 116 fragment ion produced directly from citrulline in comparison to the IRIS spectrum of the m/z 116 fragment ion produced directly from arginine. An additional peak is seen in the spectrum of the arginine fragment, which is due to the presence of an isomeric ion (see text of Supplementary Note 1).

2.7 Supplementary Note 2: Analysis of arginine MS^n fragment ion at m/z 112

In Figure S35, the experimental infrared spectrum from the arginine m/z 112 fragment ion directly formed from the parent ion (m/z 175) is shown in black in all graphs and compared to infrared spectra recorded for the same m/z value but obtained via different MS^n pathways. As not all infrared spectra show the same features, an analysis is performed to study the number of different structures. For instance, the spectrum of m/z 175 → 158 → 112 fragment shows a peak at 1650 cm^{-1}, while the m/z 175 → 157 → 112 fragment spectrum clearly lacks this peak. Simultaneously, the sum of these two spectra seems to produce the spectrum of the direct pathway m/z 175 → 112. This could indicate that the direct path (m/z 175 → 112) is a mixture of the structures formed by pathway m/z 175 → 158 → 112 (structure A) and pathway

m/z 175 → 157 → 112 (structure B). Moreover, the spectra of the m/z 175 → 157 → 112 fragment ion and the m/z 175 → 157 → 140 → 112 fragment ion share the same features (of structure B), whereas the m/z 175 → 112 spectrum and m/z 175 → 130 → 112 spectrum match very well, indicating a mixture of both structure A and B.

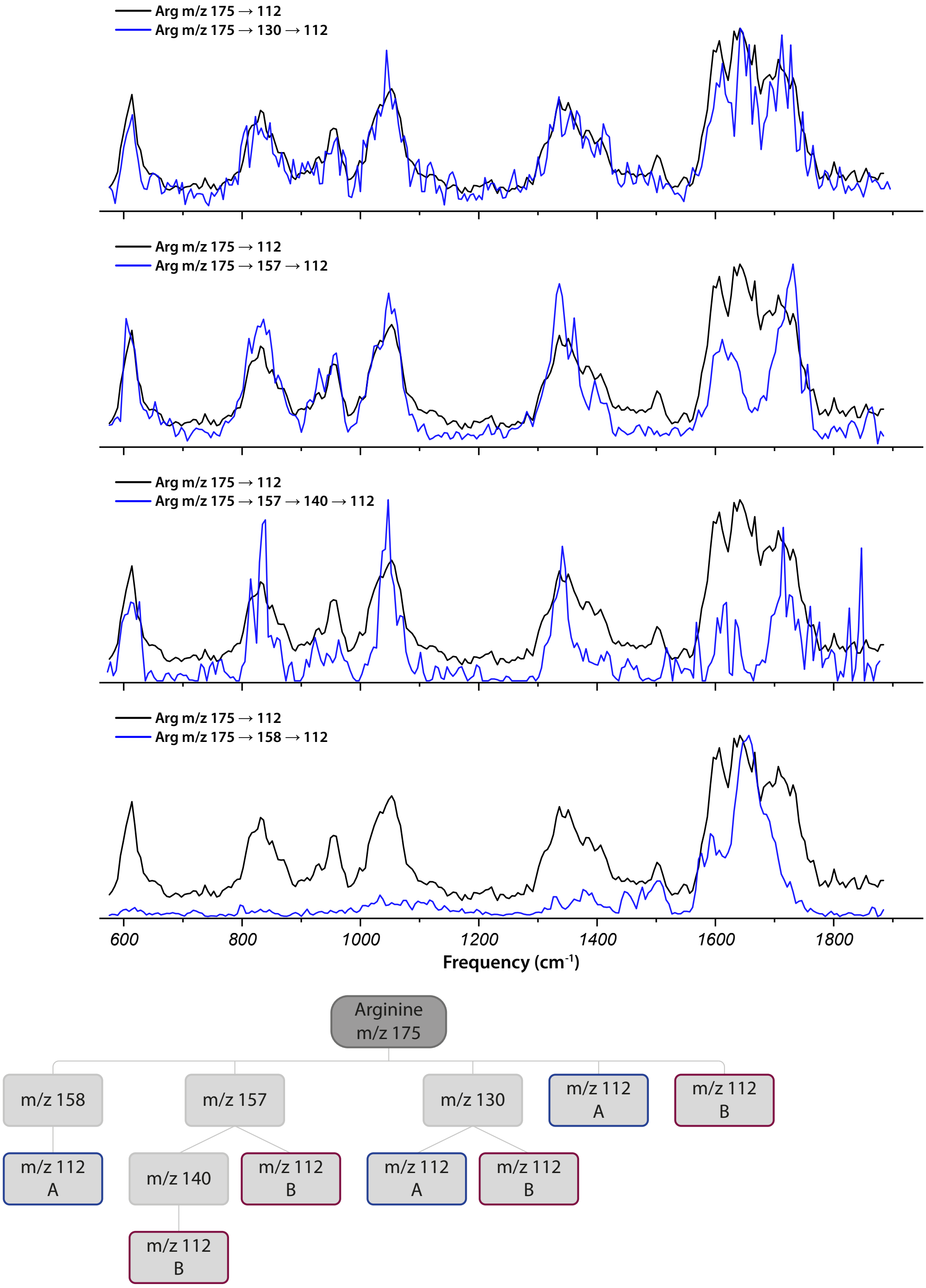

Figure S35: IRIS spectrum of the m/z 112 fragment ion produced directly from arginine (m/z 175 → 112) in comparison to spectra of the m/z 112 fragment ion produced via pathways m/z 175 → 130 → 112, m/z 175 → 157 → 112, m/z 175 → 157 → 140 → 112 and m/z 175 → 158 → 112. Analysis indicates the formation of two distinct structures, A and B, displayed in blue and red in the diagram.

3 Optimized geometries of fragment ion structures

All optimized geometries are given as xyz atom positions.

3.1 α-Amino-adipic acid

3.1.1 Precursor ion, m/z 162

Protonated α-amino-adipic acid (0.0 kJ/mol)

N	−1.2620097654	1.0133927912	1.0030838606	H	−1.8152965350	1.8715330853	0.9607740446
C	−1.6317000559	0.0400360461	−0.0958878151	H	−1.4547077750	0.5469317427	1.9069043927
C	−0.4479944971	−0.2165738486	−1.0473225291	H	−0.2351239098	1.2765590871	0.9481897147
C	0.7921416064	−0.8807748663	−0.3865710933	H	−2.4656893285	0.4661400002	−0.6612921594
C	2.1139296446	−0.1437330586	−0.6335324224	H	−0.1849823297	0.7420410223	−1.5081785618
C	2.2129992303	1.1976903037	0.0521323366	H	−0.8308131948	−0.8430612722	−1.8571731401
O	3.3910555942	1.7806720103	−0.1239759409	H	0.6533455352	−0.9952652811	0.6956993418
O	1.3197804989	1.7282349813	0.7154093065	H	0.9007934394	−1.8975788410	−0.7730274243
C	−2.1420980290	−1.2164204404	0.6198937926	H	2.9626334844	−0.7449118832	−0.2869719651
O	−2.1691545242	−1.3073617262	1.8304368528	H	2.2937007718	0.0177045946	−1.7041793433
O	−2.5374234939	−2.1481861028	−0.2407227905	H	3.4099580621	2.6372431954	0.3410303079
				H	−2.8731444290	−2.9243115398	0.2451812387

Protonated α-amino-adipic acid (+1.7 kJ/mol)

N	−0.2673410952	0.9284275627	−1.5898509996	H	−0.2014291250	0.6931866749	−2.5825569184
C	−1.2899616146	0.0850276278	−0.8612929470	H	−0.5908417562	1.9099766488	−1.5278488833
C	−0.7328747853	−0.4137629637	0.4855069536	H	−1.5541081139	−0.7648748554	−1.4969182420
C	0.4173971516	−1.4492844404	0.3395639365	H	−1.5711140436	−0.8564382092	1.0294425169
C	1.6826196220	−1.1193498835	1.1400939044	H	−0.4145983117	0.4561222915	1.0730834476
C	2.4481418421	0.0748135318	0.6233551060	H	0.0579634876	−2.4272309668	0.6708520883
O	2.1039347744	0.7881742468	−0.3208630858	H	0.6928058470	−1.5819914387	−0.7139087880
O	3.5703634978	0.2976301116	1.2938080938	H	1.4575277100	−0.9377790326	2.1988014760
C	−2.5202263991	0.9839958734	−0.6917709567	H	2.3746119281	−1.9697638988	1.1359063545
O	−2.4937487657	2.1699157374	−0.9499771158	H	0.6923566469	0.8421837620	−1.1459316884
O	−3.5661012223	0.3085605160	−0.2280635111	H	4.0307325347	1.0720837708	0.9214283752
				H	−4.3261098091	0.9101773339	−0.1199591167

3.1.2 MS^2 fragment ion, m/z 144

This work (0.0 kJ/mol)

O	2.3948587309	1.6306523086	0.3456929346	H	3.2649734941	1.2537235001	0.5571926120
C	1.4847564933	0.7176661471	0.0836804967	H	2.0222309722	−0.9464520516	1.2216666671
C	1.7934277417	−0.7455303448	0.1644721996	H	2.7177994626	−0.9371047355	−0.3944267692
C	0.6380998297	−1.6377284073	−0.3268545688	H	0.7696517839	−2.6428572167	0.0804559780
C	−0.7204713794	−1.0621981584	0.0938152304	H	0.6822833754	−1.7293144119	−1.4178461151
C	−0.8837953074	0.3402616941	−0.5012467208	H	−1.5374421480	−1.6909192258	−0.2659803394
C	−2.0906049680	1.1427592779	−0.0046885689	H	−0.8020656473	−1.0141391549	1.1864830982
O	−1.9997933541	2.2879489913	0.3787726048	H	−0.9798354573	0.2679154328	−1.5934355639
O	−3.2139539379	0.4323428454	−0.0946809272	H	−3.9690311152	0.9811129546	0.1873846931
N	0.3014808046	1.1721535465	−0.2074489218	H	0.1273306266	2.1797070085	−0.1439080195

This work (+1.4 kJ/mol)

O	2.4357843329	1.6239073250	−0.0503426094	H	3.2480563487	1.2582852203	0.3378488231
C	1.4573145761	0.7502403041	−0.1241464135	H	2.3061584381	−1.1258634830	−0.4622545338
C	1.6425550093	−0.6747705441	0.2909000601	H	2.1931240601	−0.6870855665	1.2392823201
C	0.3185092831	−1.4525404832	0.3964332618	H	−0.1484253803	−1.2450364627	1.3644517318
C	−0.6259256467	−1.0698795665	−0.7487748265	H	0.5331438112	−2.5232835893	0.3688677013
C	−0.9142664306	0.4424893463	−0.7395878207	H	−1.5760704925	−1.6032151274	−0.6699793236
C	−1.8172314760	0.8540524807	0.4367833953	H	−0.1871427244	−1.3348758591	−1.7174608659
O	−1.4113379358	1.3954243835	1.4385736986	H	−1.3946330796	0.7407012510	−1.6753176888
O	−3.0839612105	0.5018620100	0.1931501642	H	−3.6415219646	0.7478101020	0.9539986424
N	0.3448364978	1.2036595774	−0.6311091058	H	0.3211339837	2.1980186815	−0.8522166106

METLIN (+78.5 kJ/mol)

O	−3.7468779803	1.2511159438	0.3056642902	H	−3.0443075772	1.4434214943	−1.5939013466
C	−2.9143481459	1.1046360357	−0.5530772108	H	−0.5766301623	−0.3996352361	1.3924576512
C	−1.5848229836	0.4078217920	−0.2044355763	H	−2.2673526814	0.2022139358	1.6365263184
N	−1.4758157946	0.0170729192	1.0122483823	H	−0.2166590125	1.2395694185	−1.5459743277
C	−0.5590530086	0.2279609419	−1.2684492809	H	−1.1236591479	−0.1019762528	−2.1541518823
C	0.6351830862	−0.7274802540	−1.0549902105	H	0.2870078944	−1.6672100593	−0.6121608883
C	1.8702709998	−0.2021505147	−0.2929562934	H	0.9770349387	−0.9864250800	−2.0606066220
C	1.8843299767	−0.4462175821	1.1989283308	H	2.0187984596	0.8752697835	−0.4479455275
O	3.1012240132	−0.3604704970	1.7160716930	H	2.7738161165	−0.6715276635	−0.6939377805
O	0.8981009489	−0.6982791103	1.8905949765	H	3.0635600605	−0.5077100148	2.6793953045

METLIN (+127.5 kJ/mol)

N	−2.6233046819	−0.4379671045	0.9407177687	H	−3.3266205865	−0.0552054852	0.3201729402
C	−1.2738896857	−0.3454860422	0.5247082167	H	−2.9153139741	−1.2968928341	1.3905103888
C	−0.9806353529	−1.2826367154	−0.6634762005	H	−0.6133628797	−0.7225358995	1.3113644541
O	−1.3449561610	−1.9423021511	−1.5201417036	H	−0.8255961620	1.6565459604	1.0613131556
C	−0.8785822486	1.0986659392	0.1212433327	H	−1.7134758491	1.5215642024	−0.4485130291
C	0.4083821210	1.2795640124	−0.7001561802	H	0.2822461217	0.8689695666	−1.7102302978
C	1.7060491227	0.7346810365	−0.0857510298	H	0.5395496277	2.3562760051	−0.8415831894
C	1.8863503556	−0.7533555026	−0.2067861411	H	1.7864863715	0.9837515755	0.9823890264
O	3.1261804606	−1.1563512347	−0.0172882138	H	2.5806410569	1.1979589636	−0.5539246983
O	0.9897363977	−1.5780183001	−0.4585898557	H	3.1902159463	−2.1272259923	−0.0968787437

mzCloud (+132.0 kJ/mol)

N	−1.4030110146	1.3869273492	−0.6169886947	H	−1.3847927896	2.3818473602	−0.3727625812
C	−1.6861467947	0.5001743258	0.5325641697	H	−2.0681395480	1.2661635180	−1.3899958443
C	−0.8426191177	−0.7469690800	0.6922976144	H	−0.4274931387	1.1243572359	−0.9961485221
C	0.6506458126	−0.4834501731	1.0627937939	H	−1.3129764793	−1.3587858650	1.4671263061
C	1.6789561416	−0.9821153942	0.0399464946	H	−0.9184751149	−1.3457170464	−0.2261295800
C	1.7104129832	−0.2315000928	−1.2672945475	H	0.8684713522	−0.9870218505	2.0086664659
O	2.7147339538	−0.5976263990	−2.0475973960	H	0.8120453070	0.5827965495	1.2560472459
O	0.9091188700	0.6401762899	−1.6220559090	H	1.5243058729	−2.0423574742	−0.2016695095
C	−2.6771746643	0.8251410655	1.3556642114	H	2.6907834877	−0.9320920692	0.4586085214
O	−3.5330811086	1.0914851642	2.0967311213	H	2.6943359897	−0.0914334137	−2.8807033603

HMDB Wishart Lab (+259.2 kJ/mol)

O	1.2902993462	0.7748644515	−1.7083389606	H	1.4356239625	1.2019876190	−2.5635256549
C	2.3325154891	1.1556150513	−0.7960290002	H	2.0529547848	2.0749435746	−0.2700305885
C	2.5423350305	0.0075874590	0.2186932304	H	3.2737261217	1.3217444571	−1.3281188333
C	1.2632792321	−0.3930754222	0.7708125153	H	2.9831074723	−0.8620023540	−0.2869672684
C	0.1241250724	−0.6642064706	1.1116032657	H	3.2379670804	0.3225504772	1.0049596978
C	−1.2661012990	−0.7595470692	1.0272649393	H	−1.5004715865	−1.3178937887	−1.0332941899
C	−1.8708950565	−0.5112849191	−0.3790788052	H	−3.6802267362	−0.0829447226	−0.9409106723
O	−3.2523867685	−0.5709459017	−0.2208007016	H	−0.5857803557	0.7262364575	−1.2211846645
O	−1.4903807178	0.7440785720	−0.8397769416	H	−1.7423122669	−1.1556018940	2.9650270107
N	−2.0726058158	−1.0040520536	2.0168899709	H	−3.0746729891	−1.0078535234	1.8220056511

3.1.3 MS^2 fragment ion, m/z 116

This work, mzCloud (0.0 kJ/mol)

N	1.7341580424	1.9062281620	0.0963882867	H	1.2389041293	2.6074509263	0.6389523933
C	1.4586600474	0.6404361379	0.2017338300	H	2.3914476561	2.2471613831	−0.5975820407
C	0.5935698757	0.0889996966	1.2783420715	H	2.0569046770	−0.0347811106	−0.4022247030
C	−0.1910031832	−1.1847728883	0.8964706413	H	1.2938300235	−0.1717465010	2.0884999099
C	−1.4139809793	−0.9533971300	0.0009190179	H	−0.0669549029	0.8682679993	1.6775160119
C	−1.0854777101	−0.2803956678	−1.3055402735	H	0.4888498163	−1.9030890850	0.4239663081
O	−2.0573589087	−0.3631336375	−2.2054183734	H	−0.5333808351	−1.6514714961	1.8236892972
O	−0.0295454603	0.3118895519	−1.5268973454	H	−2.1648662107	−0.3309051150	0.5068193143
				H	−1.9164815814	−1.9014161149	−0.2157320739
				H	−1.7971744964	0.1046748895	−3.0202022723

METLIN (+39.6 kJ/mol)

O	3.1190172938	0.9473444291	1.3553825863	H	0.4914799200	1.9639856227	−0.9854470535
C	2.1819050633	0.1922827082	1.2693570622	H	2.0285580569	2.1643140470	−0.1487811164
C	1.0140497801	0.5098626872	0.3189057974	H	0.2272956541	−1.3669710192	−0.0937006431
N	1.1788755501	1.6184511943	−0.3197462443	H	−0.5747150022	−0.5162527047	1.1760208556
C	−0.1649493603	−0.3693930829	0.1633047992	H	−1.6337524499	1.0680460645	−0.5223697683
C	−1.2467441637	0.0854719892	−0.8247651453	H	−0.8098453573	0.1940982123	−1.8267581357
C	−2.4097750401	−0.9137126914	−0.8899251526	H	−2.0681076837	−1.9000977875	−1.2190517590
O	1.9874261043	−0.9494893189	1.9115111252	H	−2.8924588913	−1.0256781175	0.0860340971
				H	−3.1660380832	−0.5686281035	−1.5988250599
				H	2.7375786092	−1.1338341285	2.5088537552

HMDB Wishart Lab (+41.0 kJ/mol)

N	0.5523915578	−0.4763682339	2.6561096182	H	0.1011457479	−0.8029720165	3.4945264457
C	0.9729803962	−0.1571319607	1.6336078281	H	2.4377538818	0.9860365594	0.8173347616
C	1.6105016699	0.3659484343	0.4443857526	H	2.0378060078	−0.4891531495	−0.0926617548
C	0.6607881141	1.2057368212	−0.4676274417	H	1.3229763806	1.8388526597	−1.0645452508
C	−0.2266034204	0.3905070990	−1.4163075530	H	0.0544706789	1.8717296983	0.1515507264
C	−1.1885510936	−0.5726486832	−0.7286622052	H	−0.8183962322	1.0999500636	−2.0029255673
O	−1.8809629444	0.1286588471	0.2872357370	H	0.3864893386	−0.1826729587	−2.1193388861
O	−0.3974021798	−1.6206255404	−0.1635167187	H	−1.8933517822	−0.9953036931	−1.4538634635
				H	−2.7888825386	−0.1904114068	0.3744343004
				H	−0.9430535826	−2.4002325400	0.0101636712

3.1.4 MS^2 fragment ion, m/z 98

This work (0.0 kJ/mol)

O	2.6977554741	0.6367026403	−0.0868159329	H	0.9989419750	2.3717832509	−0.1848523582
C	1.5483205619	0.3183380381	−0.1198742777	H	−1.3179361633	2.2418663229	−0.1583617636
N	0.5602253743	1.4494762653	−0.1264858680	H	−2.2742680254	0.1187663576	0.6504376120
C	−0.7292598815	1.3258060426	−0.0954441367	H	−1.8007423975	−0.2049639261	−0.9923905020
C	−1.3894393655	0.0067554318	0.0116036937	H	−0.3079118191	−1.0106804120	1.5795190757
C	−0.4463018978	−1.1046661040	0.4968700443	H	−0.9057008992	−2.0798061307	0.3223408413
C	0.9186589168	−1.0408710881	−0.2105769491	H	1.6316785750	−1.7614989854	0.1967055297
				H	0.8159795721	−1.2669077031	−1.2826750084

mzCloud, METLIN (+95.3 kJ/mol)

N	2.4618791783	−0.1361318592	−0.7505798229	H	2.9556021216	0.3682357360	−1.4835703635
C	1.1718918314	−0.1026937648	−0.6507665655	H	3.0346666398	−0.6775720608	−0.1055326914
C	0.3963858532	−0.7946039760	0.3851314258	H	0.6420055505	0.4950386053	−1.3930237353
C	−0.6223760033	0.1657927260	1.1144055674	H	−0.1993456451	−1.5601751756	−0.1338501303
C	−1.6002272270	0.8678676181	0.2063606936	H	1.0482805517	−1.2979169784	1.1072990163
C	−2.8168334417	0.3955399077	−0.0389898686	H	−0.0579811205	0.9065509926	1.6880416285
O	−3.8779366820	−0.0268652981	−0.2600086803	H	−1.1372784396	−0.4640447740	1.8446109761
				H	−1.3987331671	1.8610783010	−0.1842274498

HMDB Wishart Lab trans (+138.8 kJ/mol)

O	−1.4586940816	−1.4766723582	0.0161366346	H	−1.9971299210	−2.2643428275	−0.1380920871
C	−1.6789326572	−0.5211527515	−1.0226774782	H	−1.1662159356	−0.8241614415	−1.9469384223
C	−1.1504407384	0.8317624941	−0.5491487399	H	−2.7463262292	−0.4156885316	−1.2462685363
C	0.2872706511	0.9106129928	−0.1692178052	H	−1.3437323096	1.5955151889	−1.3142099150
C	1.0904844357	−0.1564439400	0.0933831514	H	−1.7193942864	1.1656531087	0.3356119624
C	2.4248312338	0.0302440907	0.4591234204	H	0.7028469774	1.9141990595	−0.0810901055
N	3.5360726009	0.1451897276	0.7626037648	H	0.7214965029	−1.1814061362	0.0729448793
				H	4.4979637569	0.2467913242	1.0412392765

HMDB Wishart Lab cis (+148.8 kJ/mol)

O	−2.5262462502	0.6669420812	0.3544778394	H	−3.4248010444	0.4735557189	0.6528303061
C	−1.8391239181	−0.5313866436	0.0319855843	H	−1.6444184272	−1.1372021655	0.9277573001
C	−0.5065571769	−0.1072407850	−0.6203632687	H	−2.3944441715	−1.1442668523	−0.6881224233
C	0.2402241600	0.8598576157	0.2244474846	H	0.0854355051	−0.9882668171	−0.8890948388
C	1.5656517156	0.8546303315	0.5369696711	H	−0.7599364755	0.4262891038	−1.5499938740
C	2.4443902867	−0.1333556647	0.0814692356	H	−0.3521802469	1.6761767916	0.6339771269
N	3.2081158956	−0.9293669865	−0.2692953827	H	2.0229565065	1.6235324479	1.1557482894
				H	3.8810336411	−1.6097981756	−0.5826930503

3.2 Urocanic acid

3.2.1 Precursor ion, m/z 139

Protonated urocanic acid (0.0 kJ/mol)

O	3.5606912580	1.8947463799	−0.2268382922	H	4.5244925875	1.7909019177	−0.1336538240
C	2.9749139909	0.7116001020	0.0289176315	H	1.0778818369	1.7787819999	−0.3707637754
C	1.4913245940	0.8121288169	−0.0996545187	H	1.2661902169	−1.1997027334	0.3889460340
C	0.7454435309	−0.2812182528	0.1224339433	H	−1.5999784777	1.6289266409	−0.5197841974
C	−0.7012814543	−0.3425771219	0.0370110447	H	−3.7953588788	0.3625753137	−0.3553289965
C	−1.6750916701	0.5844299923	−0.2632043825	H	−3.4802057649	−2.0890248095	0.2811525740
N	−2.8909786083	−0.0636243128	−0.1854829163	H	−0.9616677127	−2.4107506934	0.5372732886
C	−2.7095720173	−1.3449160183	0.1483283115				
N	−1.3937689565	−1.5279746803	0.2858907886				
O	3.5671655255	−0.3044025409	0.3248572869				

Protonated urocanic acid (+0.7 kJ/mol)

O	3.8585263790	−0.6938740470	0.7677223200	H	4.7714885055	−0.3623184070	0.6976201434
C	3.0298203050	0.2130932212	0.2179226066	H	1.4403052100	−1.2104918979	0.7697758718
C	1.6120525390	−0.2463682056	0.2986422861	H	0.9291903343	1.4814865152	−0.6523341037
C	0.6414604167	0.5340923085	−0.2021832832	H	−1.8121126647	1.9585036817	−1.1896288942
C	−0.7770760924	0.2429067121	−0.2070036811	H	−3.9144863233	0.5924953791	−0.7222318646
C	−1.8168374517	0.9964340907	−0.7015797065	H	−3.4082264711	−1.6150744676	0.4533020467
N	−2.9803769685	0.2903143701	−0.4682352615	H	−0.8835238181	−1.6751358298	0.7555508801
C	−2.6982062480	−0.8614943311	0.1470451193				
N	−1.3734831005	−0.9065842143	0.3125313214				
O	3.3813854488	1.2618151221	−0.2768158005				

3.2.2 MS^2 fragment ion, m/z 121

This work (0.0 kJ/mol)

O	−1.3846206476	2.4743265697	0.1759334053	H	−3.2260503112	0.2654182711	0.0810367063
C	−1.2364671590	1.2948912539	0.1024120476	H	−1.8801331675	−2.0255557106	−0.0831446665
C	−2.1533254100	0.1315937586	0.0513679679	H	1.5843584070	−2.2816210987	−0.1684385872
C	−1.4643234017	−1.0277597492	−0.0318757048	H	3.1123838775	−0.2358425713	−0.0769468849
C	−0.0289913616	−0.7345798044	−0.0433355623	H	1.8212499521	1.9694228000	0.0809921171
C	1.2268819406	−1.2658997618	−0.1004770696				
N	2.0997242238	−0.1756505013	−0.0528997786				
C	1.4089759764	0.9714167506	0.0296384629				
N	0.1203370809	0.6398397933	0.0358375471				

HMDB MoNA cis (+33.6 kJ/mol)

O	−2.1269479451	1.5952893398	−2.1249206286	H	−3.3554822282	−0.2257174401	−0.0084614443
C	−2.1030364415	0.8722959129	−1.2413616057	H	−1.5679985436	−1.2253423772	1.3614881391
C	−2.3082910986	−0.0000569009	−0.1950705575	H	1.3235068578	−1.4254732715	1.8499893340
C	−1.2856086731	−0.5485113592	0.5601125441	H	3.2356532329	−0.2903632957	0.6275964468
C	0.0791093311	−0.2743174559	0.3411737433	H	2.4121533626	1.2128542832	−1.2749048441
C	1.2102218599	−0.7486400408	1.0151500097				
N	2.2576621769	−0.1645537148	0.3915186582				
C	1.7651514796	0.6371037327	−0.6275908555				
N	0.4637066291	0.5856325874	−0.6748189396				

HMDB MoNA trans, mzCloud, METLIN (+46.0 kJ/mol)

O	4.5080166496	0.3745753600	0.2313140963	H	1.6661491114	1.0935914401	1.3671759326
C	3.3713945468	0.4194253975	0.3595734960	H	1.5382330381	−0.7760221784	−1.1365813081
C	2.0229669043	0.4894041155	0.5342405090	H	−1.1557521052	−1.3906902458	−1.8010914344
C	1.1468749228	−0.1815937789	−0.3146869701	H	−3.3212273981	−0.6700489319	−0.7035177832
C	−0.2456091792	−0.1191470296	−0.1475017789	H	−2.9186436832	0.8539243939	1.3195100911
C	−1.2288056577	−0.7487018955	−0.9343524393				
N	−2.4009954692	−0.3898541851	−0.3821921168				
C	−2.1298928554	0.4350592362	0.7096725489				
N	−0.8527088254	0.6102783074	0.8686371530				

METLIN (+226.5 kJ/mol)

O	2.3714301563	−1.6883547914	1.4157628286	H	3.7355387985	−0.2226500222	1.7642849802
C	2.8211109696	−0.5875472383	1.2667551850	H	2.8227790633	1.2634412326	−0.0370042664
C	2.1751747707	0.4708979059	0.3404450555	H	−0.5901447962	−1.0311113999	−1.9913061254
C	0.9176259024	0.4418703904	0.0579538224	H	−3.0610455655	−0.6807138251	−1.5912969888
C	−0.4062617482	0.3386963223	−0.1648689294	H	−3.5134300049	0.8534531144	0.4284358918
C	−1.0239180918	−0.4258165986	−1.2062503197				
N	−2.3262378820	−0.2641350877	−1.0253905069				
C	−2.5206156523	0.5816405123	0.0945736322				
N	−1.4021059198	0.9505294853	0.6182057410				

METLIN (+349.3 kJ/mol)

O	3.2704838807	1.8325687821	−1.0699139466	H	2.7684112884	2.7887953092	0.7536639305
C	2.6020555344	2.0608998005	−0.0431627437	H	1.2323422740	−0.3180744169	−1.7571266123
C	1.6517874138	1.1010232478	−0.3060602878	H	−1.1539641657	−1.9997855822	−1.4402016877
C	0.9055228137	0.1231386242	−0.8130901236	H	−2.8908136668	−2.0161779331	0.4100933551
C	−0.2703641983	−0.3400251402	−0.1666682740	H	−2.3973567950	−0.2719199701	2.2175083230
C	−1.1470638822	−1.3503949823	−0.5766899405				
N	−2.0929107321	−1.3912831779	0.3880679480				
C	−1.7839523018	−0.4319079933	1.3417043189				
N	−0.6940774628	0.2131434321	1.0319757407				

HMDB Wishart Lab (+496.4 kJ/mol)

O	3.5679373848	0.3166437103	0.0508039380	H	3.1476826889	1.0569114081	0.5385324618
C	2.5695093229	−0.7606887101	−0.3702277206	H	4.1249736486	0.6590244359	−0.6803419659
C	1.3042290792	−0.4934302898	−0.2197360617	H	3.1346990775	−1.6308696074	−0.6689671601
C	0.0693391710	−0.2391356932	−0.0702028179	H	−2.3489546374	−1.8997366374	−0.5458480909
C	−1.2213446607	0.0292516053	0.0876135163	H	−3.7394253174	1.7941278127	0.8418116990
C	−2.3877421873	−0.8801404713	−0.1801561357				
N	−3.4691157771	−0.2419199907	0.1010666045				
C	−3.0194162645	1.0430300859	0.5381036362				
N	−1.7326715286	1.2470323419	0.5477480970				

3.3 Citrulline

3.3.1 Precursor ion, m/z 176

Protonated citrulline (0.0 kJ/mol)

N	0.3495478108	1.5282231889	−1.0754326455	H	0.2501159431	2.5263244040	−1.2713591386
C	1.3362807500	1.2818032298	0.0371000940	H	0.7269574389	1.0927386925	−1.9310915082
C	0.8279441709	0.2830688062	1.1042989477	H	1.5233672589	2.2373772635	0.5343447389
C	0.7127036088	−1.2020945722	0.7112791026	H	−0.1256485758	0.6568912349	1.4921338388
C	−0.4472835473	−1.6166957542	−0.2135173356	H	1.5408836397	0.3595668426	1.9309014032
N	−1.7812312689	−1.3750116799	0.3386264854	H	1.6463788124	−1.5473728558	0.2490972974
C	−2.5191723139	−0.2686006873	0.0565543806	H	0.6179978182	−1.7699076656	1.6437567669
N	−3.7978028515	−0.2289349785	0.5014394153	H	−0.3928348013	−1.1321230647	−1.1920356269
O	−2.0438029079	0.7096393299	−0.5746384705	H	−0.3658180301	−2.6902532098	−0.4055400424
C	2.6337906007	0.8247592932	−0.6342192579	H	−2.2110068419	−2.1220313102	0.8663420425
O	2.6954109073	0.5394475873	−1.8129479598	H	−4.2811243410	−1.0380912740	0.8605707378
O	3.6455181165	0.7693418266	0.2294038724	H	−4.3486544158	0.5781498860	0.2488229251
				H	−0.6442431523	1.1311582782	−0.8568212145
				H	4.4518261715	0.4726271886	−0.2314688489

3.3.2 MS^2 fragment ion, m/z 159

This work (0.0 kJ/mol)

O	−2.0764578161	2.0959819032	−0.6345323040	H	−2.7325411003	−0.1215647618	−0.4400837629
C	−1.2867763202	1.1674340611	−0.1116924902	H	−2.1530090038	−1.8888528463	0.8314389577
N	−1.8417303533	−0.0182844847	0.0343479579	H	−1.4476207477	−0.7317337511	1.9649561376
C	−1.4014176532	−1.1051364093	0.9351163561	H	0.0349509799	−1.9779676828	−0.4255396645
C	−0.0095891833	−1.6555569492	0.6217451754	H	0.1232240105	−2.5566500764	1.2295225959
C	1.1248273446	−0.6815152101	0.9534774085	H	2.0891005037	−1.1693341522	0.7881295562
C	1.1344872377	0.6203054090	0.1296882954	H	1.0829314302	−0.4033362326	2.0124939032
C	1.3195733793	0.3872325259	−1.3825388024	H	1.9849798732	1.2177636527	0.4713814251
O	0.5266538625	0.7583471568	−2.2215444033	H	2.5619996102	−0.3750728076	−2.5972373388
O	2.4595832891	−0.2606142883	−1.6346202984	H	0.1448224865	2.4737356152	0.3251091110
N	−0.0507612765	1.4792265366	0.3168615557	H	−1.5872305532	2.7456927919	−1.1667793711

METLIN (+0.2 kJ/mol)

N	−2.8958044639	−0.7690734177	1.4540217865	H	−2.1543149558	−0.6305704750	2.1271570100
C	−2.7470466718	−0.5676118526	0.1350430259	H	−3.7953252363	−1.0947403448	1.7834527774
N	−1.3771771837	−0.0883214597	−0.1902719634	H	−0.6608703457	0.0159933132	0.5751644396
C	−1.0365569213	0.1098844887	−1.4161339570	H	−1.8136859994	−0.0962490239	−2.1558028718
C	0.2872936137	0.6009167859	−1.8698946068	H	0.7037546556	−0.1925531973	−2.5127904746
C	1.3287715090	1.1184393823	−0.8567267331	H	0.0513864318	1.4016121306	−2.5878782600
C	2.1802068301	0.0851685713	−0.0850275785	H	0.8567939003	1.8186751670	−0.1583261257
C	1.6777663751	−0.3001311556	1.2879004295	H	2.0260002868	1.7213513924	−1.4445001285
O	2.6344983203	−0.7973460442	2.0578683091	H	3.1953423850	0.4693858671	0.0513738577
O	0.5154753160	−0.1860793909	1.6817897501	H	2.3047200834	−0.8470092179	−0.6535717926
O	−3.5497886283	−0.7201553918	−0.7519665261	H	2.2685606991	−1.0515861273	2.9249196323

mzCloud (+4.7 kJ/mol)

O	1.8322045542	−0.7325175062	0.6519880658	H	3.8514583525	−0.9286653986	−0.4730213952
C	1.9877435387	−0.5995248702	−0.5608895897	H	0.7759198934	−1.2074373850	−2.1830332799
O	3.1736933245	−0.7154124586	−1.1413739926	H	1.2458322874	0.4668794162	−2.2429412916
C	0.8851673482	−0.3104033881	−1.5597690983	H	−1.1555740836	0.2101980111	−1.8126215248
C	−0.4779789099	0.0943274346	−0.9614786473	H	0.6535828009	1.6800113904	0.0541373877
C	−0.3836856387	1.4699529236	−0.2300762545	H	−0.7035216140	2.2715390392	−0.8972573737
N	−1.2365435846	1.5402773280	0.9654141679	H	−1.6068125551	2.4403593681	1.2508375612
C	−1.3317223965	0.5746591032	1.8946705323	H	−1.0942241574	−1.4578570134	2.0098161026
O	−1.8384382373	0.5825692042	2.9849549729	H	0.3068559610	−0.7243968846	1.3909666766
N	−0.7481725476	−0.7320530621	1.3737546638	H	−2.2145829763	−0.9478495988	−0.1208872577
C	−1.1251711809	−0.9831630212	−0.0663075872	H	−0.7958301791	−1.9913926316	−0.3268828384

This work (+21.8 kJ/mol)

N	−1.1810846065	1.2247332534	−0.2811514282	H	−0.1428091883	1.4207125969	−0.1860603036
C	−1.4341880984	−0.2658177477	−0.3321774791	H	−1.5292847255	1.7057890276	−1.1128894962
C	−0.2414368801	−1.0719257272	−0.8790347975	H	−2.2962442551	−0.4241318277	−0.9890253565
C	1.0566440839	−1.0848574536	−0.0363978099	H	−0.0334772364	−0.7343185402	−1.9020847326
C	2.0966410848	−0.0152200720	−0.3767442201	H	−0.6128572668	−2.0961781906	−0.9735665861
N	1.5909994330	1.3509160671	−0.0863458963	H	0.8316707870	−1.0502067499	1.0365155449
C	2.2512099713	2.3544348819	0.1824569252	H	1.5485983010	−2.0484573511	−0.2051910044
O	2.7712659701	3.3630360990	0.4484423862	H	3.0060245960	−0.1966406046	0.2020373308
C	−1.8814642545	−0.6451676578	1.0865507247	H	2.3553919087	−0.0614240643	−1.4403127578
O	−2.0813837580	0.1904395446	1.9439413969	H	−2.3737982383	−2.1564384554	2.1180453638
O	−2.0446620582	−1.9564377845	1.2218552531	H	−1.6556555697	1.5931607561	0.5609369428

HMDB MoNA cis (+36.7 kJ/mol)

O	−0.3374342971	2.2344005063	−2.6964323276	H	0.4159847635	2.1162819942	−3.3050466000
C	−0.3496773334	1.2695992339	−1.7928805854	H	−2.1482999558	0.4722595113	−1.0320124006
C	−1.5229631853	1.3516557953	−0.8296213718	H	−2.1172965457	2.2348747921	−1.0690928234
C	−1.0762092815	1.3889140960	0.6179943063	H	−0.9568724284	2.3836240626	1.0408300111
C	−0.8146626087	0.3390978554	1.4096777565	H	−0.5025015972	0.5280757484	2.4330823655
C	−0.9157341873	−1.1099594202	1.0099442612	H	−0.9428200437	−1.7679858944	1.8785675372
N	0.2993366478	−1.5332454537	0.1858841822	H	−1.7830738864	−1.3178264436	0.3772515020
C	1.6027140562	−1.5046367923	0.9998528993	H	0.1743557077	−2.5028427240	−0.1269113199
N	2.5777644918	−0.8533293755	0.3533150514	H	0.3608959590	−0.9032057071	−0.6526902958
O	1.6139643421	−2.0615398128	2.0639640009	H	2.4293976054	−0.3504023361	−0.5123955149
O	0.5088955245	0.3850884652	−1.7580788943	H	3.4845362524	−0.7989981014	0.8006982604

HMDB MoNA trans (+54.8 kJ/mol)

N	−1.9004769724	0.9609753777	−0.8275127594	H	−2.5148586201	1.7606092139	−0.7263326482
C	−2.4672197639	−0.2369103621	−0.6913343972	H	−0.8914427387	1.1362701128	−0.8814720680
N	−1.4521489238	−1.4031881337	−0.8810979860	H	−2.0178432287	−2.1567844895	−1.2890959298
C	−0.7934099288	−1.9048837842	0.4240653570	H	−0.7179321384	−1.1539908719	−1.5530658379
C	0.0665981882	−0.8173859803	0.9916717330	H	−0.2215971150	−2.7909230636	0.1375497079
C	1.3242015448	−0.6149416412	0.5835317454	H	−1.6186128177	−2.1994427438	1.0746708964
C	2.1215422203	0.6119045829	0.9211533520	H	−0.4033991499	−0.1293386557	1.6909781989
C	1.8794966255	1.7034722321	−0.1172671768	H	1.7901621599	−1.3199193228	−0.1068084070
O	2.8595711054	2.6027588941	−0.1350141597	H	1.8194549467	1.0309005439	1.8897415709
O	0.8943321691	1.7725960037	−0.8400831599	H	3.1945968350	0.4126326961	0.9819028040
O	−3.6008714246	−0.5661079137	−0.4687958762	H	2.6497570272	3.3015973055	−0.7813849595

3.3.3 MS^2 fragment ion, m/z 158

This work (0.0 kJ/mol)

N	2.9338369118	1.1106944769	0.9508646619	H	3.9105478570	1.1680096510	1.2029176498
C	2.5328397514	−0.0454879418	0.3934083351	H	2.2895103227	1.8518747555	1.1890303681
O	3.2370297678	−1.0044725915	0.1537946277	H	1.5519250717	−1.7587074828	−1.2299742480
N	1.1037053257	−0.1421620079	0.0189252171	H	0.7833429781	−2.1978012665	0.2833479764
C	0.7510100602	−1.4836764407	−0.5457729721	H	−0.8607309025	−2.5668474742	−1.4276980696
C	−0.6046716032	−1.5200431474	−1.2434124253	H	−0.5457020838	−1.0343533381	−2.2250904720
C	−1.6954332507	−0.8475550400	−0.3999277653	H	−1.7736909025	−1.3349600287	0.5814305974
C	−1.2563443509	0.5988693872	−0.2462947134	H	−2.6726518742	−0.9124824651	−0.8889484491
N	−2.1141552878	1.4365116042	0.6816719841	H	−1.2903335491	1.1093411986	−1.2153151872
C	0.1807368647	0.7968161377	0.3001214172	H	−2.9016689419	1.8853200060	0.2078185399
O	0.3648698554	1.8506776484	0.9340351023	H	−1.4444179967	2.1509657974	1.0569175214
				H	−2.4791540230	0.8894685618	1.4680503036

This work (+0.9 kJ/mol)

N	2.9060807485	0.9240667002	1.1118503030	H	3.8643183514	0.9247317766	1.4319125652
C	2.5236304012	−0.1278614002	0.3670845106	H	2.2746747241	1.6720530245	1.3646785494
O	3.2171460890	−1.0688539348	0.0421146586	H	0.5605085728	−0.8850700506	−1.9716108838
N	1.1202822036	−0.1106482193	−0.0897012751	H	1.5096402368	−1.9574973648	−0.9467109184
C	0.6863784062	−1.2450020245	−0.9438099897	H	−0.9536423970	−2.6142403041	−1.1256652636
C	−0.6001022017	−1.8789785049	−0.3984184884	H	−0.3623836179	−2.4257270905	0.5177800038
C	−1.7123495294	−0.8317833263	−0.1039299239	H	−2.0025448449	−0.8856753585	0.9522899544
C	−1.1899282954	0.5822898068	−0.4021994948	H	−2.6106295965	−1.0229953828	−0.6983475696
N	−2.0452942751	1.6809109104	0.1923366073	H	−1.1430908356	0.7592036072	−1.4808193678
C	0.2080627335	0.8300205303	0.2072324226	H	−2.7411492944	1.3211448081	0.8519967559
O	0.3561852060	1.8434908347	0.9113795477	H	−1.3386912957	2.2669161545	0.7182086302
				H	−2.5271014896	2.2496048082	−0.5076513335

mzCloud (+97.3 kJ/mol)

O	1.0258280029	−1.3625481155	0.9038731234	H	3.9967765854	−0.8091059840	−0.0805850856
C	1.9308188048	−0.8813495779	0.1659922536	H	3.4160676323	−1.5226914331	1.3842396199
N	3.2249969623	−1.0059685157	0.5384767751	H	2.4304065118	0.1652245212	−1.5089654963
N	1.6568396152	−0.2214100519	−0.9852619063	H	0.4833349957	−0.3082346258	−2.6920853268
C	0.3334969321	−0.1795775893	−1.6162525793	H	−0.2283594416	−1.0548862328	−1.2780608832
C	−0.4457474924	1.1203284108	−1.3729639540	H	0.1664998980	1.9689487454	−1.7014732433
C	−0.8959647739	1.3944710636	0.0772221755	H	−1.3281741995	1.1048394593	−2.0236993481
C	−1.8824162184	0.3973318751	0.6662963581	H	−1.3489994236	2.3901643711	0.1130678499
N	−1.3726687470	−0.7539726001	1.4348186646	H	−0.0294256627	1.4502939386	0.7487454132
C	−3.1982998910	0.5584496578	0.5775580261	H	−0.3191602850	−0.9916980836	1.1240892699
O	−4.3468245218	0.7176217372	0.4705996327	H	−1.3475950592	−0.5701050056	2.4440666129
				H	−1.9213302244	−1.6062259649	1.2931020480

3.3.4 MS^3 fragment ion, m/z 141 (via fragment ion m/z 159)

No matching structures found.

3.3.5 MS^2 fragment ion, m/z 116

MS^2 fragment ion, m/z 116 (structure I)

This work (0.0 kJ/mol)

O	1.4382851459	0.6415679065	1.8784875366	H	3.0597316921	1.8209662805	0.5928773405
C	1.6337159768	0.5732941187	0.6806043902	H	1.4696233494	−1.0566910322	−0.7188287678
O	2.5443464721	1.2479566893	−0.0054613405	H	−0.2645445600	−0.2146743348	−2.1069402705
C	0.8049873866	−0.3454368091	−0.2233420738	H	0.3031621469	1.3498115467	−1.5211611021
C	−0.1278784771	0.3966177154	−1.2102435520	H	−2.3040839648	0.7067579356	−1.1033022457
C	−1.4519004318	0.5431794035	−0.4403031877	H	−1.4123686845	1.3801744937	0.2648356055
C	−1.5770210842	−0.7838808472	0.3054029820	H	−2.1986376350	−0.7637069135	1.2013204790
N	−0.1371020258	−1.0917371376	0.7165132022	H	−1.9051162979	−1.5941802470	−0.3489682904
				H	0.0505639797	−2.0966433209	0.7377275947
				H	0.0742370114	−0.7133754476	1.6608816999

This work (+1.4 kJ/mol)

O	1.7176317382	0.5866660313	1.8701690109	H	3.3360034110	1.5932414544	0.4367223691
C	1.7849018184	0.5238269673	0.6587141173	H	1.2517006033	−1.0120794899	−0.7646507445
O	2.6990609782	1.0968589354	−0.1110116073	H	0.1960964195	0.7124054597	−2.0506640023
C	0.7547598918	−0.2447480138	−0.1660121174	H	−0.2444815477	1.6439582853	−0.6154843438
C	−0.1815796107	0.6326503807	−1.0302029541	H	−2.3765782506	0.5806968739	−1.1876769897
C	−1.5422657601	−0.0773570922	−0.9346689564	H	−1.5838534273	−0.9420239518	−1.6061316206
C	−1.6079432298	−0.5220378245	0.5256672372	H	−1.8507805373	0.3038659664	1.1973472099
N	−0.1675444160	−0.9344330694	0.8356969562	H	−2.2691173176	−1.3638560455	0.7334442848
				H	−0.0583100407	−1.9501231000	0.7928259395
				H	0.1423992773	−0.6273117674	1.7760162112

METLIN (+13.5 kJ/mol)

N	2.3518469075	1.0239573450	−0.2391187060	H	2.7791843710	0.7739754269	0.6475928653
C	1.3698072872	0.3370933383	−0.7421517912	H	2.6670373001	1.8871017281	−0.6698120982
C	0.9557096103	−0.9885672284	−0.2104604083	H	0.9936987863	0.6683581846	−1.7051610406
C	−0.5413385959	−1.3230723432	−0.3860477942	H	1.2775275257	−1.1021122346	0.8318603448
C	−1.4872123669	−0.6112482306	0.5885553436	H	1.5389645164	−1.7159376336	−0.7982142158
C	−1.4033696629	0.8907766361	0.5209301377	H	−0.6571521414	−2.4007208105	−0.2445367855
O	−2.4196237716	1.5071653258	1.1112450424	H	−0.8420540903	−1.1149821838	−1.4193470181
O	−0.4773397830	1.5040702129	−0.0092898561	H	−2.5244215683	−0.9079272903	0.4031821996
				H	−1.2790722751	−0.9014230377	1.6276589191
				H	−2.3020920493	2.4731927949	1.0530148616

MS^2 fragment ion, m/z 116 (structure II)

METLIN (+13.5 kJ/mol)

N	2.3518469075	1.0239573450	−0.2391187060	H	2.7791843710	0.7739754269	0.6475928653
C	1.3698072872	0.3370933383	−0.7421517912	H	2.6670373001	1.8871017281	−0.6698120982
C	0.9557096103	−0.9885672284	−0.2104604083	H	0.9936987863	0.6683581846	−1.7051610406
C	−0.5413385959	−1.3230723432	−0.3860477942	H	1.2775275257	−1.1021122346	0.8318603448
C	−1.4872123669	−0.6112482306	0.5885553436	H	1.5389645164	−1.7159376336	−0.7982142158
C	−1.4033696629	0.8907766361	0.5209301377	H	−0.6571521414	−2.4007208105	−0.2445367855
O	−2.4196237716	1.5071653258	1.1112450424	H	−0.8420540903	−1.1149821838	−1.4193470181
O	−0.4773397830	1.5040702129	−0.0092898561	H	−2.5244215683	−0.9079272903	0.4031821996
				H	−1.2790722751	−0.9014230377	1.6276589191
				H	−2.3020920493	2.4731927949	1.0530148616

This work (+71.2 kJ/mol)

O	2.8577693275	0.0457827932	0.4001856447	H	3.6798209324	−0.4689938285	0.4573333495
C	1.8248090614	−0.6823313038	0.0441956370	H	1.9272708630	−1.7496423937	−0.1471894448
N	0.6649883795	−0.1342078032	−0.0654860844	H	0.5296506734	1.4886262716	1.2622541010
C	0.3427117333	1.2934826433	0.2015849110	H	0.9961139720	1.9387732467	−0.3895028352
C	−1.1494220821	1.3925382546	−0.1668293172	H	−1.2625113778	1.6908636650	−1.2135479871
C	−1.6956015615	−0.0289626317	0.0491271130	H	−1.6615883291	2.1359697037	0.4460170616
C	−0.5811206764	−0.9361590403	−0.4657264055	H	−1.8604091078	−0.2242691919	1.1174389896
O	−0.5038908715	−2.2367468084	−0.0035897629	H	−2.6317221841	−0.2176859221	−0.4798570873
				H	−0.5660152664	−0.9848080727	−1.5588929164
				H	−0.9108534858	−2.3223295819	0.8723850335

This work (+75.0 kJ/mol)

O	2.8861045860	0.1406558853	0.2735617134	H	3.7236589320	−0.3337068988	0.4076899901
C	1.8661957564	−0.6634694132	0.0911732951	H	2.0064081911	−1.7435054643	0.0984996992
N	0.6855210516	−0.1769019059	−0.0834711235	H	0.9017061795	1.7770237803	0.7174216371
C	0.3276275978	1.2682517032	−0.0590463752	H	0.5900800514	1.7006232797	−1.0303422707
C	−1.1882756882	1.2378515347	0.1746586112	H	−1.6733252484	2.1283656550	−0.2287848195
C	−1.6417278359	−0.0529799657	−0.5331657824	H	−1.4056683410	1.2042589582	1.2477467554
C	−0.5398750227	−1.0825046706	−0.2520478260	H	−2.6112936036	−0.4279207640	−0.1972811925
O	−0.6896343396	−1.8898149886	0.8679331787	H	−1.7105759764	0.1102049331	−1.6137339632
				H	−0.3379006535	−1.7527376515	−1.0895682918
				H	−1.1891256364	−1.4438940068	1.5687567647

This work (+76.8 kJ/mol)

O	3.2596880514	−0.0905577792	−0.0909044515	H	4.1498463858	−0.3203412687	0.2223791881
C	2.3156165419	−0.4220464628	0.7577252342	H	2.5726797734	−0.8841161645	1.7096471313
N	1.0793305264	−0.2047931255	0.4732444353	H	1.2578278823	0.1475458804	−1.5832175613
C	0.5637096080	0.3962423384	−0.7780773557	H	0.5699363703	1.4856062717	−0.6545959101
C	−0.8430383823	−0.1257711420	−1.1040279875	H	−0.8344785105	−1.2219590299	−1.0954707834
C	−2.0155760823	0.3974213175	−0.2518656240	H	−1.0416090015	0.1661758867	−2.1401124243
C	−2.1011093817	−0.0441509712	1.1849225720	H	−2.0421632126	1.4996285863	−0.2368074959
O	−1.1847383354	−0.5435296651	1.8275387758	H	−2.9640404011	0.1125119619	−0.7265344585
				H	−3.0726928918	0.1169531079	1.6831234401
				H	0.3308110594	−0.4647197421	1.1629332754

3.3.6 MS^3 fragment ion, m/z 115 (via fragment ion m/z 159)

This work (0.0 kJ/mol)

N	2.3432178570	−0.0293148693	0.2853270013	H	2.9177720632	−0.7631261762	0.6869468718
C	0.9125890138	−0.3200006798	0.3620400353	H	2.6635564580	0.1476745384	−0.6642673961
C	0.1961681895	−0.9525107649	−0.8399602037	H	0.7547706357	−0.9648420575	1.2382125346
C	−1.3084163337	−1.0219309724	−0.5297724181	H	0.5973240788	−1.9537986587	−1.0177252229
C	−1.9204746772	0.3628697655	−0.2927564962	H	0.3837237256	−0.3589030422	−1.7442422155
N	−1.0185462646	1.2408827083	0.4970975284	H	−1.8489458210	−1.4930731400	−1.3551467064
C	0.2423208972	1.0006755736	0.7260761524	H	−1.4704457666	−1.6535983749	0.3514608594
O	0.9858057651	1.9043280431	1.2849215207	H	−2.8667449370	0.2913095533	0.2484380659
				H	−2.1093404225	0.8824453877	−1.2383222576
				H	1.9164023532	1.5269837972	1.2232002840
				H	−1.3710368144	2.1539293690	0.7786720629

mzCloud (+51.5 kJ/mol)

N	2.8234120652	−0.9790830294	0.3177221221	H	2.9980248377	−1.9716207114	0.4098647572
C	1.5926771105	−0.5733077327	0.0132950831	H	3.6075121804	−0.3454825532	0.3795244832
N	1.2494922517	0.7134957070	0.0152909539	H	1.9273339115	1.3612341621	0.3975913751
C	0.0716179448	1.3176391289	−0.6647929819	H	0.3245153434	2.3733760048	−0.7759234413
C	−1.2615592647	1.1718838157	0.0934821599	H	0.0029758274	0.8942043001	−1.6724085736
C	−1.9131055855	−0.1786301814	−0.0765381394	H	−1.1134888355	1.4076559019	1.1528764355
C	−2.1459614083	−1.0562281781	0.9115757135	H	−1.9367745774	1.9359255766	−0.3124840831
O	0.7491165782	−1.5256937279	−0.2973779511	H	−2.2481093782	−0.4217550769	−1.0866190776
				H	−2.6514651912	−1.9990067524	0.7235850709
				H	−1.8722011489	−0.8436984366	1.9431440129
				H	−0.2043126607	−1.2808082170	−0.1423079193

mzCloud (+83.1 kJ/mol)

N	2.6940826995	−0.1415735732	0.1553320921	H	2.7317931376	0.5293636362	−0.5990564993
C	1.6586720798	−0.9550259788	0.4000228442	H	3.5015389940	−0.2161348007	0.7634992432
N	0.5220205826	−0.7763716934	−0.6509657360	H	0.4090071805	0.2209764158	−0.9067835631
C	−0.8375500745	−1.2530320714	−0.1543019784	H	0.7936974081	−1.2940050297	−1.4941262201
C	−1.4816826779	−0.1527873412	0.6990727693	H	−1.4235349707	−1.4691640482	−1.0506491555
C	−1.6050540778	1.1411446096	−0.0669566092	H	−0.6610640298	−2.1722318653	0.4045245633
C	−0.8907104374	2.2446022614	0.1934677779	H	−2.4693271927	−0.5232949039	0.9970729340
O	1.5078714681	−1.7731378652	1.2600635634	H	−0.9059372700	−0.0108534527	1.6204258061
				H	−2.3231818363	1.1529300709	−0.8876115591
				H	−0.2008643187	2.2958887937	1.0343103128
				H	−1.0195766643	3.1528068357	−0.3875405857

mzCloud (+100.4 kJ/mol)

N	−2.6965416265	−0.6394825401	1.0871962476	H	−2.2413864068	−0.4044261586	1.9759711323
C	−2.0434610357	0.0608785799	−0.2053515004	H	−2.7172614775	−1.6605600364	0.9844768749
N	−0.7223987515	−0.0016914016	−0.1705465977	H	−3.6663802409	−0.3036475946	1.1118301830
C	0.1213588301	0.5415700364	−1.2626123134	H	−0.2186675853	−0.4922782705	0.5660308868
C	1.5122092720	0.8818297643	−0.7026609560	H	0.1856942428	−0.2099957824	−2.0567614768
C	2.1630971711	−0.3047277887	−0.0390774212	H	−0.3772829438	1.4256331390	−1.6643094073
C	2.4268596429	−0.3896746628	1.2711293585	H	1.4312856542	1.7177488250	0.0011906358
O	−2.8329128987	0.5206427420	−0.9768464857	H	2.1203828654	1.2264916887	−1.5473078673
				H	2.4225615341	−1.1408378805	−0.6896495094
				H	2.2253964711	0.4375366494	1.9495041365
				H	2.9074472828	−1.2651093086	1.6974940797

3.3.7 MS^3 fragment ion, m/z 115 (via fragment ion m/z 158)

mzCloud (+51.5 kJ/mol)

N	2.8234120652	−0.9790830294	0.3177221221	H	2.9980248377	−1.9716207114	0.4098647572
C	1.5926771105	−0.5733077327	0.0132950831	H	3.6075121804	−0.3454825532	0.3795244832
N	1.2494922517	0.7134957070	0.0152909539	H	1.9273339115	1.3612341621	0.3975913751
C	0.0716179448	1.3176391289	−0.6647929819	H	0.3245153434	2.3733760048	−0.7759234413
C	−1.2615592647	1.1718838157	0.0934821599	H	0.0029758274	0.8942043001	−1.6724085736
C	−1.9131055855	−0.1786301814	−0.0765381394	H	−1.1134888355	1.4076559019	1.1528764355
C	−2.1459614083	−1.0562281781	0.9115757135	H	−1.9367745774	1.9359255766	−0.3124840831
O	0.7491165782	−1.5256937279	−0.2973779511	H	−2.2481093782	−0.4217550769	−1.0866190776
				H	−2.6514651912	−1.9990067524	0.7235850709
				H	−1.8722011489	−0.8436984366	1.9431440129
				H	−0.2043126607	−1.2808082170	−0.1423079193

This work (+60.0kJ/mol)

N	−2.1799886968	0.0729784504	0.3703450294	H	−2.6396315826	0.8196591223	−0.1689957129
C	−0.8074210646	−0.2170974813	−0.2445786101	H	−2.7767298214	−0.7603754395	0.3797390630
C	0.0349582018	−1.1366259948	0.6373401304	H	−1.0308213906	−0.6792497450	−1.2126070268
C	1.4775450373	−1.0987037335	0.1035489359	H	−0.3646465053	−2.1558640910	0.6294993391
C	2.0376593469	0.3315877791	0.1325100145	H	0.0178781927	−0.7757470106	1.6754368908
N	1.1120234274	1.3668001483	−0.3336757059	H	2.1100984856	−1.7585583421	0.7040359853
C	−0.1122951820	1.1162731766	−0.4744613280	H	1.4977699288	−1.4895193491	−0.9214455657
O	−1.0175397317	2.0530278594	−0.8847365065	H	2.3392312315	0.6149566258	1.1490012247
				H	2.9399417673	0.4026170426	−0.4813125618
				H	−0.5492040234	2.8871356174	−1.0664511803

This work (+65.5 kJ/mol)

N	−1.9616054222	0.0307556861	0.6335424349	H	−2.6291566395	−0.7471406506	0.6511744855
C	−0.8599486403	−0.1824538119	−0.4227604038	H	−2.4672679431	0.9003192907	0.4152030261
C	0.0750041715	−1.3408605780	−0.0607294909	H	−1.4149577753	−0.3741596587	−1.3458468692
C	1.2797737489	−0.8513121133	0.7544827203	H	−0.4760516915	−2.1397264776	0.4504377610
C	1.9956342955	0.2854254829	0.0162465178	H	0.4238539587	−1.7736070118	−1.0051906082
N	1.0999059359	1.3660642099	−0.4044723527	H	0.9642799242	−0.4989575026	1.7473525830
C	−0.1311037264	1.1439777408	−0.5519025766	H	1.9705078118	−1.6812803794	0.9280770863
O	−1.0291573733	2.1227888630	−0.8608258125	H	2.5106423518	−0.0890743574	−0.8782127429
				H	2.7668454908	0.7337794688	0.6479407765
				H	−0.5558359045	2.9682010628	−0.9583008375
				H	−1.5615625730	0.1272607362	1.5734843031

mzCloud (+83.1 kJ/mol)

N	2.6940826995	−0.1415735732	0.1553320921	H	2.7317931376	0.5293636362	−0.5990564993
C	1.6586720798	−0.9550259788	0.4000228442	H	3.5015389940	−0.2161348007	0.7634992432
N	0.5220205826	−0.7763716934	−0.6509657360	H	0.4090071805	0.2209764158	−0.9067835631
C	−0.8375500745	−1.2530320714	−0.1543019784	H	0.7936974081	−1.2940050297	−1.4941262201
C	−1.4816826779	−0.1527873412	0.6990727693	H	−1.4235349707	−1.4691640482	−1.0506491555
C	−1.6050540778	1.1411446096	−0.0669566092	H	−0.6610640298	−2.1722318653	0.4045245633
C	−0.8907104374	2.2446022614	0.1934677779	H	−2.4693271927	−0.5232949039	0.9970729340
O	1.5078714681	−1.7731378652	1.2600635634	H	−0.9059372700	−0.0108534527	1.6204258061
				H	−2.3231818363	1.1529300709	−0.8876115591
				H	−0.2008643187	2.2958887937	1.0343103128
				H	−1.0195766643	3.1528068357	−0.3875405857

mzCloud (+100.4 kJ/mol)

N	−2.6965416265	−0.6394825401	1.0871962476	H	−2.2413864068	−0.4044261586	1.9759711323
C	−2.0434610357	0.0608785799	−0.2053515004	H	−2.7172614775	−1.6605600364	0.9844768749
N	−0.7223987515	−0.0016914016	−0.1705465977	H	−3.6663802409	−0.3036475946	1.1118301830
C	0.1213588301	0.5415700364	−1.2626123134	H	−0.2186675853	−0.4922782705	0.5660308868
C	1.5122092720	0.8818297643	−0.7026609560	H	0.1856942428	−0.2099957824	−2.0567614768
C	2.1630971711	−0.3047277887	−0.0390774212	H	−0.3772829438	1.4256331390	−1.6643094073
C	2.4268596429	−0.3896746628	1.2711293585	H	1.4312856542	1.7177488250	0.0011906358
O	−2.8329128987	0.5206427420	−0.9768464857	H	2.1203828654	1.2264916887	−1.5473078673
				H	2.4225615341	−1.1408378805	−0.6896495094
				H	2.2253964711	0.4375366494	1.9495041365
				H	2.9074472828	−1.2651093086	1.6974940797

3.3.8 MS^3 fragment ion, m/z 113 (via fragment ion m/z 159)

This work (0.0 kJ/mol)

O	2.4687072733	1.0480805987	−1.3135185528	H	0.3330062893	2.3474736700	−0.8778538969
C	1.5148555917	0.7730531997	−0.6333683443	H	−1.1992453133	2.0449528816	0.8049931217
N	0.3801179615	1.4532922611	−0.4010621342	H	−0.0584494772	0.9753576491	1.5959789615
C	−0.59725479700	1.1468921636	0.6583421571	H	−1.9640188874	−0.4008835807	1.2524654710
C	−1.5030984775	−0.0465858244	0.3256991403	H	−2.3132988394	0.2501055775	−0.3454394761
C	−0.7461660557	−1.2180765128	−0.3727778255	H	−0.6541133922	−0.9822319427	−1.4432650646
C	0.6350371323	−1.4540008523	0.1209612284	H	−1.3185348459	−2.1434873180	−0.2937009620
N	1.5701925615	−0.5589707632	0.0685805478	H	0.9263632185	−2.4234341228	0.5260260174
				H	2.5256000574	−0.8116370845	0.3280396112

This work (+8.2 kJ/mol)

N	2.0726841309	0.0087370126	0.3804199882	H	2.6553190778	−0.5031094075	−0.2825639242
C	0.6989871666	−0.3560092083	0.2923636498	H	2.4490045092	−0.1369238971	1.3149880250
C	−0.0102233620	−1.3900859266	0.7554175677	H	0.4118749743	−2.2205629885	1.3084481252
C	−1.4768561362	−1.2411905658	0.4489473448	H	−1.8982117862	−2.1424628872	−0.0044617500
C	−1.5233106575	−0.0409519890	−0.5278459741	H	−2.0451806570	−1.0458394839	1.3662970062
N	−0.1703820119	0.5790664934	−0.3827146270	H	−2.2933961950	0.6934009995	−0.2896966837
C	0.1442912793	1.7903720637	−0.7692319702	H	−1.6382685126	−0.3689099188	−1.5640118357
O	1.3146914686	2.3251943794	−0.6184391313	H	−0.6114242467	2.4057968332	−1.2511959259
				H	1.9206009582	1.6433784910	−0.1768198848

This work (+76.2 kJ/mol)

N	−0.2819765695	2.0640174851	0.0430994179	H	0.1726734513	2.8358777156	0.5185588252
C	0.4969253173	0.9256112603	−0.0631190228	H	−0.7493717496	2.3838166979	−0.8021104338
C	1.7672650485	0.6066116503	0.1912963296	H	2.5320352832	1.3391067118	0.4209166878
C	2.0433795266	−0.8678641734	0.0838108169	H	2.5457981039	−1.2689865877	0.9690572245
C	0.6401343241	−1.4905220995	−0.0773273264	H	2.6861388156	−1.0856959752	−0.7771133942
N	−0.2915063838	−0.3126947773	−0.4007546636	H	0.2817576145	−1.9273133219	0.8579399850
C	−1.6182208057	−0.3670981250	0.3660382087	H	0.5469430805	−2.2293905471	−0.8729968328
O	−2.6325649333	−0.4930339082	−0.2319122808	H	−1.4601510476	−0.2972430779	1.4510042088
				H	−0.5580460760	−0.3210359277	−1.3949077501

mzCloud (+80.6 kJ/mol)

N	−1.3298565403	0.8665391319	0.9735267918	H	−0.4520668533	0.7036890940	1.4720250640
C	−1.4378621194	0.5302036869	−0.3066605526	H	−2.0556122786	1.3925945918	1.4414490859
N	−0.6399654654	−0.3453254235	−0.8973533053	H	−0.7614473118	−0.4119328148	−1.9022438062
C	0.2348647527	−1.3364612829	−0.2362427776	H	−0.2586103110	−1.6717865893	0.6805285411
C	1.6650871093	−0.8425904877	0.0573646853	H	0.2810880667	−2.1896154926	−0.9156915192
C	1.7498693015	0.1146568858	1.1631822571	H	2.2675313875	−1.7244446825	0.3089224108
C	1.8301214591	0.8849940041	2.0943681570	H	2.1056711628	−0.4116744315	−0.8494343675
O	−2.3847266364	1.0302367886	−1.0860170602	H	1.9635106030	1.5581110907	2.9141817209
				H	−2.7776963266	1.8528059311	−0.7523053252

mzCloud (+105.2 kJ/mol)

N	2.0826474245	−0.2492144008	1.3546308100	H	2.2249057880	0.7438267948	1.2355740725
C	1.3103285412	−1.0066458262	0.5682000620	H	2.5132231930	−0.6960630513	2.1563551691
N	0.7529121341	−0.2107192140	−0.6516805889	H	1.4291230162	−0.3152303070	−1.4167790929
C	−0.6136331750	−0.7008365234	−1.1003543125	H	0.6513550754	0.7980360189	−0.4451859904
C	−1.6897762464	−0.1764880341	−0.1357308831	H	−0.5715399047	−1.7898303494	−1.1201343297
C	−1.5300658338	1.2606199970	0.0962701480	H	−0.7572556041	−0.3079238024	−2.1085421682
C	−1.3081525799	2.4393171094	0.2649623962	H	−2.6682567712	−0.3893717134	−0.5811349929
O	1.0242576217	−2.1663563906	0.6464098820	H	−1.6541256152	−0.7243619744	0.8133843139
				H	−1.1959470639	3.4909416668	0.4236555049

mzCloud (+122.1 kJ/mol)

N	1.0368137235	1.0083338827	1.3049887939	H	0.0050973010	0.8610323367	1.4084713669
C	1.7280093033	−0.2485914271	0.6989027009	H	1.4644826718	1.1271053809	2.2302473056
N	1.1614361562	−0.6667276082	−0.4403941491	H	1.2106277597	1.8592537240	0.7585857113
C	0.0063556028	−0.1280058645	−1.1786038664	H	1.6118615191	−1.4970358784	−0.8128266412
C	−1.3618402153	−0.6550697319	−0.6948414701	H	0.1505061667	−0.4170189828	−2.2212850055
C	−1.7412025680	−0.1851227216	0.6407358404	H	0.0299443508	0.9660079785	−1.1588807384
C	−2.0588882542	0.2135229319	1.7412158318	H	−2.1206983476	−0.3257751209	−1.4154345445
O	2.6616326342	−0.6845479619	1.3117008548	H	−1.3627037961	−1.7513186890	−0.7120022649
				H	−2.4213340077	0.5239577517	2.6990202744

3.4 Homocitrulline

3.4.1 Precursor ion, m/z 190

Protonated homocitrulline (0.0 kJ/mol)

N	3.5472975628	−0.8144451026	0.4687403521	H	0.0887002164	1.7678414809	2.4352591217
C	2.4946873735	−0.2804177331	−0.2135831490	H	4.1488140714	−1.5143279354	0.0589337519
O	1.9660576369	0.7913263728	0.1720016641	H	2.4949018845	−1.8384521373	−1.5238873020
N	2.0236786016	−0.9783148256	−1.2784818301	H	1.1978004408	0.6587067769	−2.2645082932
C	1.0892593872	−0.4296281941	−2.2731354837	H	1.4388113698	−0.7811075874	−3.2481520207
C	−0.3922499945	−0.8157463372	−2.1097075142	H	−0.4845445577	−1.9003200422	−1.9697945440
C	−1.1581249132	−0.0585350276	−1.0080681458	H	−0.8718991475	−0.5920995557	−3.0696340236
C	−0.9818961979	−0.6449596021	0.4080664372	H	−2.2259010519	−0.0540596517	−1.2541330011
C	−1.2176838977	0.3648212454	1.5493917406	H	−0.8417520288	0.9921180468	−1.0423643225
N	−0.1992165883	1.4786127840	1.4981714013	H	0.0208424633	−1.0584371470	0.5432615722
C	−2.5896828553	1.0386420423	1.4863809508	H	−1.6787556247	−1.4745668605	0.5622101003
O	−2.7288023857	2.2172551357	1.2317965006	H	−1.1161994979	−0.1433962152	2.5119819970
O	−3.5746223876	0.1742325563	1.7245700870	H	0.6760595329	1.9581164930	0.9464359034
				H	−0.6470164139	2.2994237696	1.0641083233
				H	−4.4320925364	0.6341903408	1.6643264799
				H	3.9535295380	−0.2342682459	1.1881132466

3.4.2 MS^2 fragment ion, m/z 173

This work (0.0 kJ/mol)

O	−2.0808492620	0.2274194524	−2.1362140677	H	−1.0061879963	−1.8424436869	−2.5507944052
C	−1.1230459503	−0.2349478575	−1.5585923865	H	−0.7525800740	2.5483930399	0.1903616853
O	−0.5421496601	−1.4038088933	−1.8124735917	H	−1.7161070662	2.0135954037	−1.0891301637
C	−0.4747723567	0.5117492738	−0.3537443115	H	−2.2661311271	0.0907638365	0.7745052871
N	−0.7858270032	1.9672169799	−0.6500598596	H	−1.0020211277	0.9298449410	1.6833661033
C	−1.1880573230	0.1212492671	0.9639988929	H	−0.9242851802	−2.0403659968	0.9268506586
C	−0.6759371305	−1.1892746116	1.5712652464	H	−1.1891971441	−1.3528317514	2.5229990838
C	0.8385912385	−1.0947224044	1.7762771572	H	1.2333348504	−2.0257198857	2.1940258185
C	1.5397017118	−0.8365868925	0.4477079496	H	1.0737474419	−0.2959152296	2.4904698237
N	0.9791750750	0.3492956729	−0.2714310580	H	2.6019797877	−0.6330837325	0.5976167757
C	1.7913654824	1.0136891327	−1.1477142217	H	1.4459920109	−1.7104662091	−0.2053991037
O	1.4074792279	1.8889526236	−1.9324132480	H	2.8486706865	0.7268922237	−1.0843562136
				H	−0.0328891115	2.2710053043	−1.3471218513

HMDB MoNA trans (+5.4 kJ/mol)

O	2.3056359237	−2.1599752045	−2.3573025964	H	2.1486148087	−3.0962288866	−2.1367384275
C	1.5466623790	−1.3811182069	−1.5978593058	H	2.7713479683	0.3154223884	−2.0185394559
C	1.7025757755	0.0966213293	−1.9211989429	H	1.2620479374	0.2564651256	−2.9137089403
C	1.0224274556	0.9181466032	−0.8638839528	H	1.5613783470	1.0313313186	0.0773163698
C	−0.2273056903	1.3834710770	−0.9630223014	H	−0.7642050479	1.2871483253	−1.9085043206
C	−1.0200803565	1.8741767154	0.2180137879	H	−0.3629612592	2.1233081198	1.0581329111
C	−2.0329846971	0.7971523665	0.6451698738	H	−1.5991578100	2.7742087417	−0.0163803106
N	−1.3476261921	−0.5388224436	0.8892939389	H	−2.7708509509	0.6118467540	−0.1391782726
C	−0.6426556013	−0.6315808037	2.2664580230	H	−2.5442721085	1.0561824028	1.5731233584
N	0.3940293655	−1.4750757769	2.2200811480	H	−0.6662838085	−0.7522014989	0.1295230629
O	−1.0992268945	−0.0034529770	3.1814533604	H	−2.0523179244	−1.2841623965	0.8779525929
O	0.7897399835	−1.8304082928	−0.7352761623	H	0.8961866539	−1.6491894065	3.0817274115
				H	0.7292817428	−1.8848653737	1.3535471503

HMDB MoNA cis (+14.2 kJ/mol)

O	2.1349206574	2.1752278879	−2.0212116543	H	1.6284449041	3.0045089177	−2.1019462041
C	1.4733006850	1.3039374589	−1.2764274506	H	2.5259328218	−0.0210029854	−0.0200013706
C	2.2053460769	−0.0081239540	−1.0689835422	H	3.1091770057	−0.0088219710	−1.6822791721
C	1.3293775715	−1.1949034364	−1.4031945551	H	1.2763824985	−1.4369403284	−2.4625029751
C	0.6291432133	−1.9599464066	−0.5553922365	H	0.0557916105	−2.7775884580	−0.9908214470
C	0.5106986940	−1.8467810300	0.9452916088	H	1.2946438518	−1.2174373325	1.3837515315
C	−0.8759443203	−1.3483444223	1.3830480561	H	0.6378128565	−2.8407100097	1.3899862087
N	−1.0011568812	0.1456603483	1.1403777248	H	−1.0668288555	−1.5165582548	2.4431038609
C	−2.4552820948	0.6390014710	1.0258391331	H	−1.6752725875	−1.8288670870	0.8170228946
N	−2.5464111740	1.6903478813	0.2001690655	H	−0.5886018599	0.6441024470	1.9364294955
O	−3.3015996164	0.0789110544	1.6667574073	H	−0.4462593245	0.4545636723	0.3046457327
O	0.3689425640	1.5575627795	−0.7880353242	H	−1.7628276823	2.0433559726	−0.3364840514
				H	−3.4599306146	2.1069457852	0.0708572637

HMDB Wishart Lab cis (+272.1 kJ/mol)

N	−1.6533547214	1.0071800976	−2.2879063310	H	−2.1329680641	1.8874659895	−2.1269629944
C	−1.0446390638	0.4950337112	−1.1150815767	H	−1.0367999033	1.0724613801	−3.0888490355
O	−1.0192237597	1.3541163284	−0.0409756299	H	−0.0884188179	0.0099567109	−1.3269518418
N	−1.9881256054	−0.6691143108	−0.6214109846	H	−0.1026070985	1.6368035392	0.1906566829
C	−1.4740051655	−1.5290031836	0.5227086630	H	−2.8535448710	−0.1971799245	−0.3341292939
C	−0.4945928987	−2.5750848057	0.0687108964	H	−2.2254336225	−1.2346131921	−1.4422513517
C	0.8465387777	−2.4305050794	0.1139617416	H	−1.0403516432	−0.8295365193	1.2390957671
C	1.4982880218	−1.2290480053	0.5063397819	H	−2.3525339873	−1.9956736588	0.9751742302
C	2.0224584572	−0.1699609271	0.7826381838	H	−0.9022473521	−3.5245229789	−0.2684952166
C	2.5667984289	1.1645916849	1.0769074525	H	1.4807479586	−3.2620981067	−0.1861705261
O	2.9174708861	1.3589165147	2.4091173808	H	3.4830050519	1.3460494529	0.5059714106
O	1.5442553831	2.0858294647	0.6450447036	H	2.1978437326	1.0745664495	2.9935525516
				H	1.8515398764	2.9915693686	0.8094053358

HMDB Wishart Lab trans (+305.2 kJ/mol)

N	−3.9267764227	−0.3858664705	−0.1803830159	H	−3.9332506835	−0.9798526893	−0.9997884400
C	−2.7504280371	0.3610254257	0.0285337315	H	−4.7953306875	0.1255634380	−0.0640440811
O	−2.8464842355	1.7504458880	−0.1121659395	H	−1.9105914385	−0.0757904460	−0.5208655550
N	−2.3644346046	0.2483121409	1.5178188485	H	−3.0017410055	1.9877008843	−1.0385006765
C	−0.9813711972	0.7866429287	1.9095195401	H	−2.4517666492	−0.7423064933	1.7664605296
C	0.1132315257	−0.0100419116	1.2794983869	H	−3.0874371169	0.7498087292	2.0481564108
C	0.9767814657	0.5251932884	0.3906626374	H	−0.9571529513	0.7314792698	3.0028066006
C	2.0548546977	−0.1750721601	−0.2035191198	H	−0.9708426370	1.8332237727	1.6032275172
C	2.9807462543	−0.7380010718	−0.7504675554	H	0.2388068601	−1.0420413794	1.6024744418
C	4.1308023838	−1.4278574119	−1.3725631914	H	0.8638072325	1.5714519730	0.1018374710
O	3.6350406873	−2.0612162347	−2.5313326807	H	4.5457950275	−2.1546058470	−0.6630510405
O	5.1961284342	−0.5520761335	−1.6411246565	H	4.3441970535	−2.6091732620	−2.9008206926
				H	4.8974160441	0.1210537721	−2.2722694701

3.4.3 MS^2 fragment ion, m/z 147

This work (0.0 kJ/mol)

N	1.6267719813	−0.9900422230	−0.2077376982	H	1.7005994693	−1.8626179901	0.3140420143
C	0.5409326741	−1.1131519856	−1.2036464753	H	2.5231698877	−0.8682208876	−0.6789485617
C	−0.8026208050	−1.5079269402	−0.5254253882	H	0.7585028919	−1.8924911666	−1.9452483919
C	−1.1492960662	−0.8597422688	0.8300097890	H	−0.7711227757	−2.5922300619	−0.3690540607
C	−1.3606449289	0.6792893843	0.8709148762	H	−1.6081697655	−1.3360343035	−1.2466505686
C	−0.3348518468	1.4488185598	1.7062293585	H	−0.4002300387	−1.1488792653	1.5781577229
N	1.0431320544	1.4199813051	1.0896506731	H	−2.0729894656	−1.3439817037	1.1615866871
C	0.4335934343	0.1819388792	−2.0074230096	H	−2.3307787871	0.8984771155	1.3283875804
O	−0.2620100905	−0.0078884937	−3.1311559623	H	−1.4151704475	1.1089745446	−0.1357292894
O	0.9123687375	1.2610770028	−1.6946649168	H	−0.6173275965	2.4988324518	1.8134413440
				H	−0.2417266930	1.0147775948	2.7052229276
				H	1.3366513559	0.4324253597	0.7906278653
				H	1.7446856568	1.8246773925	1.7128969697
				H	1.0614521416	1.9227272532	0.1916504471
				H	−0.3150209778	0.8311104465	−3.6238339321

3.4.4 MS^2 fragment ion, m/z 144

This work, HMDB MoNA (0.0 kJ/mol)

N	−2.2931608409	−0.2919383841	−0.3330825568	H	−1.5096982913	−0.8371575557	0.1514358554
C	−2.3101873867	0.9924417017	−0.4015120996	H	−3.0181770353	−0.8320689592	−0.7993104462
C	−1.2776315370	1.8545585853	0.2296764642	H	−3.1165618118	1.4505337843	−0.9754855966
C	−0.0416170085	2.0848870181	−0.7043106959	H	−0.9562316901	1.4027634417	1.1723502279
C	0.8039363611	0.8301234055	−0.9717279257	H	−1.7325198551	2.8233974326	0.4541492656
C	1.5192618063	0.2820748484	0.2838773621	H	0.5639740500	2.8579056607	−0.2182791007
N	1.7761386027	−1.1552123917	0.2342996847	H	−0.3779530030	2.5141047791	−1.6541777139
C	0.8034008813	−2.0622689800	0.5167949545	H	0.1830590545	0.0378871439	−1.4037833638
N	1.1475016872	−3.3757491312	0.5318388701	H	1.5461958698	1.0720013178	−1.7401938326
O	−0.3805417991	−1.7123800351	0.7457055121	H	0.9332720797	0.4779243066	1.1863887703
				H	2.4820439306	0.7802954621	0.4215523546
				H	2.7108976251	−1.4758510204	0.0270443993
				H	2.1067855494	−3.6877978973	0.5469407715
				H	0.4380127609	−4.0304745333	0.8255088395

3.4.5 MS^2 and MS^3 fragment ion, m/z 130 (via fragment ion m/z 147)

This work (0.0 kJ/mol)

C	0.3765727213	0.1012445173	−0.4035340196	H	0.3111898085	0.2311159883	−1.4892877759
C	−0.3053041048	1.2733288372	0.3203926457	H	−0.1457407801	1.1708332958	1.4022959344
C	−1.8079743054	1.3312864524	0.0035629187	H	0.2009063246	2.1916878654	0.0117117541
C	−2.4967159219	−0.0023451453	0.3280110436	H	−1.9542167912	1.5796288996	−1.0554701864
C	−1.8291012870	−1.1685113119	−0.3947572555	H	−2.2675601968	2.1382873859	0.5807142327
N	−0.3425195228	−1.1941936262	−0.0782744967	H	−3.5490459114	0.0178795607	0.0286869173
C	1.8340170596	−0.1114842075	0.0013679913	H	−2.4857374312	−0.1851387148	1.4107719027
O	2.2014621708	−1.1079349925	0.5875847873	H	−1.9072158891	−1.0758530761	−1.4816900770
O	2.5958429563	0.9162562310	−0.3605956786	H	−2.2293332350	−2.1398069483	−0.0954965156
				H	0.1193530749	−1.9672804140	−0.5695447126
				H	3.5171338881	0.7594435829	−0.0809436677
				H	−0.1872556275	−1.3993821799	0.9169052579

HMDB Wishart Lab trans (+115.5 kJ/mol)

C	3.1004270690	1.2319098788	0.2812680101	H	4.1730725737	1.2007583167	0.0756791308
C	2.3913630212	0.1152879455	−0.5208187158	H	2.7312918746	2.2228477094	−0.0000659579
C	0.9174894895	0.1466354005	−0.3401000632	H	2.9560328654	1.0994713893	1.3571857164
C	0.1722827317	−0.9056558603	0.0979108456	H	2.5904114217	0.2825294435	−1.5907441081
C	−1.2437360420	−0.8698805077	0.2902891168	H	2.7959247957	−0.8686344321	−0.2636304698
C	−2.0987872752	0.3970218241	0.0655153826	H	0.4210827372	1.0833192110	−0.5871312830
O	−3.4279873846	0.0153308284	0.2877020823	H	0.6683880326	−1.8469770423	0.3291975071
O	−1.8866947657	0.9576894356	−1.1963654786	H	−1.7683385856	1.1561067979	0.7852829814
N	−1.8888198711	−1.9310745861	0.7066840690	H	−3.9974581432	0.7956146067	0.3603511570
				H	−2.2941508573	0.4015918527	−1.8804189578
				H	−2.8954799297	−1.8743245762	0.8477095414
				H	−1.4162137577	−2.8092676351	0.8943994935

HMDB Wishart Lab cis (+135.7 kJ/mol)

O	−3.1818949218	−0.2663262316	−0.8805924780	H	−4.0183136034	0.1771629760	−1.0879685456
C	−2.6041085693	0.2939422835	0.2537063961	H	−2.6873607856	1.3913823260	0.2728932776
O	−3.1698410339	−0.1660577472	1.4564935227	H	−3.6057385778	−1.0181607546	1.2892756078
C	−1.0950368391	−0.0271757133	0.2072681076	H	−1.0176075930	0.3788963975	−1.8708249854
C	−0.3797512376	0.1728610995	−1.0187940757	H	1.2880881241	0.3031030366	−2.2438164742
C	0.9677415102	0.1282142829	−1.2165614142	H	1.8967472249	0.4701851176	0.6797419798
C	2.0727154461	−0.1177574517	−0.2346261816	H	2.0370108731	−1.1801261014	0.0636895385
C	3.4682535626	0.2019943486	−0.7889866125	H	3.5485558638	1.2571354276	−1.0660400504
N	−0.5916673058	−0.4189528852	1.3481942513	H	4.2340357284	−0.0077521196	−0.0386540949
				H	3.6880009077	−0.4050429971	−1.6722431691
				H	0.3854912282	−0.6489287591	1.4754579952
				H	−1.2353200017	−0.5185965349	2.1344874050

3.4.6 MS^2 and MS^3 fragment ion, m/z 127 (via fragment ion m/z 144)

This work (0.0 kJ/mol)

N	2.9074898523	0.8227430460	0.1081289507	H	2.5996188730	1.1489655380	1.0108331188
C	2.1044350037	0.3196213605	−0.8414254529	H	3.8666672455	1.0058813125	−0.1596435449
N	0.6767657383	0.0967776914	−0.3814224023	H	0.2395315511	−0.1805742601	−2.3265492901
C	−0.1827482562	−0.1443173683	−1.3219399338	H	−2.1681384795	0.2258990082	−1.8476298121
C	−1.6269568481	−0.3756539972	−1.1040263183	H	−1.8077655872	−1.4163194694	−1.4242608623
C	−2.0988849673	−0.1213378065	0.3322738465	H	−3.0421443558	−0.6430702137	0.5094590376
C	−1.0255080006	−0.5919317029	1.3179229240	H	−2.2970072855	0.9475675786	0.4725434246
C	0.3019141941	0.1248776870	1.0673193863	H	−1.3233817105	−0.3938421495	2.3513434967
O	2.3907274176	0.0357336816	−1.9762438863	H	−0.8756465688	−1.6747359339	1.2308392858
				H	0.2486235080	1.1771543946	1.3695026513
				H	1.1123086760	−0.3633383966	1.6129753808

HMDB MoNA (+131.7 kJ/mol)

C	3.0815030583	−0.8340464585	−0.9822824529	H	2.7014218589	−1.5262157410	−1.7401169863
C	1.9980639127	−0.1535094291	−0.2732338930	H	3.6998637343	−1.4028766954	−0.2808047541
C	1.1572520353	0.4579663690	0.3565519473	H	3.7223996831	−0.1014753924	−1.4833568672
C	0.1198350731	1.1724391176	1.1091651969	H	−0.2966190742	1.9895315462	0.5084466882
C	−1.0006443740	0.2294286151	1.5784044499	H	0.5484429094	1.6400273018	2.0033924552
N	−1.4086992685	−0.7112234075	0.4510160248	H	−0.6610069211	−0.4176547039	2.3899190624
C	−2.1924644886	−0.0384270797	−0.7042484810	H	−1.8974760460	0.7676247761	1.8901202688
N	−1.3894629769	0.3602667648	−1.6964695473	H	−2.0526183257	−1.4224672970	0.8138525610
O	−3.3822957699	0.0329431969	−0.5744968112	H	−0.5513445295	−1.1772502256	0.1159218755
				H	−1.8193283476	0.8165400954	−2.4924709297
				H	−0.3771221431	0.3184786470	−1.6402098070

HMDB Wishart Lab cis (+224.6 kJ/mol)

C	2.1509085307	0.0056714150	2.3191569981	H	2.5102261262	0.7203282292	3.0287574248
C	1.7766108399	−0.8463867207	1.5386025636	H	1.3539192672	−2.8778936373	1.0020357975
C	1.3298335148	−1.8537983410	0.6354061116	H	0.5737766350	−2.4500563183	−1.2304543105
C	0.8849446525	−1.6129138518	−0.6138513609	H	1.8434853456	0.1670377531	−1.3784301714
C	0.8377568557	−0.2491839704	−1.2545258406	H	0.3446742248	−0.2699971508	−2.2254837433
N	0.0798071506	0.7603924929	−0.4082029224	H	0.4978211406	0.7947552385	0.5340065202
C	−1.4320352506	0.5235113475	−0.2330541391	H	0.1844236284	1.6957077674	−0.8181565193
N	−1.9199593655	1.4660819749	0.6941250765	H	−1.5231127203	−0.4934926421	0.1618256706
O	−1.8803456007	0.6522686352	−1.5526506424	H	−2.2735304921	2.3281321516	0.2930958672
				H	−2.5165342373	1.1025808882	1.4258774206
				H	−2.8229702452	0.4373547387	−1.6135798009

HMDB Wishart Lab trans (+238.6 kJ/mol)

C	4.4414558523	0.1901362205	0.1940165785	H	5.4392091969	0.2083795686	0.5755166165
C	3.3090873934	0.1822723491	−0.2382592648	H	1.8122672303	−0.6552132977	−1.5109313182
C	1.9981656972	0.1286619599	−0.7751189164	H	1.1924222480	1.7896401769	0.2537652617
C	1.0042444414	0.9815864661	−0.4511057663	H	−0.4117722387	0.1415439653	−1.8545975012
C	−0.3430918064	0.9193368720	−1.0932932671	H	−0.6336651089	1.8790784927	−1.5330734198
N	−1.4413584059	0.6042311670	−0.0699884834	H	−1.4321428717	1.2865787156	0.6949963100
C	−1.3910103736	−0.7912820296	0.5868258636	H	−2.3602167266	0.6825061752	−0.5228782644
N	−2.3155733983	−0.8124275491	1.6494777962	H	−0.3676311283	−0.8972453573	0.9604853432
O	−1.6698137787	−1.6081245312	−0.5148794901	H	−3.2528691768	−1.1166018479	1.4076892293
				H	−1.9861170188	−1.2208971487	2.5148273711
				H	−1.5916900267	−2.5430603674	−0.2732746784

3.4.7 MS^3 fragment ion, m/z 127 (via fragment ion m/z 173)

This work (+48.7 kJ/mol)

N	2.2597536651	−0.5634931033	0.3031257257	H	2.4926684633	−1.3410197144	0.9250509133
C	0.8013940846	−0.4618379076	0.0788377459	H	2.7845322681	−0.6744153955	−0.5712346889
C	0.0091575904	−1.5311363910	−0.0382668792	H	2.5226891864	0.4071881214	0.7560164395
C	−1.4759389922	−1.4116592672	−0.2405935081	H	0.4541761775	−2.5223732143	0.0014251817
C	−1.9705575448	0.0269138868	−0.0200687839	H	−1.9880293168	−2.1012125056	0.4398180083
C	−0.9992048890	1.0396814432	−0.6313981446	H	−1.7164746213	−1.7614349854	−1.2540495442
N	0.3491012468	0.8732986542	−0.0314969076	H	−2.0727966314	0.2297827307	1.0516578411
C	1.0395094663	1.9300259085	0.4780733125	H	−2.9579871710	0.1634301258	−0.4694412354
O	2.1544038499	1.8355708797	1.0184961854	H	−0.9087723341	0.8971085919	−1.7139330325
				H	−1.3186295662	2.0667857770	−0.4454944637
				H	0.5409050683	2.8986963651	0.3635758343

This work (+121.8 kJ/mol)

N	−2.3122702162	−0.8548462016	−0.0457634515	H	−2.7632473208	−0.9920044417	−0.9481932798
C	−0.9214943656	−0.7162332524	−0.0605152540	H	−2.6530985274	−1.5390889958	0.6208302295
C	0.0236676138	−1.4730563578	0.5065522375	H	−0.2904225754	−2.4357329171	0.9022132708
C	1.4741645127	−1.0968688122	0.6431023919	H	1.8165260591	−1.3332453892	1.6563661199
C	1.7190575545	0.3855279070	0.3329179982	H	2.0680659349	−1.7341457662	−0.0265463313
C	0.9302544229	0.8172326390	−0.8942761825	H	2.7788595783	0.5727495004	0.1378044356
N	−0.5696582624	0.6047840698	−0.6495916819	H	1.4483540653	1.0092098615	1.1936594920
C	−1.1909464067	1.7534003095	0.1958056803	H	1.0510626972	1.8724726921	−1.1469887649
O	−1.4419971565	2.7813443727	−0.3328795979	H	1.1771490855	0.2109917611	−1.7698705449
				H	−1.0337550552	0.7023853757	−1.5621107825
				H	−1.3104716380	1.4650236455	1.2475840154

HMDB MoNA (+131.7 kJ/mol)

C	3.0815030583	−0.8340464585	−0.9822824529	H	2.7014218589	−1.5262157410	−1.7401169863
C	1.9980639127	−0.1535094291	−0.2732338930	H	3.6998637343	−1.4028766954	−0.2808047541
C	1.1572520353	0.4579663690	0.3565519473	H	3.7223996831	−0.1014753924	−1.4833568672
C	0.1198350731	1.1724391176	1.1091651969	H	−0.2966190742	1.9895315462	0.5084466882
C	−1.0006443740	0.2294286151	1.5784044499	H	0.5484429094	1.6400273018	2.0033924552
N	−1.4086992685	−0.7112234075	0.4510160248	H	−0.6610069211	−0.4176547039	2.3899190624
C	−2.1924644886	−0.0384270797	−0.7042484810	H	−1.8974760460	0.7676247761	1.8901202688
N	−1.3894629769	0.3602667648	−1.6964695473	H	−2.0526183257	−1.4224672970	0.8138525610
O	−3.3822957699	0.0329431969	−0.5744968112	H	−0.5513445295	−1.1772502256	0.1159218755
				H	−1.8193283476	0.8165400954	−2.4924709297
				H	−0.3771221431	0.3184786470	−1.6402098070

HMDB Wishart Lab cis (+224.6 kJ/mol)

C	2.1509085307	0.0056714150	2.3191569981	H	2.5102261262	0.7203282292	3.0287574248
C	1.7766108399	−0.8463867207	1.5386025636	H	1.3539192672	−2.8778936373	1.0020357975
C	1.3298335148	−1.8537983410	0.6354061116	H	0.5737766350	−2.4500563183	−1.2304543105
C	0.8849446525	−1.6129138518	−0.6138513609	H	1.8434853456	0.1670377531	−1.3784301714
C	0.8377568557	−0.2491839704	−1.2545258406	H	0.3446742248	−0.2699971508	−2.2254837433
N	0.0798071506	0.7603924929	−0.4082029224	H	0.4978211406	0.7947552385	0.5340065202
C	−1.4320352506	0.5235113475	−0.2330541391	H	0.1844236284	1.6957077674	−0.8181565193
N	−1.9199593655	1.4660819749	0.6941250765	H	−1.5231127203	−0.4934926421	0.1618256706
O	−1.8803456007	0.6522686352	−1.5526506424	H	−2.2735304921	2.3281321516	0.2930958672
				H	−2.5165342373	1.1025808882	1.4258774206
				H	−2.8229702452	0.4373547387	−1.6135798009

HMDB Wishart Lab trans (+238.6 kJ/mol)

C	4.4414558523	0.1901362205	0.1940165785	H	5.4392091969	0.2083795686	0.5755166165
C	3.3090873934	0.1822723491	−0.2382592648	H	1.8122672303	−0.6552132977	−1.5109313182
C	1.9981656972	0.1286619599	−0.7751189164	H	1.1924222480	1.7896401769	0.2537652617
C	1.0042444414	0.9815864661	−0.4511057663	H	−0.4117722387	0.1415439653	−1.8545975012
C	−0.3430918064	0.9193368720	−1.0932932671	H	−0.6336651089	1.8790784927	−1.5330734198
N	−1.4413584059	0.6042311670	−0.0699884834	H	−1.4321428717	1.2865787156	0.6949963100
C	−1.3910103736	−0.7912820296	0.5868258636	H	−2.3602167266	0.6825061752	−0.5228782644
N	−2.3155733983	−0.8124275491	1.6494777962	H	−0.3676311283	−0.8972453573	0.9604853432
O	−1.6698137787	−1.6081245312	−0.5148794901	H	−3.2528691768	−1.1166018479	1.4076892293
				H	−1.9861170188	−1.2208971487	2.5148273711
				H	−1.5916900267	−2.5430603674	−0.2732746784

3.4.8 MS^n fragment ion, m/z 84

This work (0.0 kJ/mol)

N	0.7489444870	1.1561708438	0.6151977761	H	1.3123329378	1.9046726485	1.0159122197
C	−0.4633795494	1.4287418133	0.2660510551	H	−0.7929374703	2.4573238349	0.4087701697
C	−1.3883213555	0.4245891533	−0.3110061877	H	−2.3597581351	0.5446667359	0.1873871268
C	−0.8580112170	−1.0157400968	−0.2381844410	H	−1.5738977623	0.7473546502	−1.3493041711
C	0.6507311547	−1.0482423456	−0.5192578465	H	−1.0630581631	−1.4302297852	0.7555362355
C	1.4092490115	−0.1715249584	0.4748348953	H	−1.3961966708	−1.6386006452	−0.9562752800
				H	0.8550002284	−0.7040132680	−1.5402083712
				H	1.0394892770	−2.0672128382	−0.4436846389
				H	2.4363236365	0.0252673013	0.1572067817
				H	1.4432895904	−0.6133230435	1.4770246766

HMDB MoNA (different molecular formula)

O	−2.6239295668	−0.5868734365	0.7694894506	H	−0.7158842675	−0.6219806662	1.5505126962
C	−1.4753819959	−0.3154913825	0.8204874828	H	−1.6540725432	0.7959717787	−0.9357842672
N	−0.8820532562	0.5869392041	−0.2881364476	H	−0.5699296073	1.4750792188	0.1290930950
C	0.3062622721	−0.0466037838	−1.0404040409	H	0.5188101040	0.6176570552	−1.8839246552
C	1.4475448539	−0.1874575913	−0.1568649067	H	−0.0505173738	−1.0051667808	−1.4301103527
C	2.4171846779	−0.3032569206	0.5541865592	H	3.2820667028	−0.4088166948	1.1762553866

3.4.9 MS^4 fragment ion, m/z 84 (via MS^3 fragment ion m/z 190 $\rightarrow$ 173 $\rightarrow$ 127)

This work (+50.3 kJ/mol)

N	1.2369768116	0.6932642563	−0.3597505357	H	1.4667733084	0.5600009125	−1.3526509860
C	1.1042354911	−0.6693455481	0.3126396342	H	2.0372452834	1.1898202378	0.0463608736
C	−0.2061399580	−1.3164234353	−0.1176136617	H	1.1329696831	−0.4762967417	1.3879081944
C	−1.4095402795	−0.4516435808	0.2874616527	H	1.9836748144	−1.2500532076	0.0269586891
C	−1.1580489489	1.0113856351	0.0607036673	H	−0.1982292074	−1.4762395401	−1.2031412094
C	0.0218462470	1.5488152734	−0.2391358658	H	−0.2656486347	−2.3075587255	0.3406892186
				H	−1.6631181460	−0.5988779768	1.3463627513
				H	−2.3015426527	−0.7532515173	−0.2723503675
				H	−1.9962933172	1.6974498676	0.1537916151
				H	0.2150395055	2.5990540906	−0.4182336702

HMDB MoNA (different molecular formula)

O	−2.6239295668	−0.5868734365	0.7694894506	H	−0.7158842675	−0.6219806662	1.5505126962
C	−1.4753819959	−0.3154913825	0.8204874828	H	−1.6540725432	0.7959717787	−0.9357842672
N	−0.8820532562	0.5869392041	−0.2881364476	H	−0.5699296073	1.4750792188	0.1290930950
C	0.3062622721	−0.0466037838	−1.0404040409	H	0.5188101040	0.6176570552	−1.8839246552
C	1.4475448539	−0.1874575913	−0.1568649067	H	−0.0505173738	−1.0051667808	−1.4301103527
C	2.4171846779	−0.3032569206	0.5541865592	H	3.2820667028	−0.4088166948	1.1762553866

3.5 Arginine

3.5.1 Precursor ion, m/z 175

Protonated arginine (0.0 kJ/mol)

N	0.7622744385	−1.1308421204	1.6298784486	H	0.8207014319	−0.7711142026	2.5808534762
C	1.8370241698	−0.5758263593	0.7912511395	H	0.8601557171	−2.1422383431	1.6907975997
C	1.7545010920	−1.1014973058	−0.6690949536	H	2.8340999644	−0.8492126428	1.1649237067
C	0.6549749532	−0.5577718934	−1.6073848965	H	1.6863630386	−2.1958686764	−0.6131723529
C	−0.8080605107	−0.9401665069	−1.3137489795	H	2.7182862950	−0.8830815382	−1.1384126181
N	−1.2967674205	−0.3203107877	−0.0765139775	H	0.7261892584	0.5347502872	−1.6823642861
C	−2.1757628541	0.6687328581	0.0359677310	H	0.8811078732	−0.9415831319	−2.6093240833
N	−3.1448590996	0.8650143890	−0.8815821629	H	−0.9148557004	−2.0269603327	−1.2248564309
N	−2.0947329652	1.4865687495	1.0988936989	H	−1.4301613638	−0.6184604620	−2.1519631879
C	1.7680194485	0.9457054811	0.8609707260	H	−0.7043485348	−0.5455279151	0.7518243332
O	2.8272024274	1.5156671165	0.2755746987	H	−3.4379588840	0.1191277330	−1.4938510913
O	0.8710527506	1.5831290091	1.3872076536	H	−3.6950129707	1.7106718569	−0.8821472967
				H	−2.8606685021	2.0872445933	1.3635780227
				H	2.7556367852	2.4833878860	0.3594897550
				H	−1.1942008376	1.6004622584	1.5565053274

3.5.2 MS^2 fragment ion, m/z 158

This work, Zhang et al. (0.0 kJ/mol)

N	−1.1094087141	2.6149848628	0.5818439965	H	−1.9401172801	3.0307290101	0.1844529033
C	−0.8342023871	1.3110199846	0.4011894426	H	−1.9553395809	1.1500660952	−1.2470242983
N	−1.4969720067	0.6135789760	−0.5221309603	H	−2.1825811878	−1.1282041924	0.4234449904
C	−1.7114878393	−0.8487097934	−0.5260041509	H	−2.4362172211	−1.0346407829	−1.3204584881
C	−0.4428278738	−1.6717298076	−0.7585082148	H	0.0513008809	−1.3378556775	−1.6790302867
C	0.5218658408	−1.6472019713	0.4295592490	H	−0.7527506042	−2.7081473499	−0.9299736871
C	1.0780050632	−0.2549189377	0.7909618958	H	0.0247296211	−2.0439173224	1.3218733598
C	1.9287481928	0.3687399490	−0.3302832244	H	1.3788551942	−2.2940991702	0.2244416909
O	1.6975836009	1.4422610649	−0.8428072291	H	1.7395371778	−0.3858911663	1.6528390490
O	2.9563539214	−0.4265361131	−0.6512416447	H	3.4886557288	0.0025407066	−1.3455511398
N	0.0602140255	0.7177159823	1.2236802814	H	0.3226034711	1.2832071885	2.0217129639
				H	−0.3862480234	3.2471084648	0.8970135019

This work, Zhang et al. (+1.7 kJ/mol)

N	−1.3292996900	2.6075117036	0.6241426572	H	−0.6431193551	3.3107388809	0.8597796584
C	−0.9593189302	1.3455342631	0.3336936680	H	−2.7323847764	0.5874498151	0.8728445078
N	−1.8692804493	0.3616668859	0.3953238653	H	−1.8652179272	−0.5184405686	−1.5153243330
C	−1.8864330003	−0.8437878765	−0.4682942488	H	−2.8552916033	−1.3129709553	−0.2885873374
C	−0.7647045870	−1.8474825895	−0.1996827191	H	−0.7887972037	−2.1538652348	0.8533536749
C	0.6318685054	−1.3453719780	−0.5763117740	H	−0.9869354309	−2.7410450218	−0.7928956366
C	1.0685867138	−0.1290761682	0.2596380852	H	0.6759922616	−1.0851354973	−1.6409264558
C	2.5333333866	0.2582301498	0.0241305258	H	1.3603765182	−2.1419599697	−0.4044028199
O	2.8780929229	1.3687060234	−0.3234497951	H	0.9715884817	−0.3818751815	1.3249362008
O	3.3581318608	−0.7616445351	0.2634311703	H	4.2769334491	−0.4719039277	0.1165094194
N	0.3089329290	1.1020726105	−0.0087039522	H	0.9089663918	1.8892136359	−0.2577180748
				H	−2.2920204676	2.9036355358	0.5488137134

This work (+47.4 kJ/mol)

N	0.1401861097	−1.7207743561	0.4372394287	H	0.7639569525	−1.0446473198	−0.0074087406
C	−1.1917510617	−1.5972420733	0.4420952343	H	−2.7872293002	−0.4084463671	0.2456166658
N	−1.8036568305	−0.4024138993	0.4848425393	H	−2.0699342069	1.3231225836	1.6001840212
C	−1.2687791092	0.8731863015	1.0072575178	H	−0.4452540786	0.6360456007	1.6852345900
C	−0.7983769145	1.8658298301	−0.0835424527	H	−1.6315171185	2.0947267122	−0.7582384733
C	0.3536786456	1.2558982512	−0.8152487893	H	−0.5108527253	2.7952035516	0.4189854116
C	1.6502969733	1.5442308738	−0.6418493764	H	0.1129442935	0.4152567641	−1.4659839358
C	2.6596801205	0.5904061151	−1.1637639765	H	2.0003032141	2.4094653057	−0.0852139365
O	3.8407210310	1.1502992188	−1.4374729253	H	4.4572961337	0.4592187916	−1.7389043807
O	2.4326991696	−0.6094898596	−1.2799991744	H	−1.5787259356	−3.5918542058	0.1090911308
N	−1.9537957243	−2.7076095673	0.4211925692	H	−2.9155943968	−2.6936978355	0.7285177825
				H	0.5437047585	−2.6368144161	0.5801692701

HMDB MoNA (+216.4 kJ/mol)

N	−2.3565854205	−0.6054599649	−0.5580566293	H	−2.0185126571	−1.5541972012	−0.4536568966
C	−1.6033119704	0.3610038068	0.1560048689	H	−3.3591953876	−0.5677242563	−0.4162386208
N	−2.2585463929	1.1755310390	1.1154580502	H	−0.6733995856	−0.0523843962	0.5507666021
N	−1.0681523153	1.4114379841	−0.9551846829	H	−3.2716474942	1.1530204836	1.0982696092
C	−0.1844174336	0.8302985959	−2.0365777779	H	−1.9116423684	1.0605306008	2.0600183336
C	0.9909951242	0.2336075164	−1.4206275499	H	−1.8966964340	1.8425909758	−1.3773080451
C	1.8759934804	−0.2660827242	−0.7664450685	H	−0.5759140739	2.1504810882	−0.4440266377
C	2.8567532466	−0.8918800451	0.1110664174	H	−0.7927245133	0.0900949022	−2.5639937099
C	2.2409838287	−1.2075829661	1.4747428608	H	0.0680529746	1.6375485690	−2.7311899489
O	3.1440365563	−1.7811118635	2.2772569330	H	3.7319151497	−0.2489465906	0.2658251383
O	1.0922853397	−0.9731510105	1.7965377266	H	3.2392334969	−1.8253020484	−0.3204792970
				H	2.7303968496	−1.9723224946	3.1378383243

3.5.3 MS^2 fragment ion, m/z 157

This work (0.0 kJ/mol)

N	2.4838914603	0.1319058574	−1.6593096805	H	1.8415043430	−0.1825793202	−2.3878095829
C	2.0003407769	0.2782121754	−0.4306426665	H	3.7953506246	0.8368094457	0.3813572999
N	2.8118339677	0.6874363942	0.5576243446	H	2.4492712751	1.0291883643	1.4340149748
N	0.6906065998	−0.0017831015	−0.1484060994	H	1.0664546165	−0.3942339375	1.8846750623
C	0.2147171243	−0.1059972733	1.2668028493	H	−0.1725805668	0.8677943030	1.5778072221
C	−0.8504983642	−1.2000671095	1.3655181481	H	−0.3758678576	−2.1587123182	1.1287458187
C	−2.0542559145	−0.9586405195	0.4290458940	H	−1.1731218221	−1.2613816896	2.4087985870
C	−1.7323997300	0.0268161668	−0.7170596793	H	−2.3858471896	−1.9084663155	0.0019555720
N	−1.8699370821	1.4159221637	−0.2541665449	H	−2.9080964656	−0.5540240157	0.9825089742
C	−0.2872905581	−0.1279631538	−1.1822337908	H	−2.3444938582	−0.2070817045	−1.5959153645
O	0.0230812706	−0.2992999991	−2.3479567025	H	−2.7488870531	1.5403657091	0.2418748321
				H	−1.8752656312	2.0659031010	−1.0368912015
				H	3.4014900341	0.4795767772	−1.9004382665

This work (+2.0 kJ/mol)

N	3.0109478499	−0.2672736243	0.3364052429	H	3.9858020943	−0.0639126093	0.1664377894
C	2.0581557994	0.3308224636	−0.3946742795	H	1.6686233940	1.6016966498	−1.9077010002
N	2.4139334645	1.1758773650	−1.3526354492	H	3.3875098475	1.3230503042	−1.5800010505
N	0.7331971277	0.0576320644	−0.1204347157	H	0.7703358762	−1.8330272287	0.8230070952
C	0.4459258278	−0.8141451619	1.0700395875	H	1.0416258642	−0.4333352855	1.9041470261
C	−1.0242190908	−0.8240316478	1.4714509472	H	−1.1492693345	−1.6177230781	2.2141840527
C	−1.9034705401	−1.0546625078	0.2475922362	H	−1.2904981504	0.1238415459	1.9473335448
C	−1.7580802609	0.1317404411	−0.7059546349	H	−2.9555019130	−1.1624152644	0.5302804099
N	−2.3825618172	1.3315999648	−0.1324528922	H	−1.6214909689	−1.9824939400	−0.2656638127
C	−0.3062980057	0.5403445469	−0.9714586879	H	−2.1477396957	−0.1328241923	−1.7008454773
O	−0.0634297220	1.2995828182	−1.8962134881	H	−3.3567929427	1.1475103378	0.0948469728
				H	−2.3640155924	2.1031242610	−0.7948595363
				H	2.8074108889	−0.9808782225	1.0167701198

Zhang et al. (+26.3 kJ/mol)

N	1.1619042328	1.2218355880	2.3945722288	H	2.0462761190	0.9316534845	2.7888438609
C	0.8245424326	0.9369258354	1.1280442719	H	2.1156130561	−0.5714295006	0.9433895594
N	1.5435926333	0.0796440559	0.4178912239	H	1.2489202113	0.8387021630	−1.5086818441
C	1.5207458611	−0.1138303069	−1.0453018036	H	2.5550942441	−0.3249761104	−1.3279299831
C	0.6043849459	−1.2590440078	−1.5003374132	H	0.9593581082	−1.5910057999	−2.4820373805
C	−0.8853970671	−0.9097281919	−1.6405732342	H	0.7478773345	−2.1131105771	−0.8277946704
C	−1.5867337869	−0.2656911268	−0.4207116481	H	−1.4321877479	−1.8285964193	−1.8802858372
N	−1.3086966126	−0.9711263175	0.8333256561	H	−1.0318444655	−0.2399188278	−2.4952486723
C	−1.2491872492	1.2258913859	−0.3064261994	H	−2.6557113592	−0.2375744518	−0.6685263278
O	−1.8797356034	2.0875981894	−0.8706638610	H	−2.0660464674	−0.8354383853	1.4989885229
N	−0.2017047488	1.6464008360	0.5744971689	H	−1.2435683792	−1.9737358287	0.6762671253
				H	−0.2729307187	2.6285449896	0.8255445805
				H	0.4854350271	1.6380093242	3.0191546762

3.5.4 MS^3 fragment ion, m/z 140 (via fragment ion m/z 158 and fragment ion m/z 157)

No matching structures found.

3.5.5 MS^2 fragment ion, m/z 130

This work, Zhang et al. (0.0 kJ/mol)

N	−2.7663179670	−0.9474990369	−0.6934742622	H	−3.5324665336	−1.5928445238	−0.5728079254
C	−1.8158843517	−0.8045303554	0.2501806930	H	−0.4570159440	0.0030384433	−1.0394704921
N	−0.6749036358	−0.1999560280	−0.0515010596	H	0.9491591606	−0.7306824535	1.1963809754
C	0.3867325875	0.1646819878	0.8970206645	H	−0.0749177420	0.5949908214	1.7927419512
C	1.3379065491	1.2108104831	0.2953557926	H	1.9527228169	1.5776883634	1.1232654993
C	2.3001830679	0.7464550483	−0.8147297201	H	0.7517704359	2.0683971048	−0.0545015201
C	1.7267810530	0.4891177003	−2.1848465558	H	2.8408301632	−0.1696563643	−0.5253942681
O	0.5383324837	0.3483917603	−2.4380163873	H	3.0924993701	1.4959965367	−0.9455259245
N	−2.0593347105	−1.2642818520	1.4902279503	H	2.4628954308	0.4186117485	−3.0059435959
				H	−1.3355193607	−1.2928765137	2.1910386577
				H	−2.9803722270	−1.5733576261	1.7628441220
				H	−2.6429806465	−0.5425952442	−1.6104445950

3.5.6 MS^2 and MS^3 fragment ion, m/z 116 (via fragment ion m/z 158)

This work, Zhang et al. (0.0 kJ/mol)

O	1.4382851459	0.6415679065	1.8784875366	H	3.0597316921	1.8209662805	0.5928773405
C	1.6337159768	0.5732941187	0.6806043902	H	1.4696233494	−1.0566910322	−0.7188287678
O	2.5443464721	1.2479566893	−0.0054613405	H	−0.2645445600	−0.2146743348	−2.1069402705
C	0.8049873866	−0.3454368091	−0.2233420738	H	0.3031621469	1.3498115467	−1.5211611021
C	−0.1278784771	0.3966177154	−1.2102435520	H	−2.3040839648	0.7067579356	−1.1033022457
C	−1.4519004318	0.5431794035	−0.4403031877	H	−1.4123686845	1.3801744937	0.2648356055
C	−1.5770210842	−0.7838808472	0.3054029820	H	−2.1986376350	−0.7637069135	1.2013204790
N	−0.1371020258	−1.0917371376	0.7165132022	H	−1.9051162979	−1.5941802470	−0.3489682904
				H	0.0505639797	−2.0966433209	0.7377275947
				H	0.0742370114	−0.7133754476	1.6608816999

This work, Zhang et al. (+1.4 kJ/mol)

O	1.7176317382	0.5866660313	1.8701690109	H	3.3360034110	1.5932414544	0.4367223691
C	1.7849018184	0.5238269673	0.6587141173	H	1.2517006033	−1.0120794899	−0.7646507445
O	2.6990609782	1.0968589354	−0.1110116073	H	0.1960964195	0.7124054597	−2.0506640023
C	0.7547598918	−0.2447480138	−0.1660121174	H	−0.2444815477	1.6439582853	−0.6154843438
C	−0.1815796107	0.6326503807	−1.0302029541	H	−2.3765782506	0.5806968739	−1.1876769897
C	−1.5422657601	−0.0773570922	−0.9346689564	H	−1.5838534273	−0.9420239518	−1.6061316206
C	−1.6079432298	−0.5220378245	0.5256672372	H	−1.8507805373	0.3038659664	1.1973472099
N	−0.1675444160	−0.9344330694	0.8356969562	H	−2.2691173176	−1.3638560455	0.7334442848
				H	−0.0583100407	−1.9501231000	0.7928259395
				H	0.1423992773	−0.6273117674	1.7760162112

METLIN (+13.5 kJ/mol)

N	2.3518469075	1.0239573450	−0.2391187060	H	2.7791843710	0.7739754269	0.6475928653
C	1.3698072872	0.3370933383	−0.7421517912	H	2.6670373001	1.8871017281	−0.6698120982
C	0.9557096103	−0.9885672284	−0.2104604083	H	0.9936987863	0.6683581846	−1.7051610406
C	−0.5413385959	−1.3230723432	−0.3860477942	H	1.2775275257	−1.1021122346	0.8318603448
C	−1.4872123669	−0.6112482306	0.5885553436	H	1.5389645164	−1.7159376336	−0.7982142158
C	−1.4033696629	0.8907766361	0.5209301377	H	−0.6571521414	−2.4007208105	−0.2445367855
O	−2.4196237716	1.5071653258	1.1112450424	H	−0.8420540903	−1.1149821838	−1.4193470181
O	−0.4773397830	1.5040702129	−0.0092898561	H	−2.5244215683	−0.9079272903	0.4031821996
				H	−1.2790722751	−0.9014230377	1.6276589191
				H	−2.3020920493	2.4731927949	1.0530148616

HMDB MoNA (+219.0 kJ/mol)

N	2.4052198785	0.7040132563	−0.9742533583	H	3.3157139363	0.9724927876	−1.3641058222
C	2.4453981942	0.5049071638	0.5565982580	H	2.0903425248	−0.1625107091	−1.4259162732
C	1.1160245766	0.1327296504	0.9804965980	H	1.7145518911	1.4262976956	−1.2094016253
C	−0.0460879593	−0.1642724366	1.1433771825	H	2.7926289622	1.4529896221	0.9778165581
C	−1.4512728969	−0.5188033106	1.3067287046	H	3.1991230361	−0.2656606254	0.7436213782
C	−2.1934148528	−0.5101356450	−0.0403123194	H	−1.9381161719	0.1941171283	1.9801861937
O	−2.1036519460	0.7984499055	−0.5653262533	H	−1.5332811463	−1.5183150508	1.7461220733
O	−1.5687174659	−1.4639738779	−0.8746306306	H	−3.2426111309	−0.7819576886	0.1333147637
				H	−2.8223079559	0.9596453019	−1.1913780068
				H	−2.1794414740	−1.7600131676	−1.5629374209

3.5.7 MS^3 fragment ion, m/z 115 (via fragment ion m/z 157)

This work (0.0 kJ/mol)

N	2.3432178570	−0.0293148693	0.2853270013	H	2.9177720632	−0.7631261762	0.6869468718
C	0.9125890138	−0.3200006798	0.3620400353	H	2.6635564580	0.1476745384	−0.6642673961
C	0.1961681895	−0.9525107649	−0.8399602037	H	0.7547706357	−0.9648420575	1.2382125346
C	−1.3084163337	−1.0219309724	−0.5297724181	H	0.5973240788	−1.9537986587	−1.0177252229
C	−1.9204746772	0.3628697655	−0.2927564962	H	0.3837237256	−0.3589030422	−1.7442422155
N	−1.0185462646	1.2408827083	0.4970975284	H	−1.8489458210	−1.4930731400	−1.3551467064
C	0.2423208972	1.0006755736	0.7260761524	H	−1.4704457666	−1.6535983749	0.3514608594
O	0.9858057651	1.9043280431	1.2849215207	H	−2.8667449370	0.2913095533	0.2484380659
				H	−2.1093404225	0.8824453877	−1.2383222576
				H	1.9164023532	1.5269837972	1.2232002840
				H	−1.3710368144	2.1539293690	0.7786720629

3.5.8 MS^n fragment ion, m/z 112

MS^n fragment ion, m/z 112 (structure A)

This work (0.0 kJ/mol)

N	2.1171448242	0.9740384210	−0.0332407306	H	3.1222160364	0.9699398925	−0.1248741158
C	1.4111410271	−0.1727783565	−0.0948353760	H	1.6390496515	−2.2128504445	−0.2963322297
N	2.0664322126	−1.3464507523	−0.0067633391	H	3.0273897632	−1.3832684393	0.2996261182
N	0.0887275353	−0.1545291942	−0.2414154905	H	−0.6915766608	−2.0218072282	−0.8832108588
C	−0.8029981900	−1.3346898865	−0.0372861233	H	−0.5266887912	−1.8471802406	0.8869919688
C	−2.2263615580	−0.7217555240	0.0019185987	H	−2.6523860243	−0.7459325463	1.0119394732
C	−2.0113210247	0.6977023471	−0.4616913602	H	−2.9136566948	−1.2690654005	−0.6492021692
C	−0.7127537037	0.9808645450	−0.5728588388	H	−2.8112438437	1.3963149866	−0.6694496943
				H	−0.2410896256	1.8956471178	−0.9030744408
				H	1.6803568705	1.8444021937	0.2297480143

This work (+8.4 kJ/mol)

N	2.5571179776	−0.6687788559	−0.4422075494	H	3.2392985130	0.0013747812	−0.7670397820
C	1.2923186292	−0.3001915739	−0.1498491630	H	1.8840866158	1.5198653228	0.4164815812
N	1.0593585090	0.9840216076	0.1764660546	H	0.0774772913	2.8097451129	0.1659611866
C	−0.1415884342	1.7507494058	0.1002784488	H	−2.1439103231	2.1195334177	−0.0960535610
C	−1.4026836399	1.3276706441	−0.0373240165	H	−2.8753956732	−0.1242203004	0.4505911018
C	−1.9217911081	−0.0791918515	−0.0890244293	H	−2.1475731741	−0.3678531990	−1.1235203298
C	−0.9708241061	−1.1026540094	0.5355863944	H	−0.7683371190	−0.8524956224	1.5813309364
N	0.3166650573	−1.2089472275	−0.1769427972	H	−1.4124490432	−2.1000918246	0.5142865747
				H	0.5144077778	−2.0726535893	−0.6652493116
				H	2.8439222497	−1.6358822382	−0.3938713386

This work (+27.2 kJ/mol)

N	2.4769940264	0.1820389216	−1.1272632118	H	2.8740649555	1.0919647258	−1.3217406050
C	1.3981860490	0.1213116777	−0.3403305813	H	−0.4150081487	2.7218599087	0.6128458207
N	0.9040004809	1.3482097858	0.0218997420	H	−2.2404528402	1.4075736928	−0.4928288836
C	−0.3067059310	1.6838351298	0.2884307611	H	−2.0704362916	1.0926992951	1.2030935218
C	−1.5826829956	0.9077521704	0.2339832887	H	−2.4387635880	−1.0597764802	0.2324008775
C	−1.4866847608	−0.5967241703	−0.0438140091	H	−1.3461090924	−0.7936475663	−1.1122923426
C	−0.3590727626	−1.2734412210	0.7434516424	H	−0.3285194132	−0.9261511453	1.7832622718
N	0.9566733062	−1.0503417086	0.1127963400	H	−0.4974044190	−2.3556666115	0.7657840086
				H	1.5384686944	−1.8659030030	−0.0414607216
				H	2.9235527308	−0.6355934014	−1.5182179196

mzCloud (+61.2 kJ/mol)

N	−2.1505379570	−0.4613182687	−1.2559545455	H	−3.1668524024	−0.4815544327	−1.3360325981
C	−1.7321227297	−0.0956384828	−0.1194809328	H	−2.0584013410	0.9738025105	1.6128654926
N	−2.3687998444	0.1754192288	1.0703655710	H	−3.3769740357	0.0792751710	1.0558377920
N	−0.2746473001	−0.0397636374	−0.0104205994	H	−0.0753987014	−0.4211215492	2.0125404413
C	0.4190349378	−0.2304616948	1.0638351967	H	2.4163373087	0.3809133770	1.5543850263
C	1.8818886217	−0.2216948746	0.8120710542	H	2.2506047099	−1.2516684899	0.9387665321
C	1.9888998485	0.2959601143	−0.6452876588	H	2.2518457599	1.3566556784	−0.6467346243
C	0.5812808812	0.0905710387	−1.2371055802	H	2.7474932411	−0.2369623636	−1.2194921394
				H	0.2073880160	0.9185045476	−1.8394200358
				H	0.4588609868	−0.8309178724	−1.8107383917

Zhang et al. (+106.8 kJ/mol)

N	2.7832659972	−0.1880833859	0.7169668085	H	3.1910646955	−1.1068609701	0.8855107324
C	1.5807988158	−0.2035077771	0.3142126636	H	1.8513592923	1.7960106874	−0.1063160655
N	1.0774578939	1.1520493546	0.0681448290	H	−0.2208212772	2.6911577331	0.0327942344
C	−0.1239494059	1.6189566740	0.1999172374	H	−1.0662514374	0.3724875043	1.5750269247
C	−1.3034208227	0.8062146975	0.5934075085	H	−2.1613414047	1.4676388768	0.7238516615
C	−1.6530373378	−0.3548599541	−0.3823654251	H	−2.2971557294	−1.0449438044	0.1688139637
C	−0.4056069105	−1.0732569114	−0.9172604790	H	−2.2346573889	0.0263886747	−1.2269860911
N	0.6614652723	−1.1908736767	0.0960336222	H	−0.6646583162	−2.0846945784	−1.2350546377
				H	−0.0132833947	−0.5603148441	−1.8028773728
				H	0.9988714584	−2.1237083003	0.2962798852

MS^n fragment ion, m/z 112 (structure B)

This work, mzCloud (+61.2 kJ/mol)

N	−2.1505379570	−0.4613182687	−1.2559545455	H	−3.1668524024	−0.4815544327	−1.3360325981
C	−1.7321227297	−0.0956384828	−0.1194809328	H	−2.0584013410	0.9738025105	1.6128654926
N	−2.3687998444	0.1754192288	1.0703655710	H	−3.3769740357	0.0792751710	1.0558377920
N	−0.2746473001	−0.0397636374	−0.0104205994	H	−0.0753987014	−0.4211215492	2.0125404413
C	0.4190349378	−0.2304616948	1.0638351967	H	2.4163373087	0.3809133770	1.5543850263
C	1.8818886217	−0.2216948746	0.8120710542	H	2.2506047099	−1.2516684899	0.9387665321
C	1.9888998485	0.2959601143	−0.6452876588	H	2.2518457599	1.3566556784	−0.6467346243
C	0.5812808812	0.0905710387	−1.2371055802	H	2.7474932411	−0.2369623636	−1.2194921394
				H	0.2073880160	0.9185045476	−1.8394200358
				H	0.4588609868	−0.8309178724	−1.8107383917

This work, mzCloud (+61.6 kJ/mol)

N	−2.0814941128	−1.0504273306	1.2158699997	H	−3.0909541737	−1.1331187008	1.3247991787
C	−1.7197854604	−0.2479548894	0.3057556542	H	−3.4221286325	0.3676931475	−0.6275569293
N	−2.4134197502	0.4349543572	−0.6520259170	H	−2.0559899709	1.2943956256	−1.0456306935
N	−0.2644503705	−0.0683568502	0.1832522319	H	0.1362346353	−0.6383465380	2.1163248532
C	0.5440112524	−0.3207282602	1.1601762857	H	2.5821452368	−0.9612201997	1.1382092712
C	1.9665916195	−0.1228110140	0.7944012307	H	2.3421094048	0.7632448538	1.3288733582
C	1.9222340754	0.0520838470	−0.7456295005	H	2.5910459503	0.8386460176	−1.0966358098
C	0.4463298966	0.3787207328	−1.0584231259	H	2.2124244649	−0.8791825987	−1.2374295869
				H	0.2678216888	1.4512217331	−1.1892983811
				H	0.0374742462	−0.1589139330	−1.9150321195

Zhang et al. (+106.8 kJ/mol)

N	2.7832659972	−0.1880833859	0.7169668085	H	3.1910646955	−1.1068609701	0.8855107324
C	1.5807988158	−0.2035077771	0.3142126636	H	1.8513592923	1.7960106874	−0.1063160655
N	1.0774578939	1.1520493546	0.0681448290	H	−0.2208212772	2.6911577331	0.0327942344
C	−0.1239494059	1.6189566740	0.1999172374	H	−1.0662514374	0.3724875043	1.5750269247
C	−1.3034208227	0.8062146975	0.5934075085	H	−2.1613414047	1.4676388768	0.7238516615
C	−1.6530373378	−0.3548599541	−0.3823654251	H	−2.2971557294	−1.0449438044	0.1688139637
C	−0.4056069105	−1.0732569114	−0.9172604790	H	−2.2346573889	0.0263886747	−1.2269860911
N	0.6614652723	−1.1908736767	0.0960336222	H	−0.6646583162	−2.0846945784	−1.2350546377
				H	−0.0132833947	−0.5603148441	−1.8028773728
				H	0.9988714584	−2.1237083003	0.2962798852

4 Proposed fragmentation mechanisms

4.1 α-Amino-adipic acid

4.1.1 m/z 162 → 144

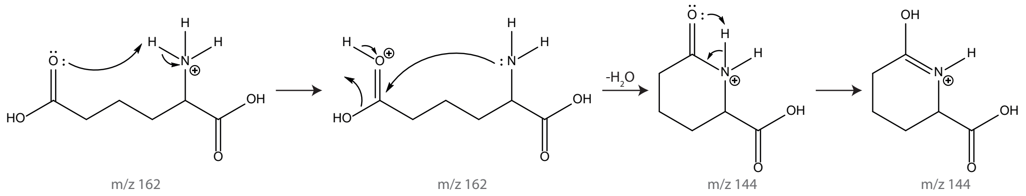

Figure S36: Suggested reaction pathway based on precursor and product ion structure.

4.1.2 m/z 162 → 116

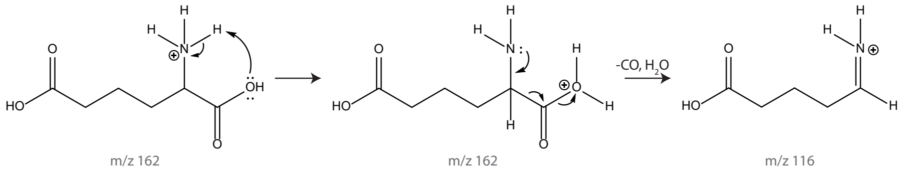

Figure S37: Suggested reaction pathway based on precursor and product ion structure.

4.1.3 m/z 162 → 98

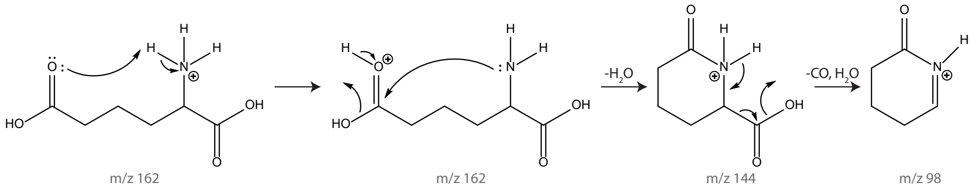

Figure S38: Suggested reaction pathway based on precursor and product ion structure.

4.2 Urocanic acid

4.2.1 m/z 139 → 121

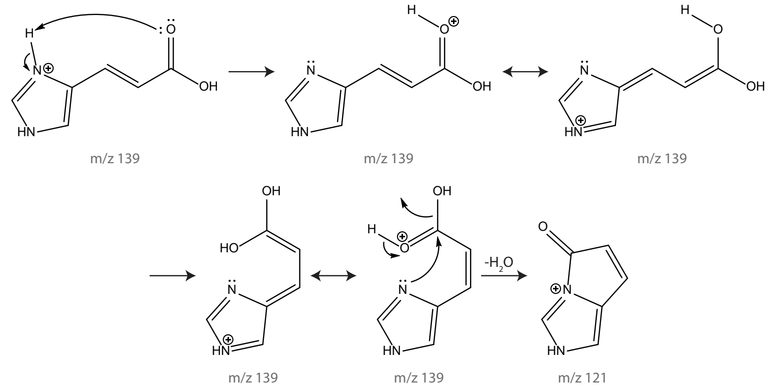

Figure S39: Suggested reaction pathway based on precursor and product ion structure.

4.3 Citrulline

4.3.1 m/z 176 → 159

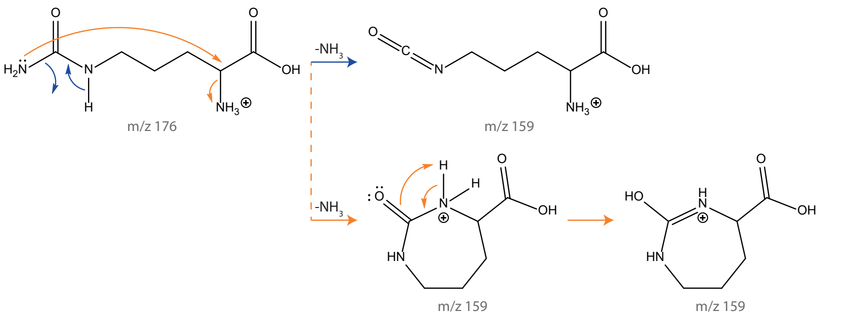

Figure S40: Suggested reaction pathway based on precursor and product ion structure.

4.3.2 m/z 176 → 158

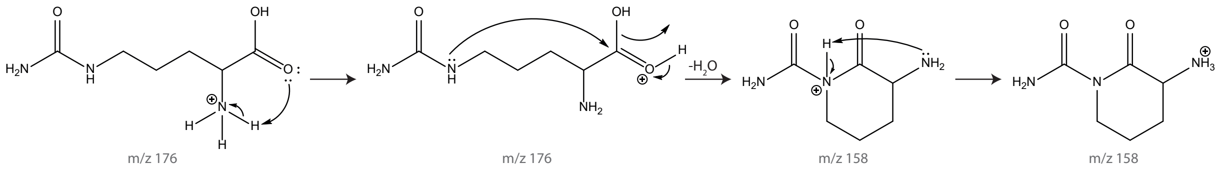

Figure S41: Suggested reaction pathway based on precursor and product ion structure.

4.3.3 m/z 176 → 116

Structure I

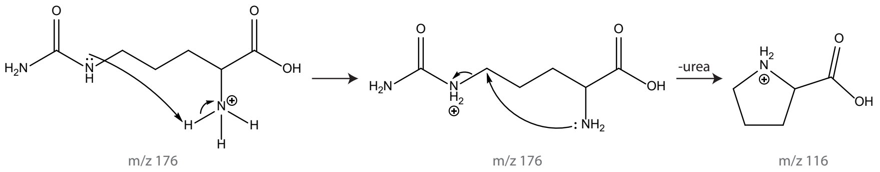

Figure S42: Suggested reaction pathway based on precursor and product ion structure.

Structure II

No proposed reaction mechanisms.

4.3.4 m/z 176 → 159 → 115

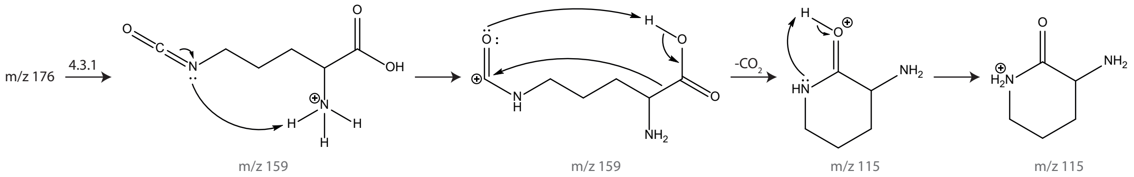

Figure S43: Suggested reaction pathway based on precursor and product ion structure.

4.3.5 m/z 176 → 158 → 115

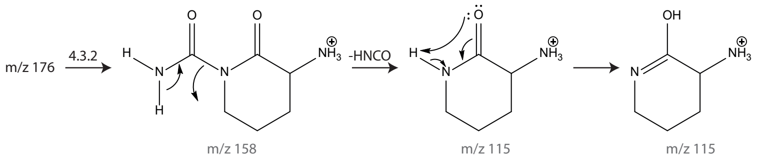

Figure S44: Suggested reaction pathway based on precursor and product ion structure.

4.3.6 m/z 176 → 159 → 113

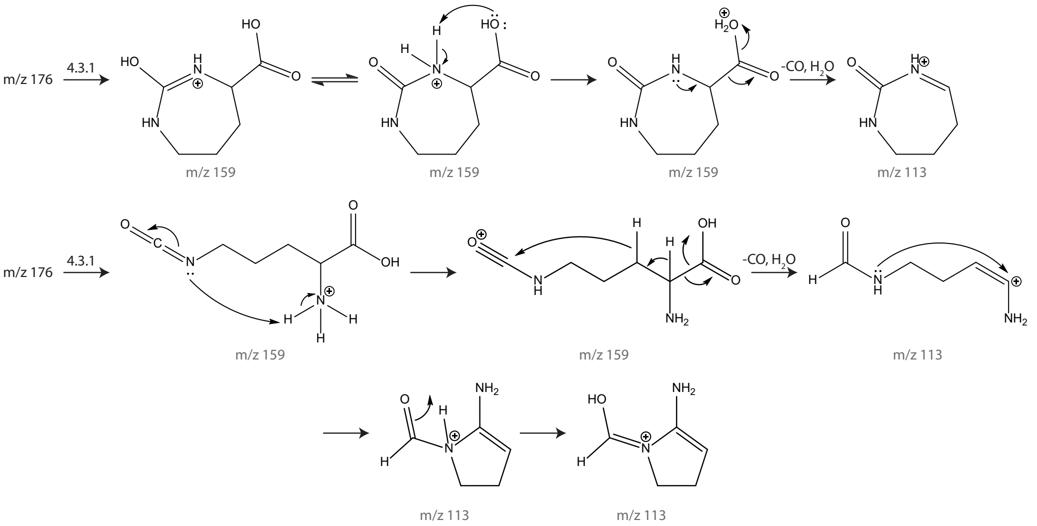

Figure S45: Suggested reaction pathway based on precursor and product ion structure.

4.4 Homocitrulline

4.4.1 m/z 190 → 173

No proposed reaction mechanism.

4.4.2 m/z 190 → 147

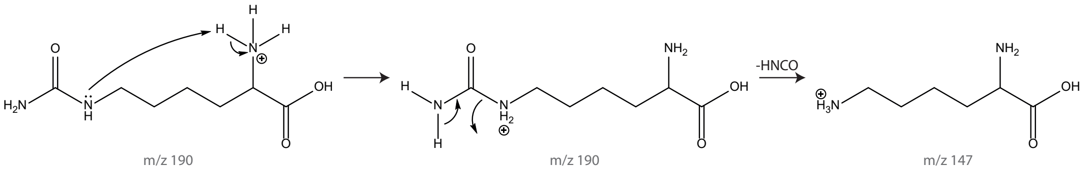

Figure S46: Suggested reaction pathway based on precursor and product ion structure.

4.4.3 m/z 190 → 144

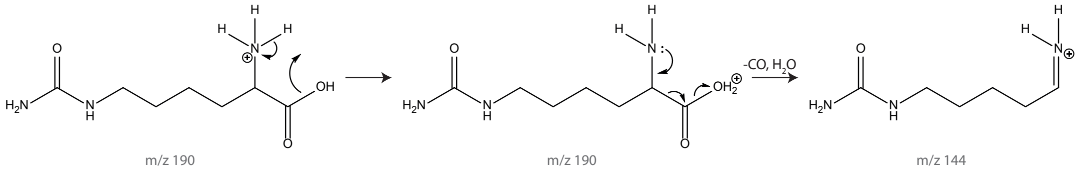

Figure S47: Suggested reaction pathway based on precursor and product ion structure.

4.4.4 m/z 190 → 147 → 130 & m/z 190 → 130 (via fragment m/z 147)

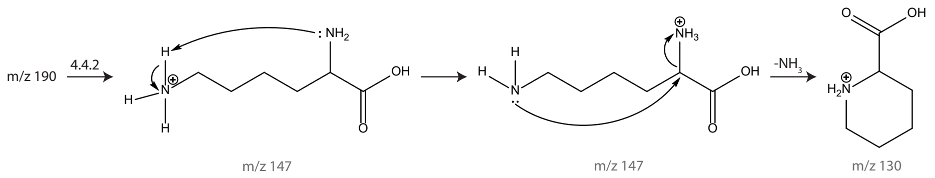

Figure S48: Suggested reaction pathway based on precursor and product ion structure.

4.4.5 m/z 190 → 144 → 127 & m/z 190 → 127 (via fragment m/z 144)

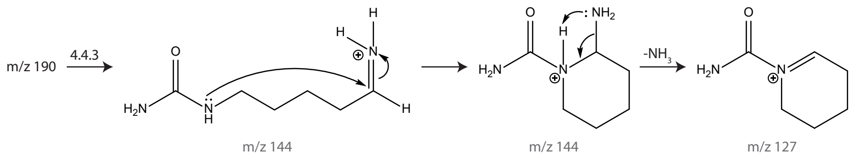

Figure S49: Suggested reaction pathway based on precursor and product ion structure.

4.4.6 m/z 190 → 173 → 127

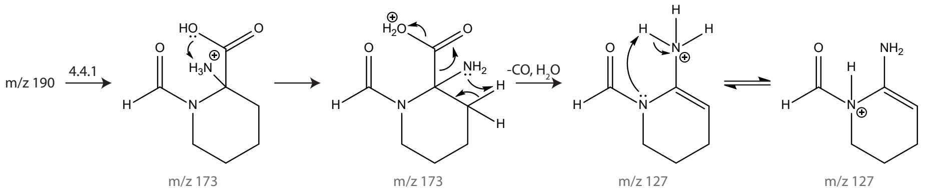

Figure S50: Suggested reaction pathway based on precursor and product ion structure.

4.4.7 m/z 190 → 173 → 127 → 84

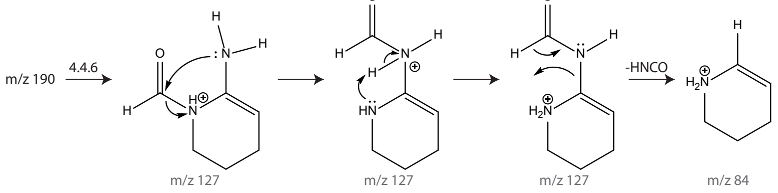

Figure S51: Suggested reaction pathway based on precursor and product ion structure.

4.4.8 m/z 190 → 173 → 84

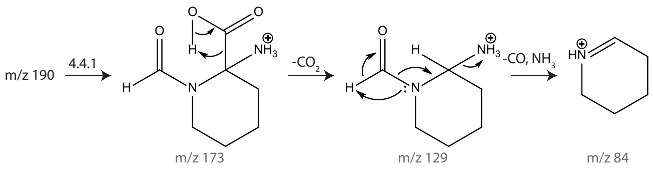

Figure S52: Suggested reaction pathway based on precursor and product ion structure.

4.4.9 m/z 190 → 144 → 127 → 84 & m/z 190 → 84 (via fragment m/z 127)

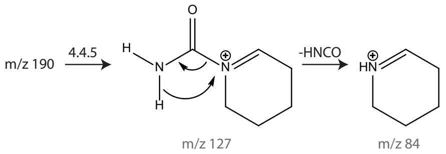

Figure S53: Suggested reaction pathway based on precursor and product ion structure.

4.5 Arginine

4.5.1 m/z 175 → 158

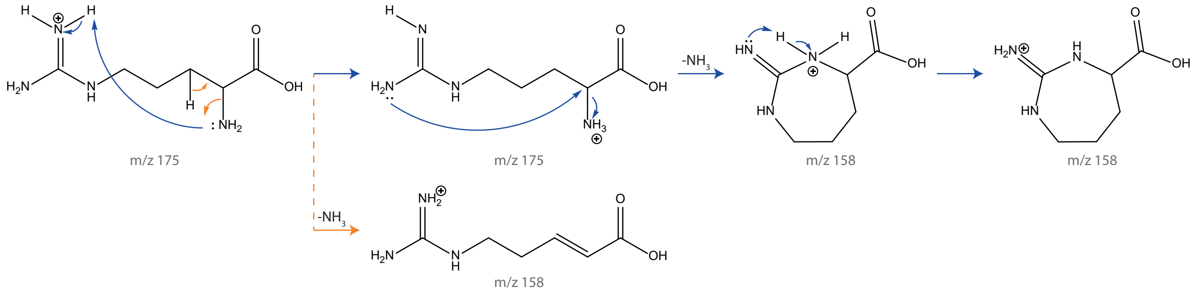

Figure S54: Suggested reaction pathway based on precursor and product ion structure.

4.5.2 m/z 175 → 157

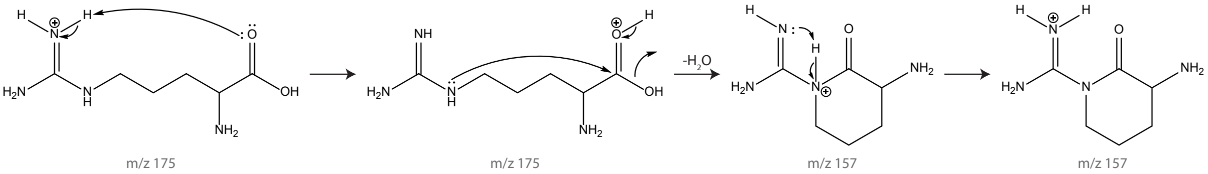

Figure S55: Suggested reaction pathway based on precursor and product ion structure.

4.5.3 m/z 175 → 130

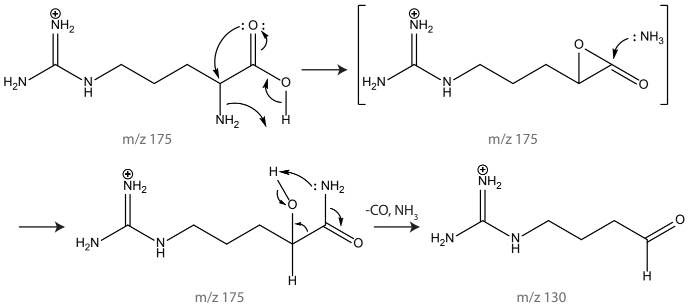

Figure S56: Suggested reaction pathway based on precursor and product ion structure, taken from Cai *et al.* [2].

4.5.4 m/z 175 → 116

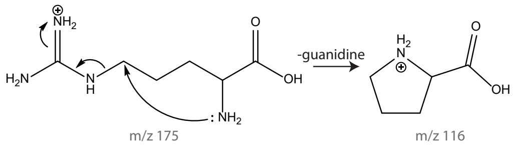

Figure S57: Suggested reaction pathway based on precursor and product ion structure, taken from Csonka *et al.* [3].

4.5.5 m/z 175 → 158 → 116

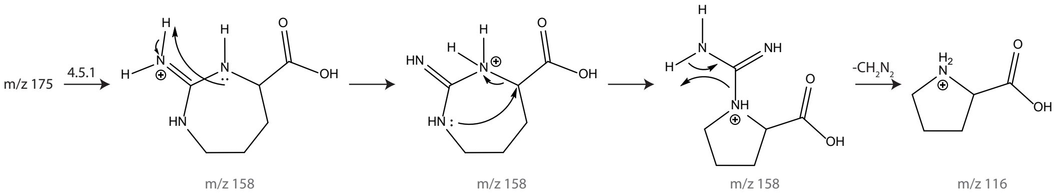

Figure S58: Suggested reaction pathway based on precursor and product ion structure.

4.5.6 m/z 175 → 157 → 115

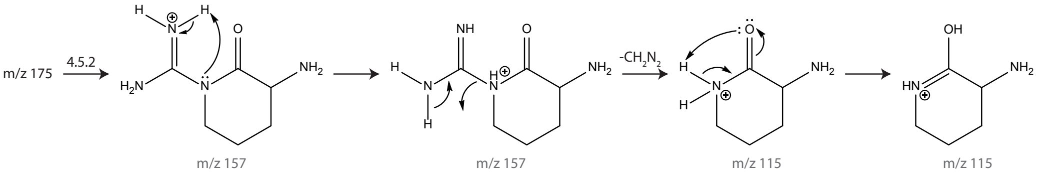

Figure S59: Suggested reaction pathway based on precursor and product ion structure.

4.5.7 m/z 175 → 158 → 112 and m/z 175 → 112 (via fragment m/z 158)

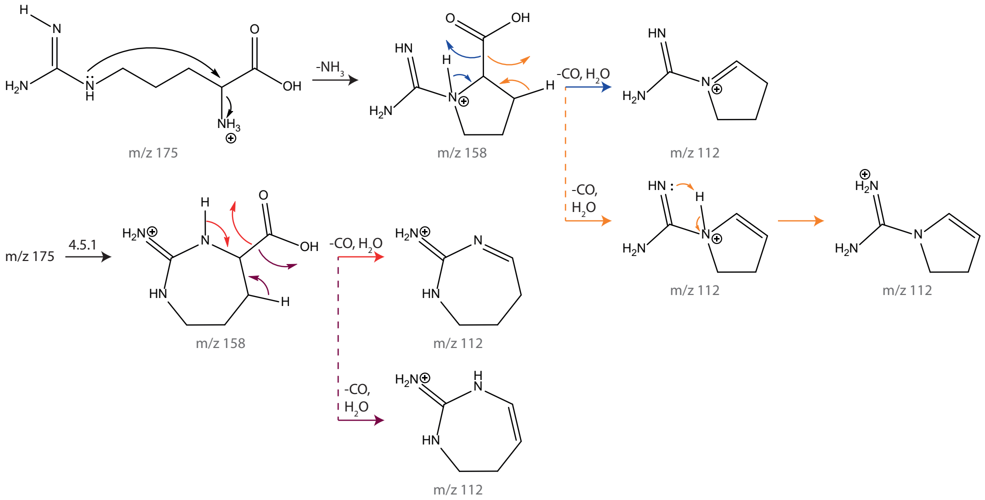

Figure S60: Suggested reaction pathway based on precursor and product ion structure.

4.5.8 m/z 175 → 157 → 112

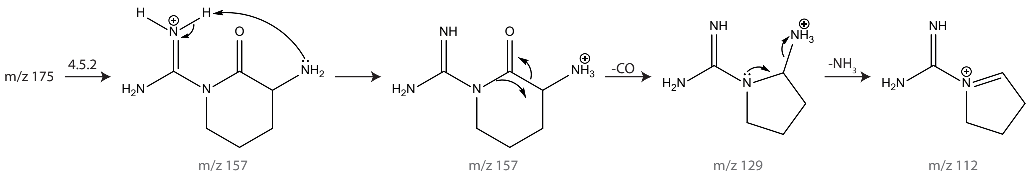

Figure S61: Suggested reaction pathway based on precursor and product ion structure.

5 Recorded MS/MS Spectra

High-resolution MS/MS spectra were obtained on a Bruker SolariX FTICR-MS using an FID transient length of 0.2097 s (α-amino-adipic acid and homocitrulline) or 0.4194 s (urocanic acid, citrulline and arginine).

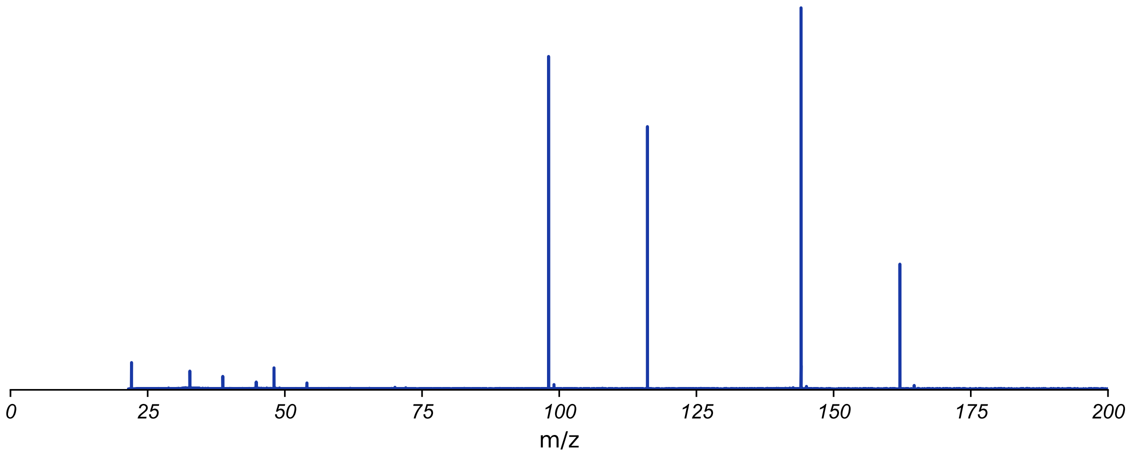

Figure S62: High-resolution MS/MS spectrum of protonated α-amino-adipic acid using a collision energy (CID) of 7.5 V.

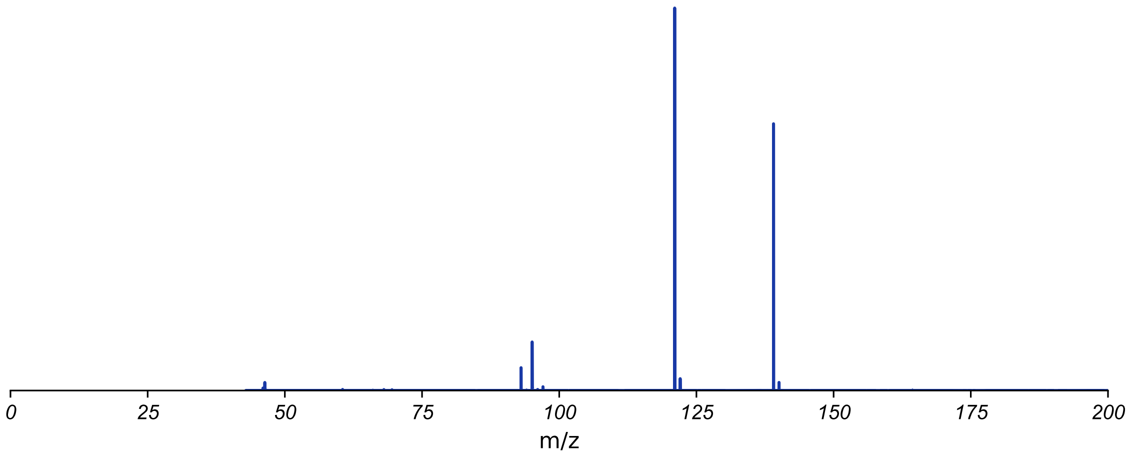

Figure S63: High-resolution MS/MS spectrum of protonated urocanic acid using a collision energy (CID) of 10 V.

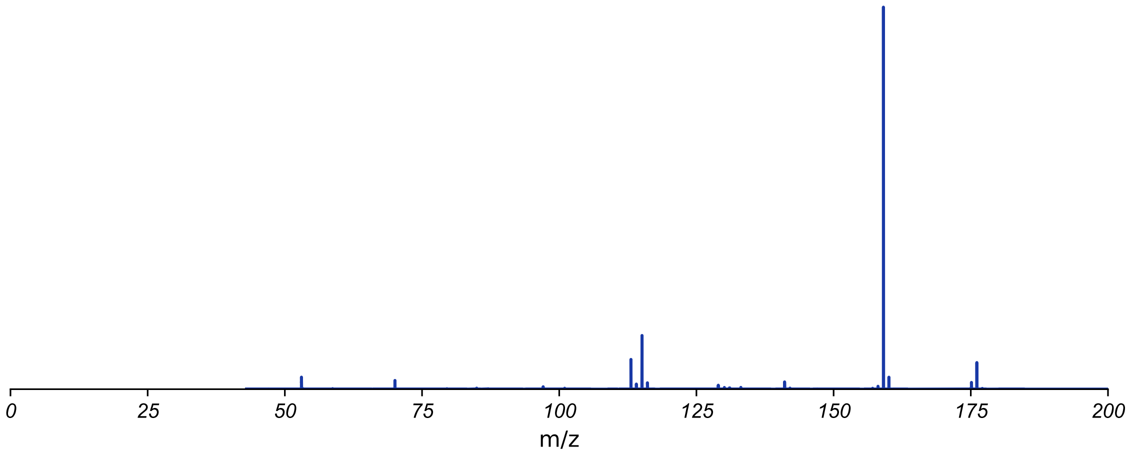

Figure S64: High-resolution MS/MS spectrum of protonated citrulline using a collision energy (CID) of 7.5 V.

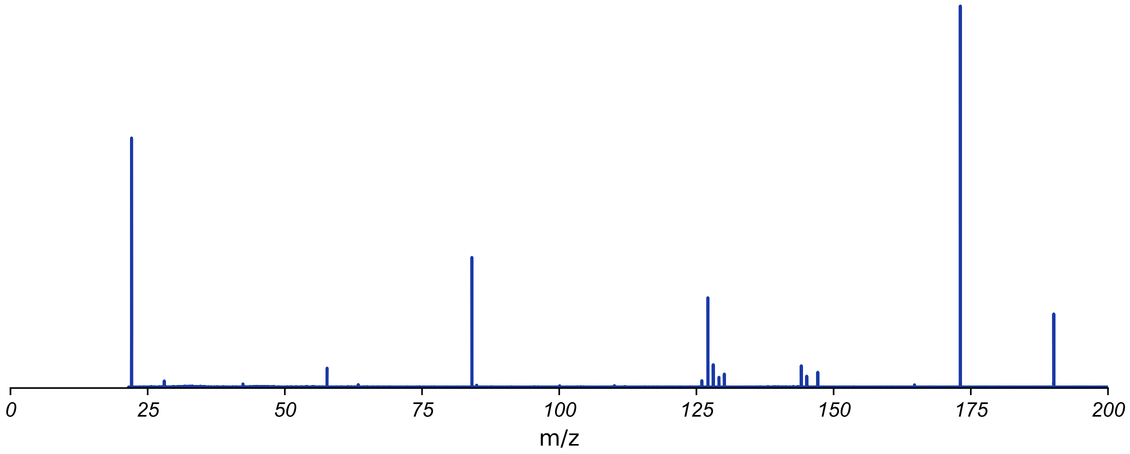

Figure S65: High-resolution MS/MS spectrum of protonated homocitrulline using a collision energy (CID) of 7.5 V.

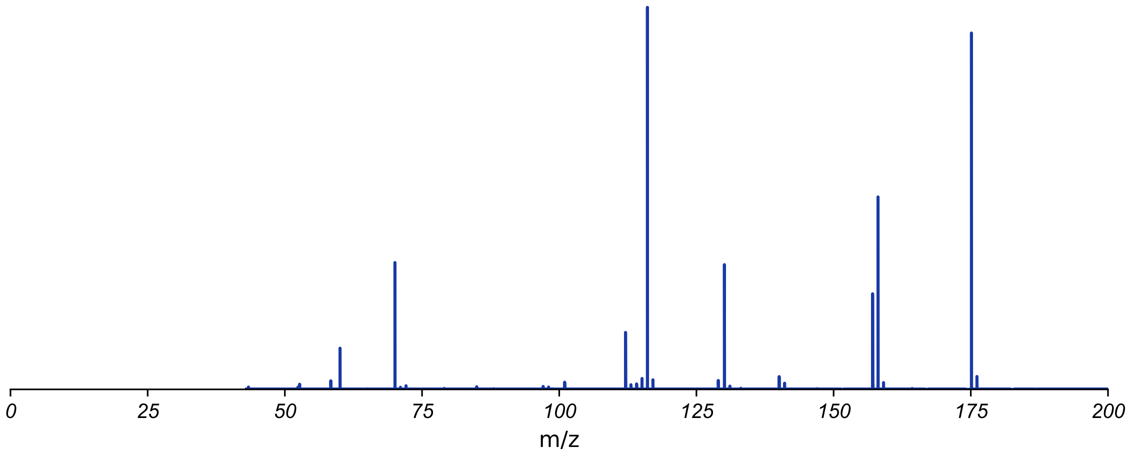

Figure S66: High-resolution MS/MS spectrum of protonated arginine using a collision energy (CID) of 7.5 V.

6 Accessed HMDB Spectra

The HMDB contains multiple MS/MS spectra per metabolite taken from different sources and at various CID energies. The table below identifies the spectra accessed in this study by their splash key.

Table S6: Overview of assessed MS/MS spectra in the HMDB.

Chemical	Source	Splash Key
α-Amino-adipic acid	Wishart Lab	splash10-00kg-2900000000-3e45ac769d3b229221a3
Urocanic acid	MoNA	splash10-00di-3900000000-8c1c060d06c1ce3fb5f7
	Wishart Lab	splash10-0079-1900000000-dac4165382f541a26a03
Citrulline	MoNA	splash10-0a4i-0900000000-4c1d7af748a47e489949
Homocitrulline	MoNa	splash10-00di-1900000000-adcca2902e0538d46b16
	Wishart Lab	splash10-006y-0900000000-8e7e33c59412f835b2c5
Arginine	MoNA	splash10-00di-3900000000-8c82418f7b35a97fb9b3

7 Additional molecules

Additional spectroscopically established product ion structures are collected here from previous studies. This data set, containing many peptide sequence ions, is contrasted against entries in the MS/MS libraries.

7.1 2-hydroxynicotinic acid

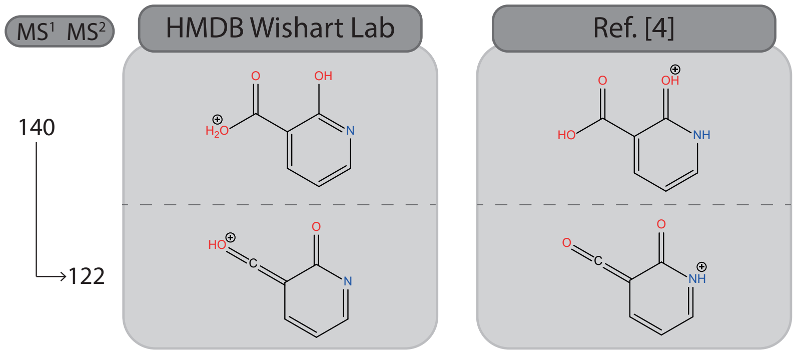

Figure S67: Spectral tree for protonated 2-hydroxynicotinic acid with structural annotations given in the HMDB library and structures identified through ion spectroscopy in a previous study [4].

7.2 Alanylasparagine (AlaAsn)

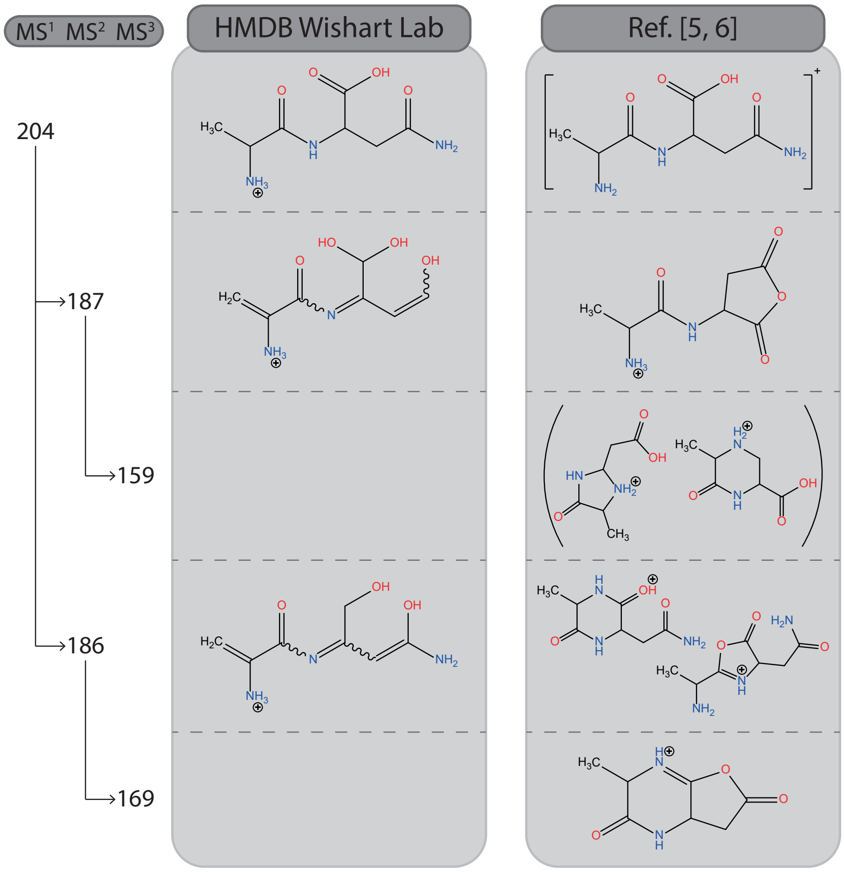

Figure S68: Spectral tree for protonated alanylasparagine with structural annotations given in the HMDB library and structures identified through ion spectroscopy in a previous study [5, 6].

7.3 Alanylglutamine (AlaGln)

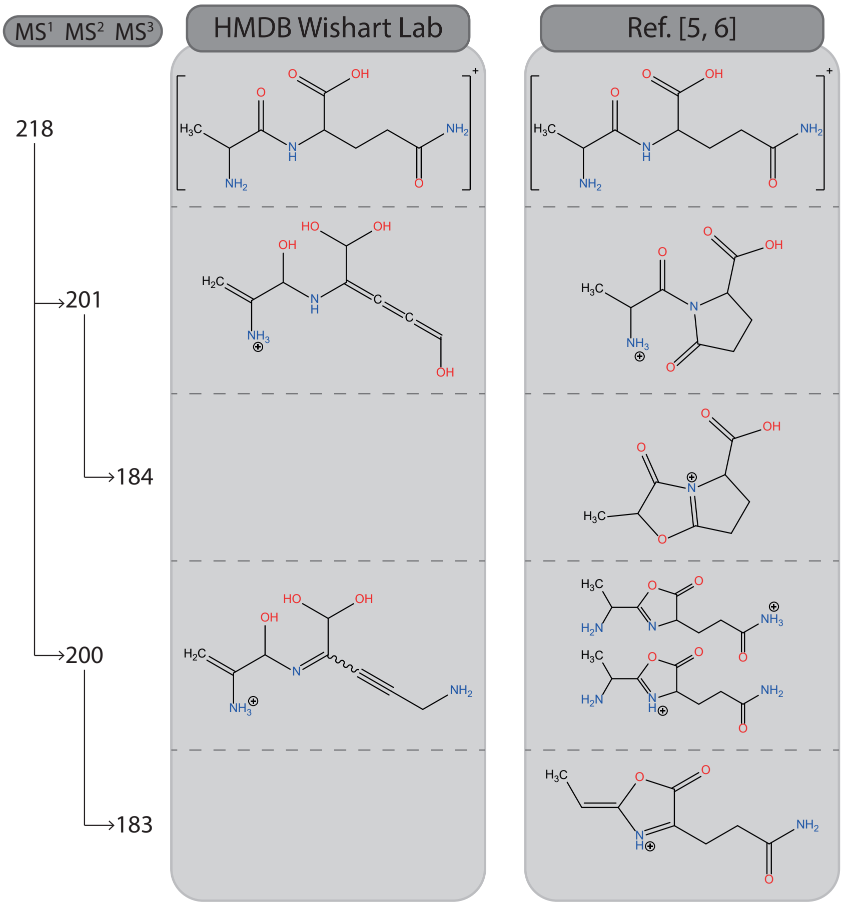

Figure S69: Spectral tree for protonated alanylglutamine with structural annotations given in the HMDB library and structures identified through ion spectroscopy in a previous study [5, 6].

7.4 Alanylserine (AlaSer)

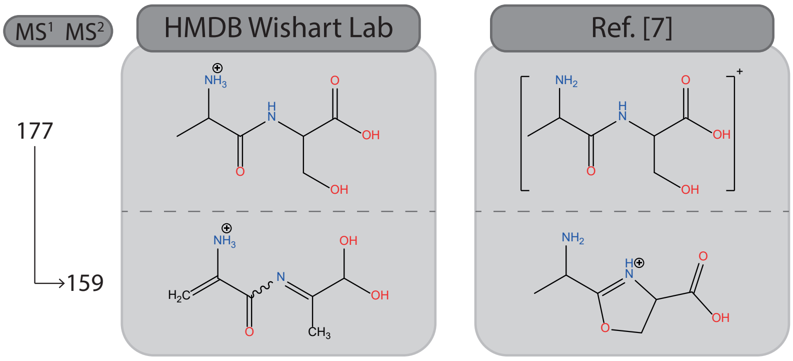

Figure S70: Spectral tree for protonated alanylserine with structural annotations given in the HMDB library and structures identified through ion spectroscopy in a previous study [7].

7.5 Alanylthreonine (AlaThr)

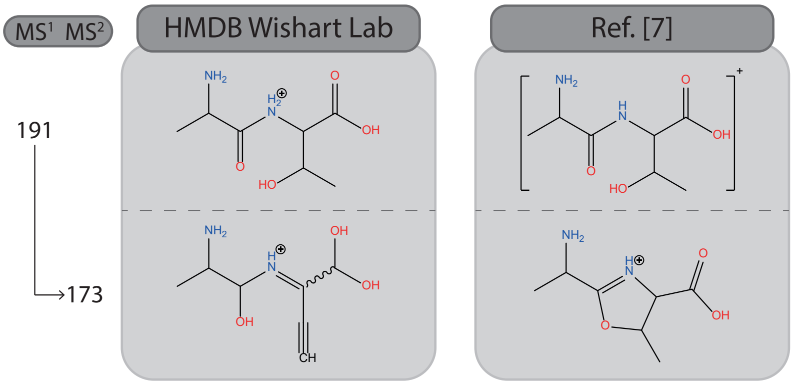

Figure S71: Spectral tree for protonated alanylthreonine with structural annotations given in the HMDB library and structures identified through ion spectroscopy in a previous study [7].

7.6 Arginylglycine (ArgGly)

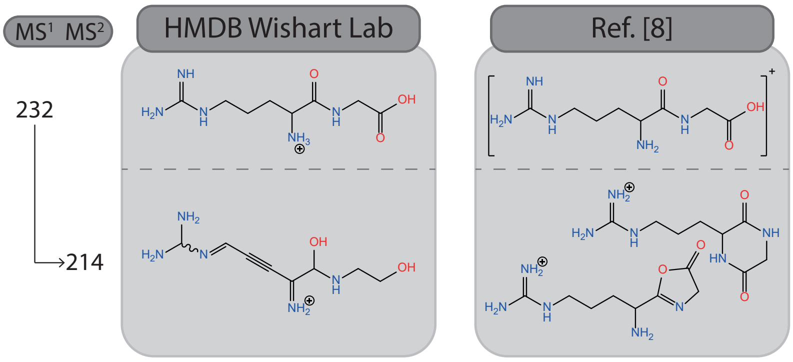

Figure S72: Spectral tree for protonated arginylglycine with structural annotations given in the HMDB library and structures identified through ion spectroscopy in a previous study [8].

7.7 Asparagine

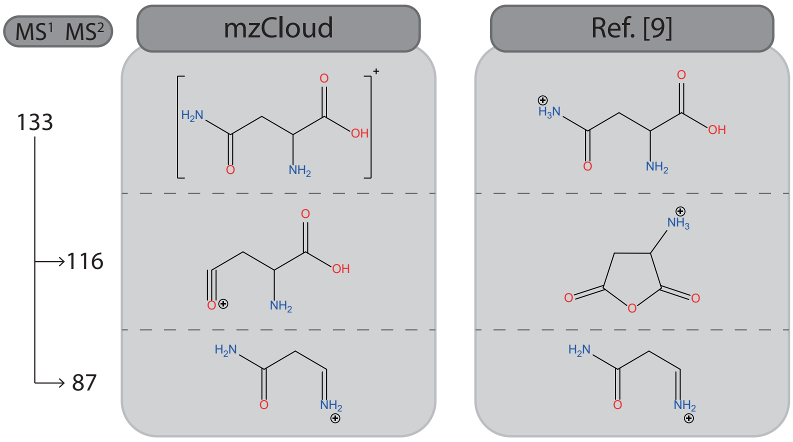

Figure S73: Spectral tree for protonated asparagine with structural annotations given in mzCloud and structures identified through ion spectroscopy in a previous study [9].

7.8 Asparaginyl-alanine (AsnAla)

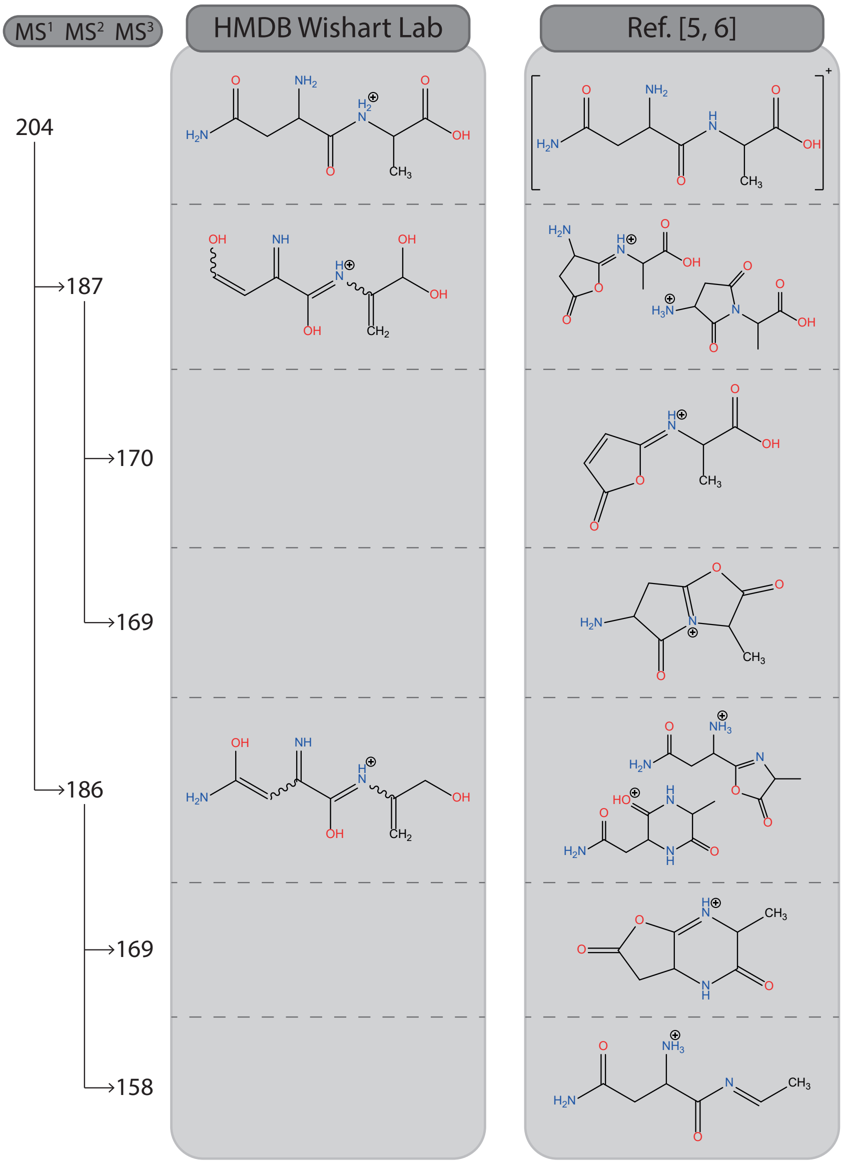

Figure S74: Spectral tree for protonated asparaginyl-alanine with structural annotations given in the HMDB library and structures identified through ion spectroscopy in a previous study [5, 6].

7.9 Asparaginyl-serine (AsnSer)

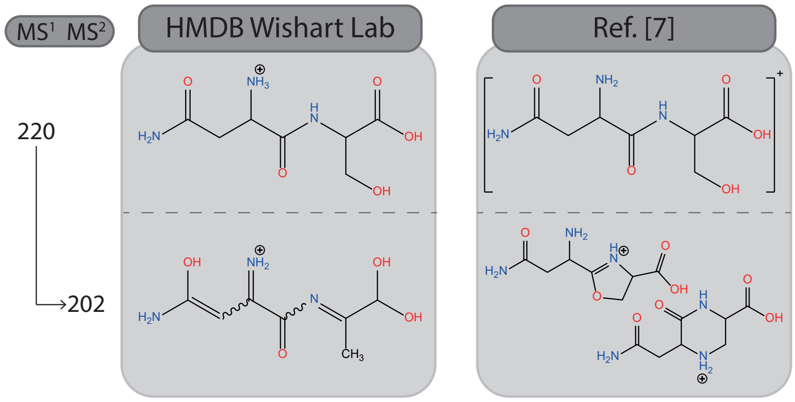

Figure S75: Spectral tree for protonated asparaginyl-serine with structural annotations given in the HMDB library and structures identified through ion spectroscopy in a previous study [7].

7.10 Asparaginyl-threonine (AsnThr)

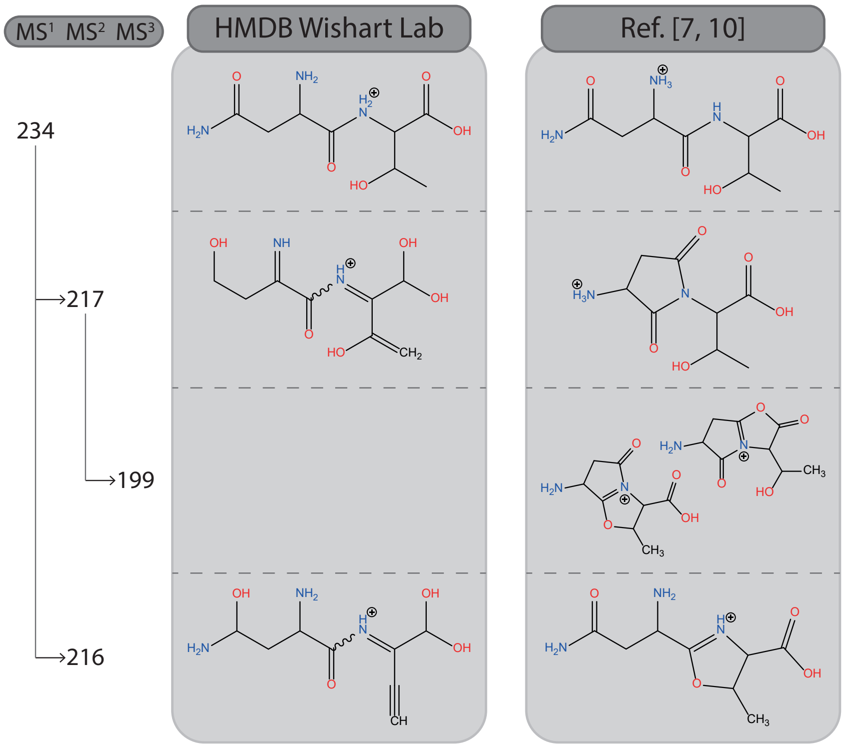

Figure S76: Spectral tree for protonated asparaginyl-threonine with structural annotations given in the HMDB library and structures identified through ion spectroscopy in a previous study [7, 10].

7.11 Asparaginyl-valine (AsnVal)

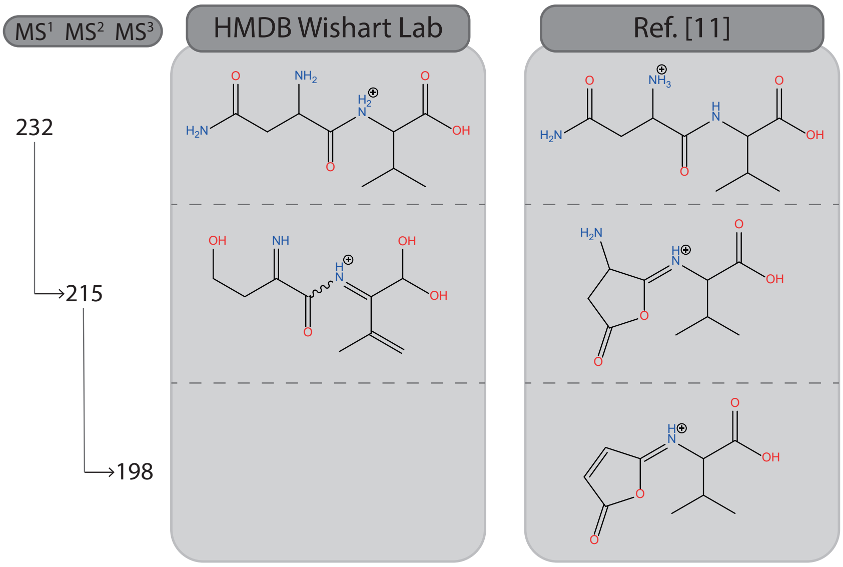

Figure S77: Spectral tree for protonated asparaginyl-valine with structural annotations given in the HMDB library and structures identified through ion spectroscopy in a previous study [11].

7.12 Aspartic acid

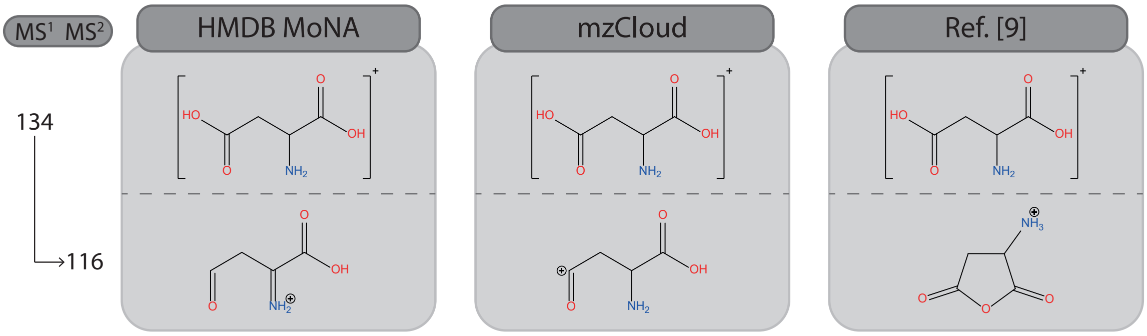

Figure S78: Spectral tree for protonated aspartic acid with structural annotations given in the HMDB library, mzCloud and structures identified through ion spectroscopy in a previous study [9].

7.13 Glutamic acid

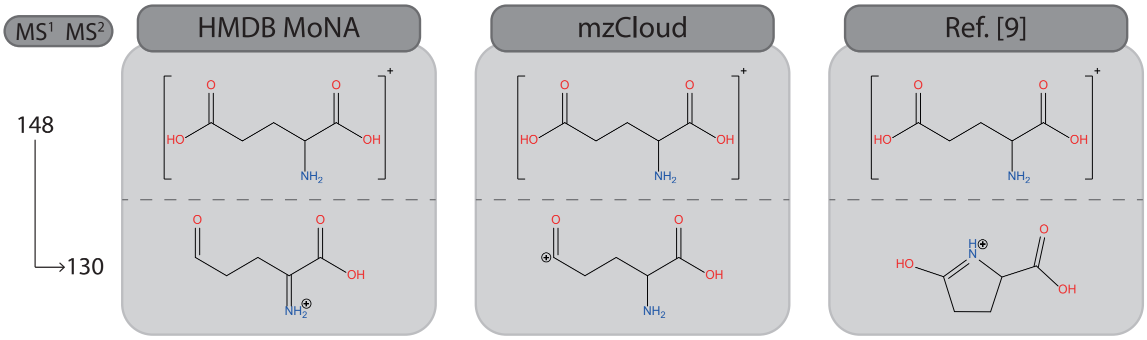

Figure S79: Spectral tree for protonated glutamic acid with structural annotations given in the HMDB library, mzCloud and structures identified through ion spectroscopy in a previous study [9].

7.14 Glutamine

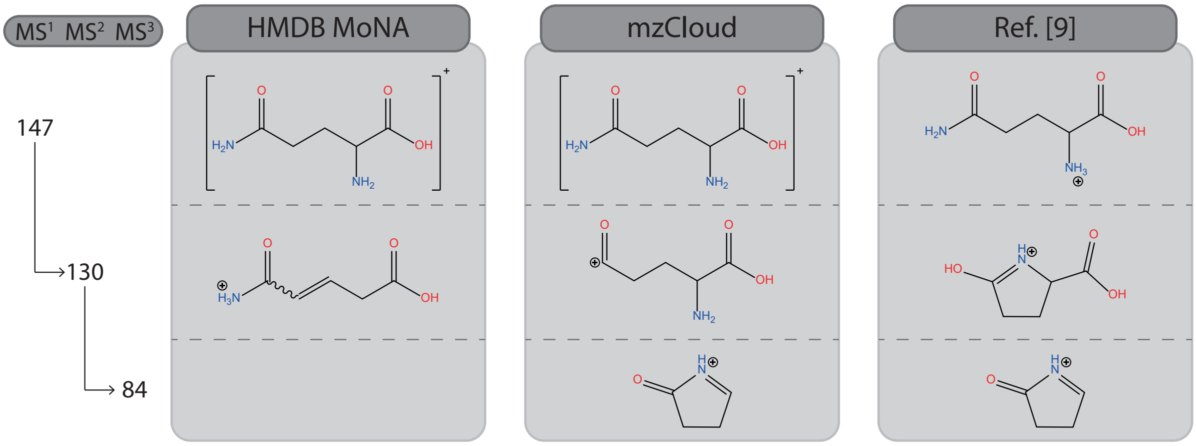

Figure S80: Spectral tree for protonated glutamine with structural annotations given in the HMDB library, mzCloud and structures identified through ion spectroscopy in a previous study [9].

7.15 Glutaminylalanine (GlnAla)

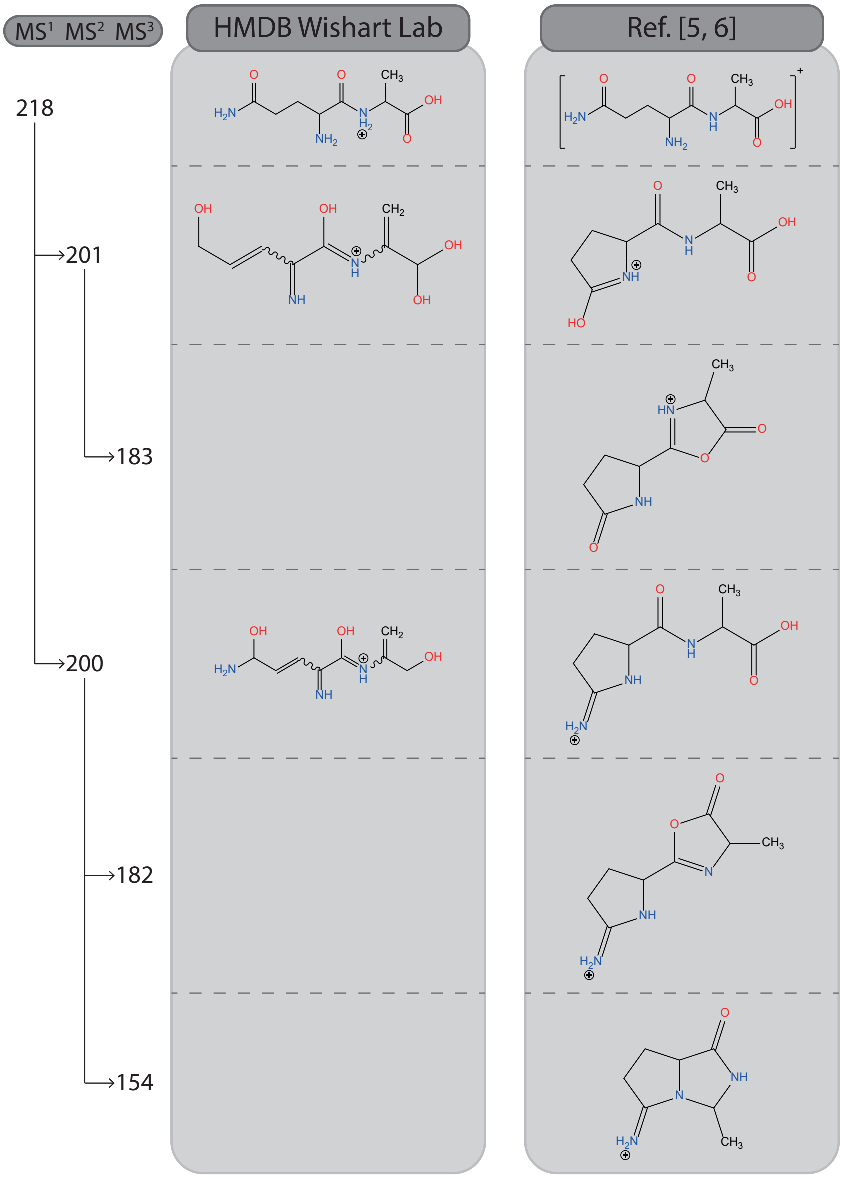

Figure S81: Spectral tree for protonated glutaminylalanine with structural annotations given in the HMDB library and structures identified through ion spectroscopy in a previous study [5, 6].

7.16 Glycyl-arginine (GlyArg)

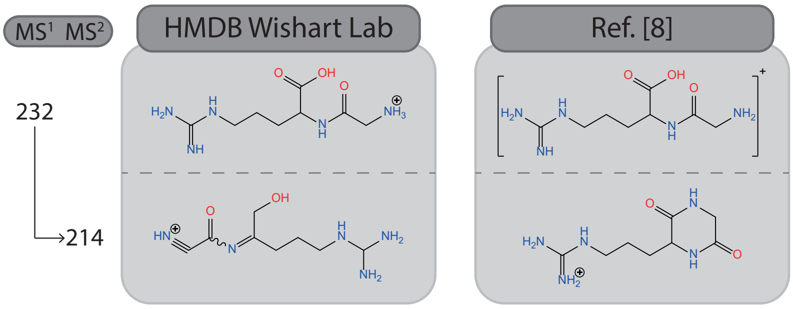

Figure S82: Spectral tree for protonated glycyl-arginine with structural annotations given in the HMDB library and structures identified through ion spectroscopy in a previous study [8].

7.17 Glycyl-serine (GlySer)

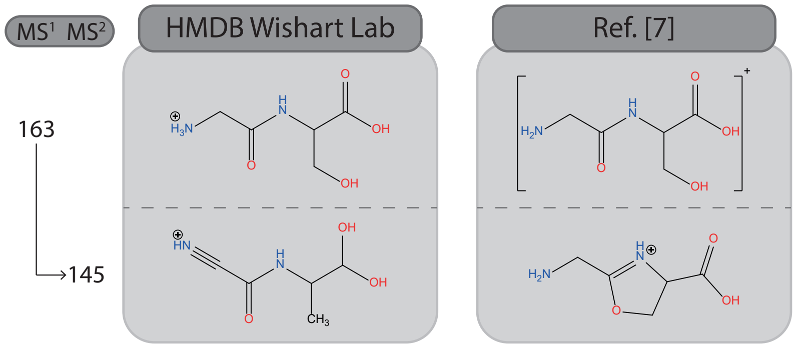

Figure S83: Spectral tree for protonated glycyl-serine with structural annotations given in the HMDB library and structures identified through ion spectroscopy in a previous study [7].

7.18 Glycyl-threonine (GlyThr)

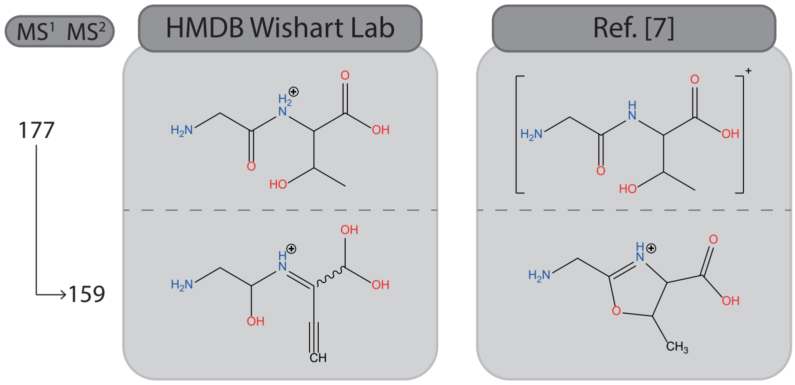

Figure S84: Spectral tree for protonated glycyl-threonine with structural annotations given in the HMDB library and structures identified through ion spectroscopy in a previous study [7].

7.19 Leu-enkephelin

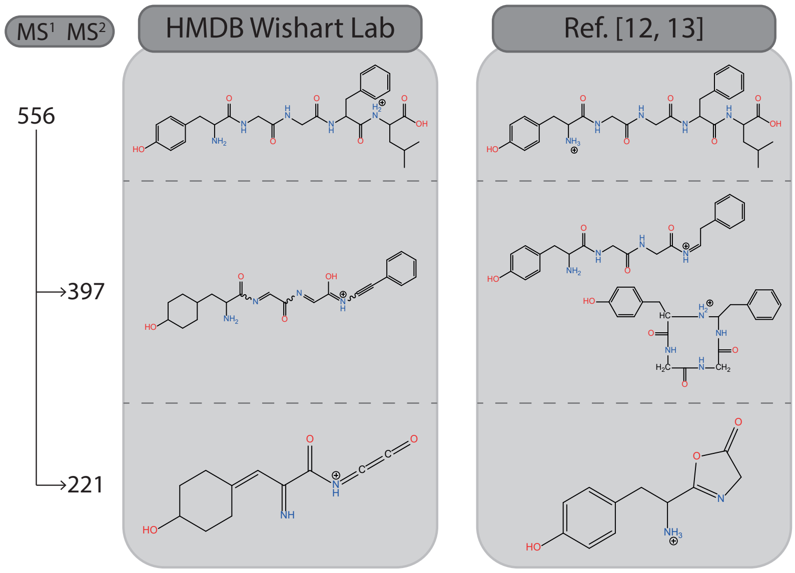

Figure S85: Spectral tree for protonated leu-enkephelin with structural annotations given in the HMDB library and structures identified through ion spectroscopy in a previous study [12, 13].

7.20 Prolyl-serine (ProSer)

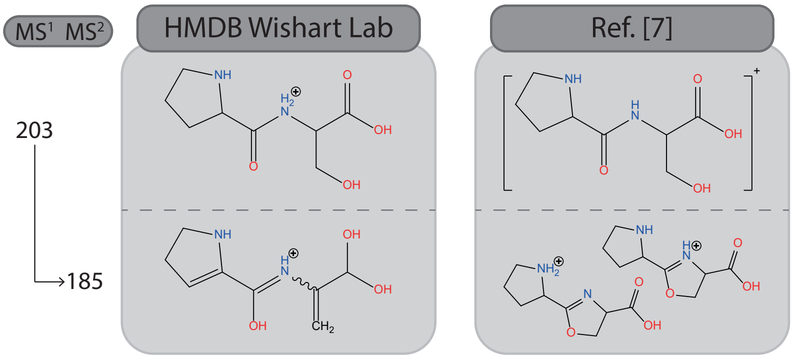

Figure S86: Spectral tree for protonated prolyl-serine with structural annotations given in the HMDB library and structures identified through ion spectroscopy in a previous study [7].

7.21 Prolyl-threonine (ProThr)

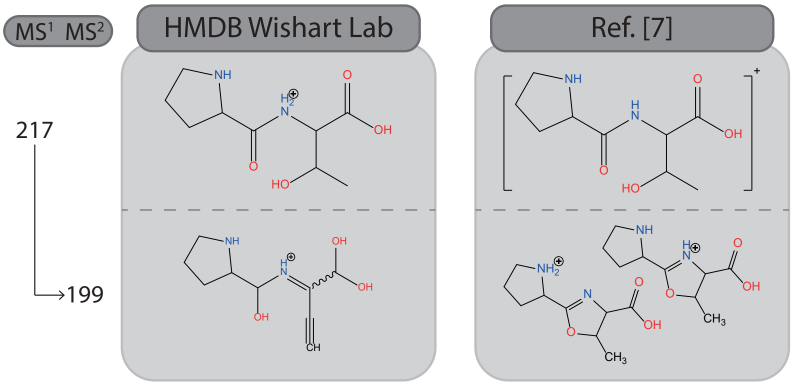

Figure S87: Spectral tree for protonated prolyl-threonine with structural annotations given in the HMDB library and structures identified through ion spectroscopy in a previous study [7].

7.22 Testosterone

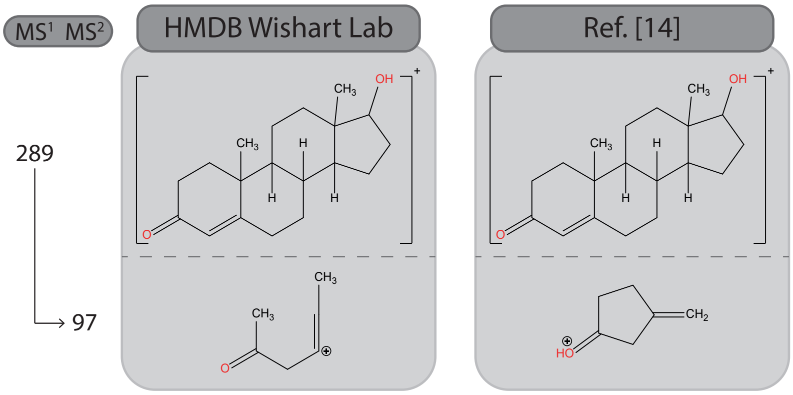

Figure S88: Spectral tree for protonated testosterone with structural annotations given in the HMDB library and structures identified through ion spectroscopy in a previous study [14].

7.23 Triglycine

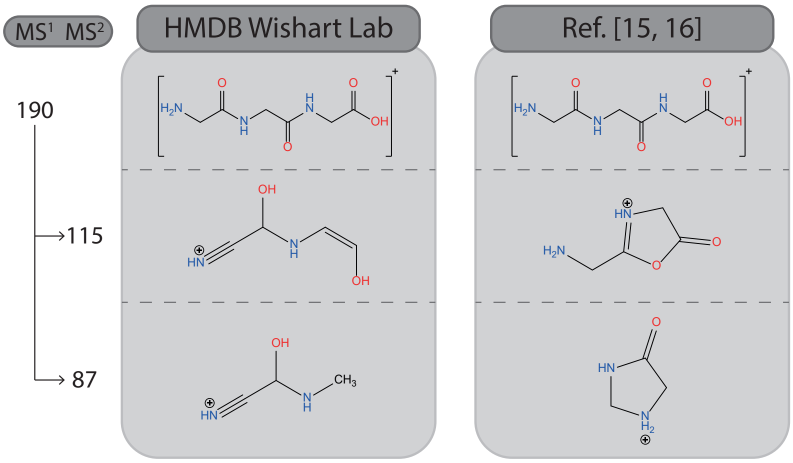

Figure S89: Spectral tree for protonated triglycine with structural annotations given in the HMDB library and structures identified through ion spectroscopy in a previous study [15, 16],

8 Supplementary References

[1] Zhang, P. *et al.* Revisiting Fragmentation Reactions of Protonated alpha-Amino Acids by High-Resolution Electrospray Ionization Tandem Mass Spectrometry with Collision-Induced Dissociation. *Sci. Rep.* **9**, 6453 (2019).

[2] Cai, T. *et al.* Gas-phase intramolecular hydroxyl-amino exchange of protonated arginine and verified by the synthetic intermediate compound. *J. Mass Spectrom.* **53**, 700–704 (2018).

[3] Csonka, I. P., Paizs, B. & Suhai, S. Modeling of the gas-phase ion chemistry of protonated arginine. *J. Mass Spectrom.* **39**, 1025–1035 (2004).

[4] Van Stipdonk, M., Kullman, M., Berden, G. & Oomens, J. IRMPD and DFT study of the loss of water from protonated 2-hydroxynicotinic acid. *Int. J. Mass Spectrom.* **330-332**, 134–143 (2012).

[5] Kempkes, L. J. M., Martens, J., Grzetic, J., Berden, G. & Oomens, J. Deamidation Reactions of Asparagine- and Glutamine-Containing Dipeptides Investigated by Ion Spectroscopy. *J. Am. Soc. Mass Spectrom.* **27**, 1855–1869 (2016).

[6] Kempkes, L. J. M., Martens, J., Berden, G. & Oomens, J. Dehydration reactions of protonated dipeptides containing asparagine or glutamine investigated by infrared ion spectroscopy. *Int. J. Mass Spectrom.* **429**, 90–100 (2018).

[7] Oomens, J. *et al.* Water Loss from Protonated XxxSer and XxxThr Dipeptides Gives Oxazoline-Not Oxazolone-Product Ions. *J. Am. Soc. Mass Spectrom.* **31**, 2111–2123 (2020).

[8] Zou, S., Oomens, J. & Polfer, N. C. Competition between diketopiperazine and oxazolone formation in water loss products from protonated ArgGly and GlyArg. *Int. J. Mass Spectrom.* **316-318**, 12–17 (2012).

[9] Kempkes, L. J. M., Martens, J. K., Grzetic, J., Berden, G. & Oomens, J. Deamidation reactions of protonated asparagine and glutamine investigated by ion spectroscopy. *Rapid Commun. Mass Spectrom.* **30**, 483–490 (2016).

[10] Boles, G. C. *et al.* Ion spectroscopy and guided ion beam studies of protonated asparaginyl-threonine decomposition: Influence of a hydroxyl containing C-Terminal residue on deamidation processes. *Int. J. Mass Spectrom.* **442**, 64–82 (2019).

[11] Kempkes, L. J. M. *et al.* Deamidation of Protonated Asparagine–Valine Investigated by a Combined Spectroscopic, Guided Ion Beam, and Theoretical Study. *J. Phys. Chem. A* **122**, 2424–2436 (2018).

[12] Polfer, N. C., Oomens, J., Suhai, S. & Paizs, B. Infrared spectroscopy and theoretical studies on gas-phase protonated leu-enkephalin and its fragments: Direct experimental evidence for the mobile proton. *J. Am. Chem. Soc.* **129**, 5887–5897 (2007).

[13] Chen, X., Steill, J. D., Oomens, J. & Polfer, N. C. Oxazolone Versus Macrocycle Structures for Leu-Enkephalin b_2-b_4: Insights from Infrared Multiple-Photon Dissociation Spectroscopy and Gas-Phase Hydrogen/Deuterium Exchange. *J. Am. Soc. Mass Spectrom.* **21**, 1313–1321 (2010).

[14] Thevis, M. *et al.* Structure Elucidation of the Diagnostic Product Ion at *m/z* 97 Derived from Androst-4-en-3-One-Based Steroids by ESI-CID and IRMPD Spectroscopy. *J. Am. Soc. Mass Spectrom.* **23**, 537–546 (2012).

[15] Chen, X., Yu, L., Steill, J. D., Oomens, J. & Polfer, N. C. Effect of Peptide Fragment Size on the Propensity of Cyclization in Collision-Induced Dissociation: Oligoglycine b_2-b_8. *J. Am. Chem. Soc.* **131**, 18272–18282 (2009).

[16] Verkerk, U. H. *et al.* a_2 Ion Derived from Triglycine: An N_1-Protonated 4-Imidazolidinone. *J. Phys. Chem. Lett.* **1**, 868–872 (2010).
